# Nutraceuticals in Uro-Oncology: A Structured Expert Review and Precision-Oriented Framework

**DOI:** 10.3390/nu18152411

**Published:** 2026-07-23

**Authors:** Fusun Erten, Ecem Kalemoglu, Omer Kucuk, Kazim Sahin

**Affiliations:** 1 Pertek Sakine Genç Vocational School, Munzur University, 62000 Tunceli, Türkiye; fusunerten@munzur.edu.tr; 2 Department of Medicine, Rutgers-Jersey City Medical Center, Jersey City, NJ 07302, USA; ecem.kalemoglu@rutgers.edu; 3 Winship Cancer Institute, Emory University, Atlanta, GA 30322, USA; 4 Department of Animal Nutrition, Faculty of Veterinary Science, Firat University, 23119 Elazig, Türkiye

**Keywords:** prostate cancer, bladder cancer, renal cell carcinoma, testicular germ cell tumors, nutraceuticals, structured expert review, precision oncology, PI3K/AKT/mTOR, androgen receptor

## Abstract

Urologic malignancies (UMs), including prostate cancer (PCa), bladder cancer (BCa), renal cell carcinoma (RCC), and testicular germ cell tumors (TGCT), are governed by interconnected molecular pathways that regulate proliferation, angiogenesis, metabolism, invasion, immune escape, and treatment resistance. Key pathways include PI3K/AKT/mTOR, VEGF/VEGFR, EGFR, FGFR, c-MET, androgen receptor signaling, DNA damage response, inflammatory transcriptional programs, and regulation of the tumor microenvironment. This structured expert review evaluates mechanistic, translational, epidemiological, and clinical evidence on food- and botanical-derived nutraceuticals that may influence cancer-related signaling, redox balance, inflammation, metabolic adaptation, and host–tumor interactions. Nutraceuticals are considered investigational adjunctive exposures rather than anticancer treatments or alternatives to standard care. Relevant literature was identified through a structured, non-systematic search of PubMed/MEDLINE, Scopus, Web of Science Core Collection, and Embase from database inception to 17 June 2026, supplemented by backward and forward citation searches. Eligible evidence comprised preclinical, observational, interventional, pharmacokinetic, formulation, safety, and drug–nutraceutical interaction studies. Evidence was evaluated by cancer type, compound class, molecular context, formulation, exposure, translational maturity, and safety, and was classified into five stages: prevention signal, mechanistic plausibility, bioavailability and exposure feasibility, exposure-linked biomarker activity, and clinical benefit. An exposure–species–compartment framework was used to assess whether parent compounds and relevant metabolites reached systemic, urinary, or target-tissue concentrations compatible with the proposed effects. Findings based solely on supraphysiological concentrations of unconjugated parent compounds were considered hypothesis-generating unless supported by human exposure or tissue-distribution data. Curcumin, green tea catechins, isoflavones, carotenoids, flavonols, stilbenes, and triterpenoids affect several cancer-related pathways, primarily in experimental models. Translation to clinical practice is constrained by inconsistent formulations, limited bioavailability, inadequate target-tissue exposure data, few biomarker-linked studies, and possible interactions with anticancer therapies. PCa and BCa provide the most suitable settings for exposure-verified mechanistic studies. In RCC, safety and treatment interactions should be prioritized, whereas in TGCTs, non-interference with curative cisplatin-based therapy must be demonstrated. Future studies should use chemically defined formulations, verify clinically relevant exposure, incorporate mechanism-matched biological-response endpoints, and confirm compatibility with established treatment. Current evidence does not support nutraceuticals as treatments for urologic malignancies.

## 1. Introduction

Urologic malignancies, including prostate cancer (PCa), urothelial bladder cancer (BCa), renal cell carcinoma (RCC), and testicular germ cell tumors (TGCT), represent a major component of the global cancer burden. Despite substantial advances in early detection, surgery, targeted therapies, and immunotherapy, metastatic progression and treatment resistance remain significant clinical challenges [1,2]. Increasing evidence indicates that these malignancies function as network diseases, in which tumor behavior is governed by interconnected oncogenic circuits rather than isolated genetic alterations. Across uro-oncology malignancies, several signaling hubs consistently emerge as central regulators of proliferation, survival, metabolic adaptation, angiogenesis, invasion, and therapeutic resistance.

**Figure 1:**
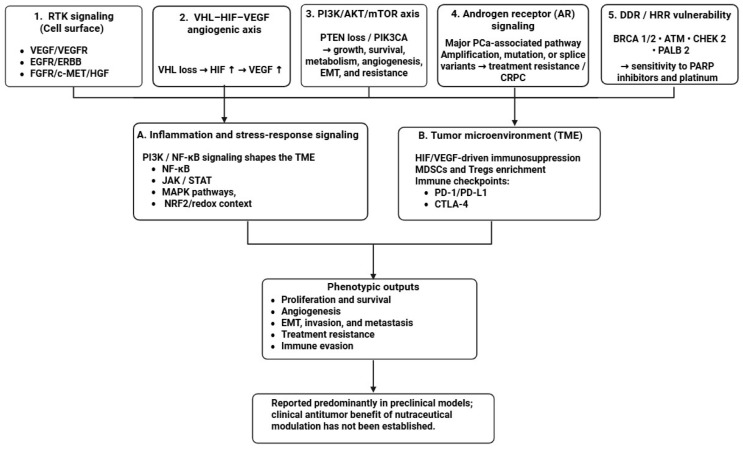
Core signaling hubs potentially modulated by plant-derived nutraceuticals in uro-oncology. The schematic summarizes receptor tyrosine kinase signaling, including VEGF/VEGFR, EGFR/ERBB, FGFR, and c-MET/HGF; the PI3K/AKT/mTOR axis; androgen receptor signaling; the VHL–HIF–VEGF angiogenic program; DNA damage response and homologous recombination repair vulnerabilities; and tumor microenvironmental regulation. The central inflammation and stress-response module encompasses NF-κB-, JAK/STAT-, MAPK-, and PI3K-linked signaling, along with its bidirectional crosstalk with oncogenic pathways and the tumor microenvironment. The lower phenotypic-output module summarizes proliferation and survival, angiogenesis, epithelial–mesenchymal transition, invasion and metastasis, treatment resistance, and immune evasion. Arrows indicate reported or proposed biological relationships and do not imply clinically established modulation by nutraceuticals. Evidence supporting nutraceutical-related modulation of these pathways is predominantly preclinical, and clinical antitumor benefit has not been established.

Among these, the PI3K/AKT/mTOR pathway is one of the most frequently dysregulated networks, promoting cell survival, metabolic reprogramming, angiogenesis, and epithelial–mesenchymal transition (EMT) [3,4]. Its biological relevance is exemplified by extensive crosstalk with androgen receptor (AR) signaling during the development of castration-resistant prostate cancer (CRPC) [5,6]. Additional disease-defining pathways include receptor tyrosine kinase (RTK) signaling, particularly FGFR3 and EGFR alterations in bladder cancer, and VHL loss, leading to activation of the HIF–VEGF angiogenic axis in renal cell carcinoma [7,8]. Furthermore, inflammatory transcriptional programs, DNA damage response (DDR) defects, and tumor microenvironment (TME)-mediated immune suppression significantly influence disease progression and therapeutic responsiveness (Table 1; Figure 1) [9,10].

The extensive crosstalk, feedback regulation, and redundancy within these networks can enable compensatory pathway activation after inhibition of a single molecular target [23,24]. This biological complexity provides a rationale for investigating compounds that can influence multiple disease-relevant processes. However, broad or pleiotropic activity should not be equated with therapeutically meaningful network control. Nutraceuticals frequently alter redox balance, mitochondrial function, inflammatory mediators, apoptosis, and cell-cycle progression, particularly when tested at high concentrations in vitro. Such changes may reflect nonspecific cellular stress or cytotoxicity rather than engagement of a defined oncogenic target. Mechanistic credibility, therefore, requires more than the demonstration of reduced proliferation or increased apoptosis. The parent compound or a biologically active metabolite should reach the relevant biological compartment at an exposure compatible with the reported effect; engagement of a defined molecular node should be demonstrated using target-proximal biochemical, genetic, biophysical, or biological-response evidence; and this engagement should produce a coherent alteration in a disease-relevant signaling circuit. Changes in reactive oxygen species, inflammatory cytokines, apoptosis, migration, or proliferation are therefore interpreted as downstream phenotypic observations unless they are causally linked to the proposed pathway through genetic or pharmacological perturbation, rescue experiments, temporal analysis, orthogonal validation, or exposure-linked pharmacodynamic measurements. Within this framework, polyphenols, flavonoids, carotenoids, stilbenes, and triterpenoids are considered sources of mechanistic and translational hypotheses rather than agents with established clinical efficacy. Preclinical studies suggest that curcumin may influence NF-κB, PI3K/AKT/mTOR, MAPK, STAT3, EMT/MMP, apoptotic, angiogenic, and metabolic signaling; EGCG, genistein, quercetin, resveratrol, lycopene, and ursolic acid have also been linked to AR-, PI3K/AKT/mTOR-, NF-κB-, MAPK-, oxidative stress-, apoptotic-, angiogenic-, and invasion-related pathways in prostate cancer and other urologic cancer models [23,25,26,27,28,29]. Nevertheless, the clinical relevance of these observations depends on whether the biologically active chemical species can be generated at tolerable doses, reach the intended systemic, urinary, or tissue compartment, and produce verifiable target engagement. Translation is further limited by heterogeneous preparations, variable bioavailability, incomplete characterization of active metabolites, inadequate biomarker-guided study designs, and potential nutraceutical–drug interactions [30]. In this review, the terms “modulation,” “biological activity,” and “supportive potential” refer to experimentally or clinically observed changes in physiological, molecular, or biological-response processes. They should not be interpreted as claims that a nutraceutical prevents, treats, or cures cancer. Food- and botanical-derived products may support nutrition or influence redox, inflammatory, metabolic, or immune-related functions, but these effects do not establish antitumor efficacy. Nutraceuticals are therefore evaluated as investigational exposures or potential supportive strategies whose relevance depends on formulation-specific pharmacology, clinically achievable exposure, safety, compatibility with established treatment, and appropriately controlled human evidence. Surgery, radiotherapy, systemic anticancer therapy, and evidence-based supportive oncology remain the standards of care. The central objective is consequently not to catalogue additional pathway effects, but to determine whether a chemically defined preparation can achieve exposure-dependent, disease-relevant biological activity without compromising established oncologic treatment.

## 2. Review Methodology

### 2.1. Review Design and Objectives

This review was developed as a structured expert review rather than a systematic or scoping review. It was therefore not intended to provide an exhaustive inventory of the literature or a quantitatively pooled estimate of treatment effects. The objectives were to identify food- and botanical-derived nutraceuticals relevant to prostate cancer, urothelial bladder cancer, renal cell carcinoma, and testicular germ cell tumors; relate their reported effects to disease-relevant signaling networks; distinguish mechanistic plausibility from bioavailability and exposure feasibility, human biomarker activity, and clinical benefit; and evaluate formulation, pharmacokinetic, safety, and nutraceutical-drug interaction constraints. Because of substantial heterogeneity in compound identity, formulation, dose, exposure, experimental model, biomarker selection, and clinical setting, quantitative meta-analysis was deemed inappropriate. Nevertheless, predefined procedures for literature searching, eligibility assessment, study prioritization, data extraction, evidence classification, and table construction were applied to enhance transparency and consistency in the narrative synthesis.

### 2.2. Information Sources and Literature Search

PubMed/MEDLINE, Scopus, Web of Science Core Collection, and Embase were searched from database inception to 17 June 2026. Search strategies combined controlled vocabulary, where available, with free-text terms covering: (i) prostate cancer, bladder cancer, renal cell carcinoma, and testicular germ cell tumors; (ii) nutraceuticals, phytochemicals, polyphenols, flavonoids, carotenoids, catechins, isoflavones, curcumin, resveratrol, quercetin, genistein, lycopene, epigallocatechin gallate, and triterpenoids; and (iii) cancer-related signaling, pharmacokinetics, bioavailability, metabolism, tissue distribution, safety, and drug interactions.

The principal search concept was: (“prostate cancer” OR “bladder cancer” OR “urothelial carcinoma” OR “renal cell carcinoma” OR “kidney cancer” OR “testicular cancer” OR “testicular germ cell tumor”) AND (nutraceutical* OR phytochemical* OR polyphenol* OR flavonoid* OR carotenoid* OR isoflavone* OR catechin* OR curcumin OR resveratrol OR quercetin OR genistein OR lycopene OR EGCG OR triterpenoid*) AND (signaling OR pathway* OR pharmacokinetic* OR bioavailability OR metabolism OR biomarker* OR safety OR interaction*). Syntax was adapted to the indexing and technical requirements of each database. Additional searches addressed specific compound–cancer combinations, formulation technologies, plasma, urinary, or tissue exposure, clinical trials, and nutraceutical–drug interactions. The reference lists of relevant articles and reviews were examined, and forward citation searches were conducted for influential mechanistic and clinical studies. The complete database-specific strategies are provided in Supplementary Tables S1 and S2.

### 2.3. Eligibility Criteria

Peer-reviewed English-language publications were eligible when they addressed at least one of the four prespecified malignancies and contributed evidence concerning molecular mechanisms, in vivo tumor or microenvironmental effects, epidemiology, human intervention, pharmacokinetics, metabolism, tissue distribution, formulation, pharmacodynamic biomarkers, safety, or nutraceutical–drug interactions. Eligible designs included in vitro, ex vivo, organoid, animal, observational human, pharmacokinetic, and interventional studies. Reviews were used for contextual interpretation and citation tracking, whereas primary studies were prioritized for evidence synthesis and table construction. Publications were excluded if they concerned only non-urologic malignancies without a clear uro-oncology rationale, used inadequately characterized exposures, lacked disease-relevant outcomes, duplicated a more complete report, or were not peer-reviewed.

### 2.4. Study Selection and Prioritization

Records identified through database and citation searches were screened in two stages: title/abstract screening followed by full-text review. As this review was designed as a structured expert synthesis rather than a formal systematic review, the purpose of study selection was not exhaustive inclusion of every publication, but transparent prioritization of the most informative and translationally relevant evidence. Studies were prioritized in the following order: controlled human intervention studies, followed by human pharmacokinetic, tissue-distribution, and biomarker studies, then observational human studies, animal studies, and finally in vitro/ex vivo mechanistic studies. Greater interpretive weight was assigned to studies with well-characterized formulations, justified dosing, verified exposure, disease-relevant pharmacodynamic readouts, and direct implications for clinical trial design.

### 2.5. Data Extraction, Evidence Domains, and Table Construction

For each included study, the following information was extracted where available: cancer type, molecular or histologic context, compound or class, formulation, model or population, dose, route, duration, parent compound or metabolite exposure, tissue or urinary distribution, molecular targets, biomarkers, biological or clinical outcomes, safety findings, and potential interactions with anticancer therapy. Evidence was categorized into human interventional, human observational, pharmacokinetic/formulation, safety/interaction, in vivo preclinical, and in vitro/ex vivo mechanistic domains, and was then mapped to the highest translational stage supported within that specific compound–cancer–formulation context: prevention signal, mechanistic plausibility, bioavailability and exposure feasibility, exposure-linked biomarker activity, or clinical benefit. Tables were constructed using prespecified selection rules. Human evidence tables included only human studies and were organized by cancer type and compound/class. Where multiple studies addressed the same context, priority was given to randomized or controlled studies, studies with exposure verification or tissue/urinary measurements, studies with prespecified pharmacodynamic biomarkers, and studies with interpretable safety data. Where reports were redundant, the most complete or methodologically informative study was retained in the main table, with complementary studies incorporated into the narrative text or Supplementary Material.

### 2.6. Narrative Synthesis and Precision Framework Development

Evidence was synthesized narratively because of substantial heterogeneity across compounds, formulations, doses, endpoints, and clinical settings. Studies were organized by cancer type and interpreted within disease-relevant signaling modules, including androgen receptor signaling, PI3K/AKT/mTOR, receptor tyrosine kinase and MAPK pathways, NF-κB and inflammatory signaling, DNA damage response, HIF-VEGF-mediated angiogenesis, epithelial–mesenchymal transition, apoptosis, metabolism, and immune microenvironmental regulation. Findings derived solely from supraphysiological concentrations of unconjugated parent compounds were interpreted cautiously unless supported by exposure-compatible parent compound or metabolite data, or by formulations achieving verified target-compartment delivery. The precision-oriented framework was developed by integrating disease-specific molecular features, compound class and formulation, exposure feasibility, pharmacodynamic biomarker strategy, safety considerations, and the availability of clinically appropriate trial settings.

### 2.7. Human Evidence Summary and Evidence Hierarchy

Human evidence in uro-oncology remains substantially more limited than the mechanistic and preclinical literature. Table 2 therefore summarizes human studies by cancer type, compound or class, formulation, dose, study design, systemic or target-compartment exposure, biomarker outcomes, clinical findings, safety, and assigned translational stage. Evidence was classified as a prevention signal, mechanistic plausibility, bioavailability and exposure feasibility, exposure-linked biomarker activity, or clinical benefit within each specific compound–cancer–formulation context rather than to a nutraceutical as a whole. The most developed human evidence concerns prostate cancer, particularly standardized green tea catechin interventions in prevention or elevated-risk settings. An early proof-of-principle trial reported fewer prostate cancer diagnoses after one year of green tea catechins at 600 mg/day in men with high-grade prostatic intraepithelial neoplasia, although the small sample size and possible baseline disease imbalance limit generalizability [31,32]. A subsequent randomized, placebo-controlled trial of Polyphenon E providing 400 mg/day of epigallocatechin-3-gallate (EGCG) confirmed measurable exposure, acceptable tolerability, and reduced prostate-specific antigen levels, but did not significantly lower prostate cancer incidence [32]. The ProDiet trial similarly demonstrated adherence, tolerability, and increased circulating EGCG and lycopene-related metabolites, supporting bioavailability and exposure feasibility rather than clinical efficacy [33]. Conversely, a combined lycopene, selenium, and green tea catechin intervention in men with HGPIN and/or atypical small acinar proliferation was associated with more cancers at repeat biopsy and potentially unfavorable microRNA changes [34], illustrating that controlled human findings may diverge from preclinical expectations.

Evidence for other compounds is mainly based on human exposure or exploratory studies. Synthetic lycopene administered with docetaxel and androgen-deprivation therapy was tolerated and produced modest exposure and biomarker changes, but the study was not designed to establish antitumor efficacy [35]. Lipid-enhanced curcumin plus ursolic acid increased circulating ursolic acid exposure without demonstrating tumor response or target-tissue delivery [36]. Curcumin findings require particular caution because enhanced formulations may increase total circulating curcuminoids while free unconjugated curcumin remains below the concentrations commonly used in vitro [37,38].

Outside of prostate cancer, the human evidence base is considerably weaker. Bladder cancer studies remain predominantly preclinical, with few controlled clinical investigations and limited information on urinary exposure, urothelial tissue delivery, or target engagement [39,40]. An uncontrolled LCS103 case series reported reduced recurrence but lacked a comparator, an exposure assessment, and prespecified biomarkers [41]. No sufficiently informative human intervention studies meeting the selection criteria were identified for renal cell carcinoma or testicular germ cell tumors [42,43]. Integrative reviews combining epidemiological, preclinical, and early-phase clinical evidence support further investigation of standardized green tea catechin formulations in active-surveillance settings. However, because these reviews synthesize heterogeneous evidence rather than reporting a single human intervention study, they cannot be assigned a study-level translational stage [44,45]. Overall, current human evidence supports tolerability, exposure feasibility, and limited biomarker activity for selected interventions, but does not establish consistent preventive or therapeutic benefit.

### 2.8. A Five-Stage Translational Maturity Framework for Nutraceutical Evidence

The nutraceutical literature encompasses epidemiological studies, computational analyses, experimental models, human exposure studies, presurgical interventions, and controlled clinical trials. Because these study designs support different levels of inference, they should not be interpreted as equivalent. Cell-culture studies, for example, frequently use micromolar concentrations of unconjugated parent compounds, whereas human administration generally results in substantially lower systemic or tissue exposure and is often dominated by conjugated, methylated, or microbiota-derived metabolites [37,38]. Findings obtained at concentrations incompatible with documented human exposure were therefore considered hypothesis-generating rather than directly translatable.

To provide a consistent basis for interpreting this heterogeneous evidence, we developed a five-stage translational maturity framework. The framework was applied separately to each compound–cancer–formulation context rather than to a nutraceutical as a whole. Table 2 operationalizes this framework for the included human studies by linking study design, exposure verification, biomarker findings, clinical outcomes, and safety data to the highest translational stage directly supported by each study. The assigned stage therefore reflects the strength and type of evidence available for a specific formulation and clinical context and should not be generalized to other preparations, doses, or cancer settings.

Stage 1—Prevention Signal included observational, dietary, prevention, or interception evidence relating an exposure to cancer risk, incidence, or premalignant disease. These associations may justify further investigation but do not establish causality, treatment efficacy, or equivalence between habitual dietary intake and concentrated supplements. Stage 2—Mechanistic Plausibility included in vitro, ex vivo, organoid, and animal evidence demonstrating modulation of a cancer-relevant pathway, cellular process, tumor phenotype, or treatment response. Computational predictions were considered hypothesis-generating unless experimentally validated. Mechanistic evidence was interpreted as target-proximal, circuit-level, or downstream phenotypic. Changes in proliferation, apoptosis, oxidative stress, inflammation, migration, or invasion alone were not considered proof of direct target engagement. Confidence was greater when pathway specificity was supported by genetic or pharmacological perturbation, rescue experiments, temporal analysis, replication across models, and concentrations compatible with human plasma, urinary, or tissue exposure. Stage 3—Bioavailability and Exposure Feasibility required a chemically characterized formulation with acceptable human tolerability and measurable systemic, urinary, or target-tissue exposure. Pharmacokinetic, bioavailability, metabolism, tissue distribution, and preliminary interaction data supported feasibility but not disease-relevant biological activity. Stage 4—Exposure-linked Biomarker Activity required verified exposure to the parent compound or a biologically relevant metabolite together with a prespecified, mechanism-matched pharmacodynamic change in blood, urine, premalignant tissue, or tumor tissue. Nonspecific changes in PSA, oxidative-stress markers, inflammation, apoptosis, or proliferation were insufficient unless prospectively linked to the proposed target or signaling pathway. This stage indicates biological activity in humans, not clinical benefit. Stage 5—Clinical Benefit required controlled evidence of improvement in a validated endpoint, such as cancer incidence, pathological response, recurrence, progression, treatment response, disease-specific survival, or overall survival. Surrogate-marker changes, short-term tissue effects, feasibility outcomes, and uncontrolled observations were not considered evidence of clinical benefit.

**Table 2:** Human evidence for nutraceutical interventions in uro-oncology: study characteristics, exposure verification, biomarker outcomes, clinical findings, safety signals, and assigned translational stage.

Cancer Type	Compound/Class	Dose/Duration	Study Design	Population	Endpoints	Biomarker Outcomes	Clinical Findings	Safety Signals	Assigned Translational Stage
Prostate cancer/premalignant lesions	Green tea catechins	400 mg EGCG/day 1 year	Randomized, placebo-controlled trial	97 men with HGPIN and/or ASAP	1-year PCa rate, PSA, tolerability	Plasma EGCG concentrations were significantly higher in the intervention group, confirming systemic exposure. Serum PSA decreased; the composite endpoint (PCa + ASAP) improved only in men with baseline HGPIN without ASAP.	No significant reduction in 1-year prostate cancer incidence overall despite favorable PSA and subgroup findings	Adverse events did not differ from placebo; well tolerated [32,46]	Stage 3—Bioavailability and exposure feasibility. A standardized formulation achieved measurable plasma exposure and acceptable tolerability. PSA and subgroup findings were not sufficiently mechanism-specific to support Stage 4 and did not establish clinical benefit.
Prostate cancer/premalignant lesions	Green tea catechin preparation	600 mg/day for 1 year	Double-blind, placebo-controlled proof-of-principle study	60 volunteers with HGPIN	Incident PCa, PSA, urinary symptoms, QoL	PSA remained nonsignificantly lower in the GTC arm; no participant-level plasma, urinary, or tissue exposure biomarker was reported.	PCa was diagnosed in 1/30 participants in the GTC group and 9/30 participants in the placebo group.	No significant adverse effects documented [31]	Stage 1—Preliminary prevention signal. The incidence difference supports hypothesis generation, but the small proof-of-principle design and absence of exposure verification preclude classification as Stage 3, Stage 4, or definitive Stage 5 benefit.
Prostate cancer risk/negative biopsy setting	Green tea catechins; lycopene	EGCG 600 mg/day and lycopene 15 mg/day for 6 months	Phase II randomized placebo-controlled factorial trial	Men at increased PCa risk with elevated PSA 2.0–2.95 ng/mL, or PSA 3.0–19.95 ng/mL and a negative prostate biopsy	Feasibility, adherence, exposure biomarkers, safety, and PSA	Plasma EGCG and lycopene metabolite concentrations confirmed adherence to the intervention and systemic exposure.	The study demonstrated feasibility, acceptability, adherence, and systemic exposure but was not powered to assess prostate cancer prevention or clinical benefit.	Interventions were well tolerated; capsules were preferred [33]	Stage 3—Bioavailability and exposure feasibility. Human systemic exposure, adherence, acceptability, and tolerability were demonstrated, but no exposure-linked, mechanism-matched tissue biomarker or clinical benefit was established.
Prostate cancer/premalignant lesions	Lycopene + selenium + green tea catechins	Lycopene 35 mg + selenium 55 μg + green tea catechins (GTCs) 600 mg/day for 6 months	Double-blind randomized controlled trial	60 men with mHGPIN and/or ASAP	Formulation stability, plasma exposure, repeat-biopsy PCa detection, PSA, IPSS, QoL, PR25, and exploratory miRNA profiling	The mean plasma lycopene concentration was 1.45 ± 0.40 μM. Exploratory miRNA profiling included androgen-regulated miR-125b-5p and PTEN-targeting miR-92a-3p; the observed patterns were associated with PCa progression.	More PCa was detected at re-biopsy in the supplementation arm, with borderline significance.	No toxicity above grade 1 occurred during Phase I; one grade 2 abdominal pain event occurred subsequently. The authors concluded that this supplement combination should be avoided [34]	Stage 3—Bioavailability and exposure feasibility, with an unfavorable prevention signal. Formulation stability, plasma exposure, and tolerability were assessed. The exploratory miRNA findings were not prospectively linked to individual-compound exposure and therefore do not meet the criteria for Stage 4.
Prostate cancer/presurgical setting	Quercetin plus green tea extract	Green tea extract 1000 mg/day + quercetin 800 mg/day for 4 weeks before prostatectomy	Translational clinical trial	Men with prostate cancer	Tissue and plasma analytes; enzyme activity	COMT and DNMT enzyme activity, gene expression, and protein expression were evaluated in prostate tissue and red blood cells	Translational exposure study, not efficacy trial	No liver toxicity was observed [47,48]	Stage 3—Bioavailability and exposure feasibility. Plasma, urinary, and prostate-tissue exposure was demonstrated, but no significant mechanism-matched tissue response or clinical efficacy endpoint was established.

Only studies involving human participants are included. Preclinical and mechanistic studies are summarized separately in the cancer-specific sections. Translational maturity was classified according to the five-stage framework described in Section 2.8: Stage 1, prevention signal; Stage 2, mechanistic plausibility; Stage 3, bioavailability and exposure feasibility; Stage 4, exposure-linked biomarker activity; and Stage 5, clinical benefit. Because Table 2 is restricted to human studies, Stage 2 evidence is not presented as a separate study-level category in this table. Prevention signals, bioavailability and exposure feasibility, exposure-linked biomarker activity, and clinical benefit were treated as distinct levels of evidence. A change in a surrogate biomarker or the inclusion of human participants alone was not considered evidence of clinical benefit. Where no sufficiently informative human intervention study was identified, this is stated explicitly to prevent extrapolation from preclinical evidence. The assigned translational stage represents the highest level of evidence directly supported by each study within the specified compound–cancer–formulation context. Stage assignment does not imply that the same level of evidence applies to other formulations, doses, populations, or clinical settings. Plasma EGCG accumulation and acceptable tolerability were demonstrated in the 400 mg/day Polyphenon E study, supporting Stage 3 rather than a prevention-signal-only classification [46]. The combined lycopene–selenium–catechin study verified plasma lycopene exposure but reported exploratory miRNA findings and an unfavorable repeat-biopsy signal; therefore, Stage 3 with an unfavorable prevention signal is the most defensible designation [34]. The quercetin–green tea trial verified plasma, urine, and prostate-tissue exposure but found no significant difference in tissue methylation activity, which also supports Stage 3 rather than Stage 4 [48]. Abbreviations: ASAP, atypical small acinar proliferation; COMT, catechol-O-methyltransferase; DNMT1, DNA methyltransferase 1; EGCG, epigallocatechin-3-gallate; GTCs, green tea catechins; HGPIN, high-grade prostatic intraepithelial neoplasia; IPSS, International Prostate Symptom Score; mHGPIN, multifocal high-grade prostatic intraepithelial neoplasia; miRNA, microRNA; MRP1, multidrug resistance-associated protein 1; PCa, prostate cancer; PK, pharmacokinetics; PR25, European Organisation for Research and Treatment of Cancer prostate cancer-specific quality-of-life module; PSA, prostate-specific antigen; PTEN, phosphatase and tensin homolog; QoL, quality of life.

As summarized in Table 2, the available human evidence for green tea catechins, lycopene, curcumin, ursolic acid, and related interventions predominantly supports prevention signals, bioavailability and exposure feasibility, or, in a limited number of studies, exposure-linked biomarker activity [31,32,33,34,35,36,37,38]. The table also illustrates that the translational stage may differ across studies of the same nutraceutical, as formulation, dose, exposure verification, study population, and endpoint selection vary. No nutraceutical included in Table 2 or elsewhere in the reviewed evidence has demonstrated Stage 5 benefit for the treatment of a uro-oncologic malignancy [31,32,33,34,35,36,37,38,39,40,41]. Overall, the broader evidence base remains concentrated in Stages 1 and 2, whereas the human studies presented in Table 2 primarily support Stage 3 and offer limited conclusions for Stage 4. Accordingly, all conclusions were restricted to the highest stage directly supported within each compound–cancer–formulation context and should not be interpreted as evidence of clinical readiness or as a recommendation for patient use.

## 3. Uro-Oncology as a Network Disease: Central Signaling Hubs and Translational Implications

Although prostate, bladder, renal, and testicular malignancies arise from distinct tissues and are driven by different initiating molecular events, increasing evidence suggests that common systems-level principles govern their clinical behavior. Rather than functioning as collections of independent oncogenic alterations, these tumors evolve through interconnected signaling, metabolic, genomic, and microenvironmental networks that collectively regulate proliferation, survival, angiogenesis, immune evasion, and therapeutic responsiveness [3,4,49]. This network architecture provides a biological basis for tumor plasticity and helps explain why durable responses are often difficult to achieve despite increasingly sophisticated targeted therapies.

A hallmark of network-driven tumor biology is signaling convergence. Diverse upstream molecular abnormalities frequently funnel into a limited number of shared biological outputs that sustain malignant growth and adaptation. Consequently, inhibition of a dominant signaling node rarely produces complete pathway suppression. Instead, compensatory activation of parallel circuits, feedback regulation, and pathway redundancy enable tumor cells to preserve essential functions under therapeutic pressure. Resistance therefore emerges not merely as a consequence of individual molecular alterations but as an adaptive property of the network itself [4,11,17,49,50,51,52,53,54].

The complexity of these regulatory systems extends beyond canonical oncogenic pathways. Genomic instability and defects in DNA damage response (DDR) mechanisms generate molecular heterogeneity while simultaneously creating therapeutically exploitable vulnerabilities. Alterations in homologous recombination repair genes, including BRCA1, BRCA2, ATM, and CHEK2, exemplify this principle and have enabled some of the most successful applications of precision oncology through PARP inhibitors and platinum-based therapies [10,12]. Increasing evidence further suggests that DDR dysfunction influences inflammatory signaling, immune recognition, and treatment responsiveness, reinforcing its integration within broader oncogenic networks.

Equally important is the contribution of the tumor microenvironment (TME), which functions as a dynamic regulatory compartment rather than a passive bystander. Interactions among stromal cells, immune populations, inflammatory mediators, and angiogenic signals actively shape tumor evolution and therapeutic response. Hypoxia-driven remodeling, aberrant vascularization, and the recruitment of immunosuppressive cell populations establish a microenvironment that facilitates tumor adaptation and immune escape. As a result, responsiveness to targeted therapies and immune checkpoint blockade is determined not only by tumor-intrinsic alterations but also by the broader ecological context in which malignant cells reside [4,9,11,17].

**Figure 2:**
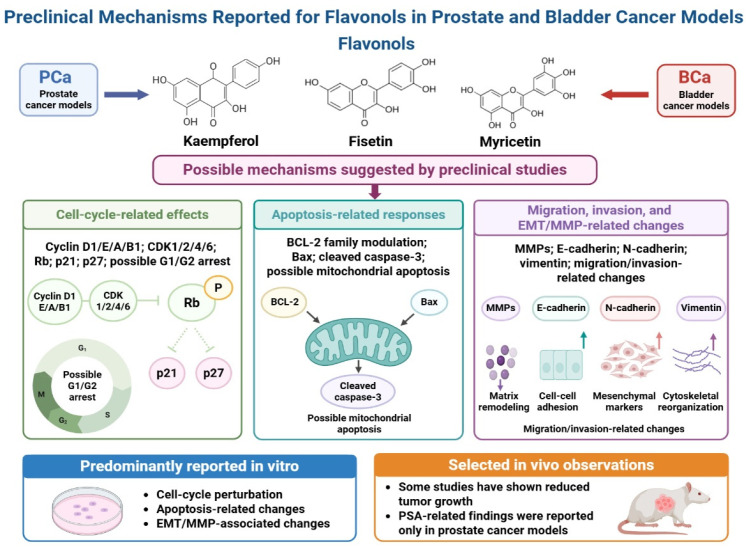
Possible mechanisms reported for kaempferol, fisetin, and myricetin in preclinical prostate and bladder cancer models. In vitro studies suggest that these flavonols may influence cell-cycle regulation, mitochondrial apoptosis, and epithelial–mesenchymal transition- and matrix metalloproteinase-associated processes. Some preclinical studies have shown increased BAX and cleaved caspase-3, reduced BCL-2-family signaling, and changes in E-cadherin, *N*-cadherin, vimentin, and MMP expression. Selected in vivo studies have reported reduced tumor growth, whereas reductions in PSA or AR-related activity have been described only in prostate cancer models. In bladder cancer models, reported effects primarily involve proliferation, migration, invasion, and EMT-related processes. Evidence for enhanced responses in combination experiments remains preclinical and does not establish clinical anticancer efficacy.

Recent advances have revealed additional layers of regulation that further contribute to network behavior. Epitranscriptomic modifications, translational control mechanisms, metabolic stress-response pathways, and non-coding RNA networks influence cellular adaptation and therapeutic resistance independently of new genomic alterations [1,15,55,56]. Dysregulated RNA-processing machinery, translational regulators such as eIF4E, long non-coding RNAs, and metabolic defense systems including the glyoxalase pathway collectively contribute to the maintenance of malignant phenotypes and adaptive survival programs [13,15,55,56]. Collectively, these observations support a transition from reductionist models of tumor biology toward a systems-oriented view of uro-oncology. Within this framework, effective therapeutic intervention increasingly depends on the ability to influence multiple biologically relevant nodes simultaneously rather than targeting individual pathways in isolation. This systems-oriented concept does not imply that every nutraceutical affecting multiple pathways is a network-modulating agent. Here, “network modulation” requires exposure to a parent compound or active metabolite, evidence of target engagement, and a coherent change in a disease-defining circuit, such as the AR–PI3K axis in selected prostate cancers, FGFR3/ERBB–PI3K signaling in bladder cancer, VHL–HIF–VEGF signaling in clear-cell renal cell carcinoma, or DNA damage- and cisplatin-response pathways in testicular germ cell tumors (Table 1). Isolated changes in apoptosis, oxidative stress, inflammation, mitochondrial function, or proliferation are considered downstream observations unless causally linked to the relevant oncogenic network through pathway perturbation, rescue experiments, orthogonal validation, temporal analysis, and clinically achievable tissue exposure. Nutraceuticals should therefore be viewed as context-dependent modulators rather than broad inhibitors of multiple oncogenic pathways. Their translational relevance depends on measurable, exposure-dependent modulation of a tumor-relevant circuit. Future studies should integrate molecular subtype selection, characterization of parent compounds and active metabolites, tissue-exposure verification, pathway-specific pharmacodynamic biomarkers, and mechanism-based clinical trial designs. Flavonols, for example, affect cell-cycle, mitochondrial-apoptotic, and epithelial–mesenchymal transition pathways in experimental prostate and bladder cancer models, but these findings remain preclinical and require confirmation at clinically achievable exposures (Figure 2).

## 4. Cancer-Specific Mechanistic Integration and Translational Boundaries

A disease-specific assessment of nutraceuticals should begin with the molecular and physiological context in which a compound has been studied, while avoiding the assumption that pathway modulation constitutes cancer treatment. Across prostate cancer, bladder cancer, renal cell carcinoma, and testicular germ cell tumors, plant-derived compounds have been reported to influence redox regulation, inflammatory signaling, metabolic processes, apoptosis, angiogenesis, and immune-related pathways [29,39,40,43,57]. Most such observations originate from in vitro or animal studies and should therefore be interpreted as mechanistic plausibility or potential supportive biological activity rather than therapeutic efficacy. For this review, a nutraceutical is considered translationally informative only when a chemically characterized preparation achieves measurable exposure to the relevant parent compound or metabolite, produces a prespecified pharmacodynamic effect, has an acceptable safety profile, and does not interfere with established oncologic treatment. Changes in proliferation, apoptosis, oxidative stress, cytokines, migration, or invasion alone are insufficient to support a disease-treatment claim. The following sections therefore identify research hypotheses and stage-appropriate supportive or investigational settings rather than recommending nutraceuticals for cancer treatment.

A rational nutraceutical strategy in uro-oncology should be anchored to a molecularly defined tumor state and its dominant signaling circuitry rather than to generic antioxidant, anti-inflammatory, or pro-apoptotic activity. Across prostate cancer, bladder cancer, renal cell carcinoma, and testicular germ cell tumors, biological relevance is determined by distinct combinations of lineage dependence, receptor tyrosine kinase signaling, PI3K/AKT/mTOR activation, DNA damage response, inflammatory transcription, angiogenesis, metabolic adaptation, lineage plasticity, and immune regulation. To avoid equating broad pleiotropy with meaningful network control, the evidence discussed in this section is interpreted at three levels. Target-proximal evidence demonstrates engagement of a defined molecular node by the parent compound or an active metabolite. Circuit-level evidence demonstrates that this engagement modifies a disease-defining signaling program, including its downstream transcriptional output, feedback architecture, or adaptive response. Downstream phenotypic evidence comprises changes in apoptosis, oxidative stress, inflammation, proliferation, migration, or invasion without sufficient proof of the initiating target or circuit. Only candidates supported by exposure-compatible target-proximal or circuit-level evidence should be prioritized for translational development. Compounds supported primarily by downstream phenotypic findings should remain hypothesis-generating, irrespective of the number of cellular pathways reported to change. Accordingly, the most credible candidates are those for which a chemically characterized preparation can achieve measurable systemic, tissue, or urinary exposure; produce a compound-specific target-engagement signature; and alter a clinically relevant pathway in a molecularly selected disease context. This framework is operationalized in Table 3, which summarizes candidate nutraceutical contexts, evidence maturity, key translational limitations, and the earliest justified research steps across uro-oncology malignancies.

Throughout the following cancer-specific sections, the terms “clinical evidence,” “human evidence,” and “biological activity” are used only with an explicit indication of the inference supported by the relevant study design. Human participation alone does not establish clinical benefit. A pharmacokinetic study may support Stage 3 feasibility, whereas a presurgical study may support Stage 4 biomarker activity only when compound exposure and a prespecified pathway-matched biomarker are evaluated together. Conversely, epidemiological associations are framed as prevention signals, and cell-line or animal findings are framed as mechanistic plausibility. This distinction is maintained even when several evidence types are available for the same compound.

### 4.1. Prostate Cancer

Among the uro-oncology malignancies considered in this review, prostate cancer (PCa) provides the most tractable, although not clinically validated, framework for mechanism-based nutraceutical research. This relative advantage reflects both the central role of androgen receptor (AR) signaling in disease biology and the availability of clinically accessible settings, including active surveillance, biochemical recurrence, and presurgical intervention windows, in which systemic and prostate-tissue exposure, target engagement, and pharmacodynamic responses can be assessed [5,53]. Fontana et al. [26] described the capacity of natural compounds to influence AR signaling, as well as PI3K/AKT/mTOR, inflammatory, apoptotic, and metabolic pathways. More recent reviews by Wu et al. [144] and Pratama et al. [145] indicate that selected natural products may affect the AR axis through receptor antagonism or degradation, inhibition of nuclear translocation and transcriptional activity, and modulation of treatment-resistant AR variants. Tyagi et al. [146] further identified AR splice variants as potential nutraceutical targets in castration-resistant PCa. Nevertheless, these observations remain predominantly preclinical, and their relevance at achievable concentrations in human prostate tissue has not been established.

PCa progression extends beyond a purely AR-driven model and involves interactions among AR-dependent transcription, PI3K/AKT/mTOR and MAPK signaling, inflammatory and redox pathways, metabolic adaptation, DNA-damage responses, epithelial–mesenchymal transition, and lineage plasticity [4,5,53,147]. Natural compounds may influence several of these networks simultaneously rather than acting through a single molecular target. Besasie et al. [148], Chopra et al. [149], and Kallifatidis et al. [150] emphasized that the biological effects of nutraceuticals may arise from coordinated modulation of hormonal, proliferative, inflammatory, metabolic, and stress-response programs. Curcumin and ursolic acid, for example, converge on NF-κB, AKT, STAT3, mTOR, and AR-associated signaling rather than acting through an isolated target [148]. Such pleiotropy may be relevant in a biologically redundant tumor, but it does not automatically confer meaningful network control and may also lead to compensatory signaling, pharmacological antagonism, or context-dependent effects.

Molecular heterogeneity is therefore central to the interpretation of nutraceutical activity in PCa. Luminal and basal transcriptional states differ in AR dependence, differentiation, prognosis, and response to androgen-deprivation therapy [151,152]. AR-high luminal tumors may provide a more coherent context for compounds proposed to modulate AR signaling, whereas basal-like and lineage-plastic tumors may require interpretation in the context of altered proliferation programs, reduced AR dependence, epithelial plasticity, neuroendocrine differentiation, and treatment-adaptive transcriptional states. Neuroendocrine PCa represents a particularly distinct, frequently treatment-emergent state characterized by reduced AR dependence and extensive epigenetic and transcriptional reprogramming. Single-cell analyses support luminal-to-neuroendocrine transdifferentiation during disease progression [153]. Findings obtained in AR-positive adenocarcinoma models should consequently not be generalized to AR-low, basal-like, lineage-plastic, or neuroendocrine disease. Even within AR-active tumors, PTEN loss, PI3K activation, DNA-repair alterations, prior hormonal treatment, and disease stage may modify biological responses [5,53]. Claims of AR-related modulation should therefore be supported by comparisons between AR-dependent and AR-independent models, assessment of AR nuclear localization and transcriptional output, and, where relevant, measurement of AR splice variants. Genetic perturbation, target-specific rescue, pharmacological comparators, and testing at exposure-compatible concentrations are required to distinguish modulation of an AR-defined circuit from generalized oxidative stress, apoptosis, or growth inhibition.

Mechanistic claims in PCa must also be interpreted according to the chemical species that actually reach prostate tissue. Plasma exposure alone cannot establish prostate delivery, and detection of total compound-derived material does not indicate that the unconjugated parent molecule is present. Accordingly, the translational relevance of AR-, PI3K-, NF-κB-, apoptosis-, or redox-related effects depends on whether the parent compound or a biologically active metabolite reaches prostate tissue at a concentration compatible with the proposed mechanism. Human presurgical studies that quantify parent compounds and metabolites in plasma, urine, and prostate tissue therefore provide a more informative basis for mechanistic interpretation than administered dose or plasma concentration alone.

Soy-derived isoflavones, including genistein, daidzein, and the microbiota-derived metabolite S-equol, are among the most extensively studied nutraceuticals in PCa. Experimental evidence indicates that isoflavones can suppress AR transcriptional activity, reduce AR-regulated targets such as PSA, and inhibit proliferation in androgen-sensitive and castration-resistant models [26,61]. Genistein also inhibits IGF-I-mediated PI3K/AKT and MAPK signaling [67], whereas S-equol has been linked to AKT–FOXO3a modulation, reduced MDM2 expression, G2/M arrest, and apoptosis [64]. These findings support an interaction between AR-dependent transcription and PI3K-linked survival signaling rather than an isolated phytoestrogenic effect.

Human findings, however, remain inconsistent. Higher soy intake, particularly in East Asian populations, has been associated with lower PCa incidence, suggesting that long-term dietary exposure may be more relevant to disease interception than to the treatment of established cancer [60]. Short-term intervention studies have reported reduced PSA after genistein supplementation in some settings [63], whereas other studies have demonstrated increased circulating genistein and daidzein concentrations without significant changes in PSA [59]. Soy protein isolate did not reduce biochemical recurrence after prostatectomy, and high-dose isoflavones did not improve metabolic or inflammatory outcomes during androgen deprivation therapy [58,65]. These differences may reflect variation in disease stage, formulation, exposure duration, baseline diet, endpoint selection, and metabolic phenotype rather than a simple distinction between effective and ineffective interventions.

Purified genistein, mixed isoflavone supplements, soy protein isolates, and whole-soy foods are not pharmacologically equivalent. In addition, only a proportion of individuals efficiently convert daidzein to S-equol, and microbiome-dependent equol production may substantially influence systemic and tissue exposure [62,66,154]. The relationship among microbiota composition, inflammatory tone, and nutraceutical metabolism may further modify responses in PCa [155]. Trials that combine equol producers and non-producers without metabolite quantification may therefore obscure effects confined to metabolically defined subgroups. Changes in PSA alone should also not be regarded as evidence of tumor regression or pathway-specific target engagement. Future studies should stratify participants by equol-producer status, quantify parent compounds and metabolites, measure prostate-tissue exposure, and incorporate biomarkers of AR, PTEN, PI3K, and DNA repair. The clinically relevant exposure should therefore be defined separately for genistein, daidzein, S-equol, and other circulating metabolites rather than expressed as total isoflavone intake. Mechanistic effects demonstrated with unconjugated genistein or daidzein should not be extrapolated to all supplemented individuals unless the corresponding parent compound or active metabolite is detected in prostate tissue at an exposure-compatible concentration. Equol-producer status should be incorporated prospectively because it represents a qualitative metabolic phenotype that may determine both the chemical species reaching the prostate and the resulting pharmacodynamic response [154].

A practical early-stage isoflavone study would prospectively identify equol producers using standardized serum and urinary measurements of daidzein and S-equol. Yoshikata et al. [156] demonstrated that equol-producer status can be characterized by LC–MS/MS and confirmed substantial interindividual variation in circulating and urinary equol levels. Akaza et al. [157] reported a lower frequency of equol producers among men with PCa than among controls, although this association does not establish causality or clinical benefit. Candidate studies should preferentially enroll men with localized, AR-active adenocarcinoma in active-surveillance or presurgical settings. A chemically defined preparation should be used, with separate quantification of genistein, daidzein, S-equol, and their major metabolites in serum, urine, and, where available, prostate tissue. A prespecified tissue measure of AR activity, such as AR nuclear localization or a validated AR-transcriptional signature, would be more informative as a primary pharmacodynamic endpoint than serum PSA alone. AR-regulated genes, including KLK3, may serve as supporting endpoints, whereas *p*-AKT or *p*-S6 should be included only when baseline profiling demonstrates PTEN loss or PI3K Pathway activation [53]. Itsumi et al. [158] found that equol reduced AR and PSA expression more prominently in androgen-dependent LNCaP cells than in castration-resistant models, supporting the importance of disease context. However, experimental concentrations must be compared directly with measured human tissue exposure before AR modulation can be considered pharmacologically plausible. Miyanaga et al. [159] observed no significant reduction in overall cancer detection with purified isoflavones, although exploratory findings suggested possible age-related heterogeneity. A convincing proof-of-mechanism result would therefore require verified S-equol exposure, an AR-active baseline tumor state, and a coherent change in tissue AR activity. An isolated PSA change without tissue exposure or pathway modulation should remain exploratory.

**Figure 3:**
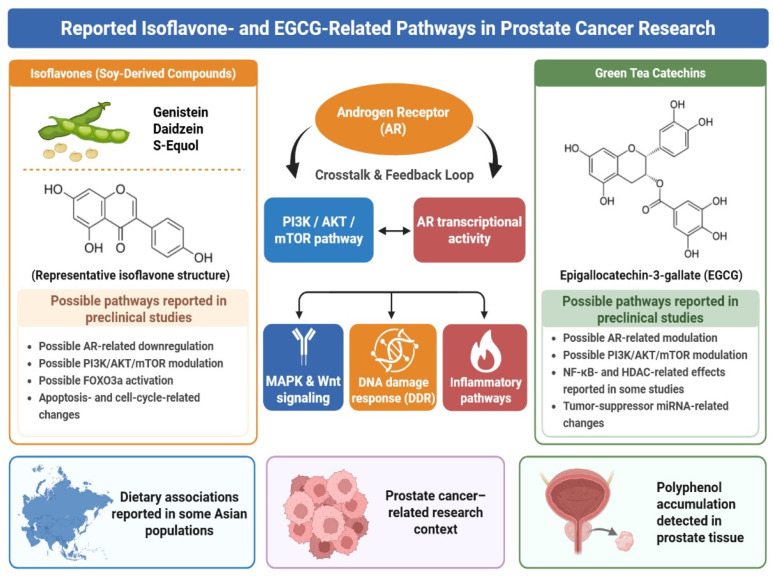
Reported isoflavone- and EGCG-related pathways in prostate cancer research. The chemical structures of genistein, daidzein, S-equol, and (−)-epigallocatechin-3-gallate are shown. Preclinical studies suggest that these compounds may influence androgen receptor signaling, PI3K/AKT/mTOR, MAPK/WNT, DNA damage response, and inflammatory pathways. Epidemiological studies have reported population-dependent associations with prostate cancer risk, whereas short human interventions have mainly evaluated exposure and exploratory biomarkers. The pathways illustrated represent mechanistic hypotheses and do not establish clinical treatment efficacy. Unless otherwise specified, the illustrated effects are derived predominantly from experimental studies and represent mechanistic or potentially supportive biological activity rather than established tumor-directed efficacy or clinical recommendations.

Green tea catechins, particularly epigallocatechin-3-gallate (EGCG), represent another mechanistically attractive group of compounds. EGCG influences proliferation, inflammation, angiogenesis, and therapy resistance by modulating AR, PI3K/AKT, NF-κB, MAPK, and STAT signaling [29,139]. EGCG has been shown to directly alter AR ligand binding and AR-dependent transcription, leading to deregulation of androgen-driven tumor growth [75]. In preclinical models, EGCG inhibits tumor expansion, reduces angiogenesis, promotes apoptosis, and attenuates stem-like phenotypes; similar effects have been observed in androgen-independent xenografts and in the TRAMP model, where green tea polyphenols delayed tumor formation and prolonged tumor-free survival, particularly when administered early during carcinogenesis [68,72]. Other mechanistic studies proposed that EGCG can also modulate androgen biosynthesis via interaction with CYP17A1 [69,73,74], with further epigenetic effects, such as HDAC inhibition and upregulation of tumor-suppressive microRNAs, such as miR-34a, expanding its potential influence [70,71,76] (Figure 3). Together, the principal isoflavone- and EGCG-related mechanistic hypotheses discussed above are summarized in Figure 3.

Human prostate-tissue data provide partial evidence of bioavailability and exposure feasibility but do not yet establish pathway-specific activity. In the presurgical study by Henning et al. [48], EGCG and epicatechin gallate were detectable in prostate tissue in most participants receiving green tea extract, demonstrating that selected catechins can reach the prostate. Quercetin co-administration substantially increased plasma, urine, and prostate-tissue quercetin exposure; however, it did not significantly increase prostate-tissue green tea polyphenol concentrations or alter the measured COMT- and DNMT-related methylation endpoints. Tissue detection should therefore be interpreted as evidence of delivery rather than evidence that AR-, PI3K-, NF-κB-, or epigenetic effects occur at biologically meaningful levels. Future studies should relate individual catechin and metabolite concentrations in prostate tissue to prespecified target-proximal and circuit-level pharmacodynamic endpoints.

Curcumin illustrates both the appeal and limitations of network-oriented nutraceutical research. It has been reported to influence NF-κB, PI3K/AKT/mTOR, JAK/STAT, MAPK, WNT/β-catenin, AR-associated transcription, inflammatory mediators, metabolic regulation, and apoptosis-related pathways [25,84,90,96,97]. Curcumin and ursolic acid may converge on AKT, STAT3, mTORC1, mitochondrial function, cell-cycle regulation, the unfolded-protein response, and apoptosis-related pathways [88]. In transgenic PCa models, combined administration was associated with greater inhibition of tumor progression than either compound alone and with coordinated changes in several pathways [88]. These observations remain preclinical and should not be interpreted as evidence of a clinically effective combination. A major limitation is that many curcumin studies use micromolar concentrations of the unconjugated parent compound, which are difficult to reproduce in human plasma or prostate tissue [37,38]. Poor absorption, rapid metabolism, and extensive conjugation remain substantial barriers. Nanoparticle, liposomal, and other enhanced-delivery systems may improve solubility, cellular delivery, release behavior, or in vitro anticancer activity in experimental models [85,95], but formulation enhancement alone does not demonstrate clinical applicability. Each preparation requires independent characterization of composition, stability, parent-compound and metabolite pharmacokinetics, tissue distribution, safety, and interaction potential with AR-directed and other systemic therapies. Enhanced-delivery approaches should therefore be evaluated as tools for closing a predefined pharmacokinetic–pharmacodynamic gap rather than as evidence of efficacy [160,161].

**Figure 4:**
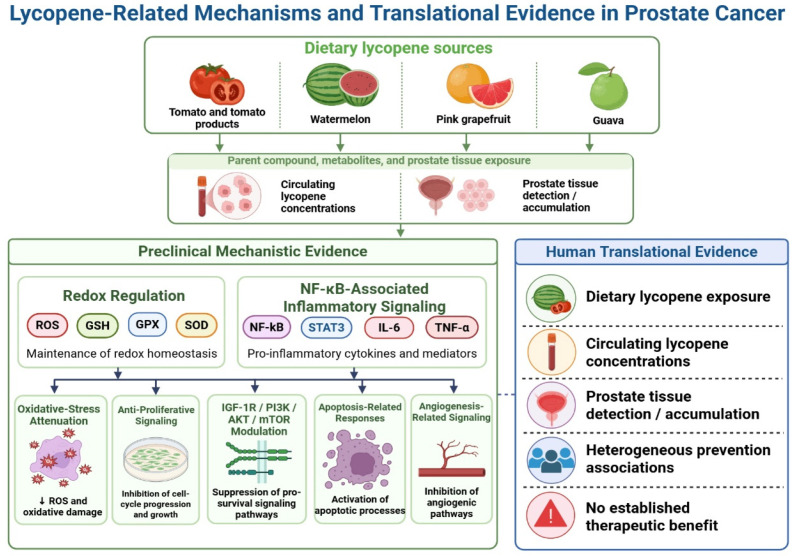
Dietary sources, possible mechanisms, and translational evidence for lycopene in prostate cancer. The illustrated dietary sources are tomato and tomato products, watermelon, pink grapefruit, and guava. Preclinical PCa studies suggest that lycopene may modulate oxidative stress regulation, NF-κB-associated inflammatory signaling, IGF-1/PI3K/AKT signaling, cell-cycle control, apoptosis, and angiogenesis. Human evidence consists mainly of dietary and observational associations, exposure studies, and limited interventions and remains heterogeneous. The figure is restricted to prostate cancer and does not imply interventional effects in bladder cancer, renal cell carcinoma, or testicular germ cell tumors. The illustrated pathways represent mechanistic and prevention-related hypotheses rather than established therapeutic efficacy.

Lycopene occupies a distinct position within the evidence landscape because its strongest rationale centers on dietary exposure, risk-related associations, and early disease interception rather than on the treatment of established or advanced PCa. Preclinical studies suggest that lycopene may influence redox-sensitive signaling, inflammatory mediators, IGF-1/PI3K/AKT activity, cell-cycle regulation, mitochondrial apoptosis, and angiogenesis [77,78,79,81,82]. Prospective epidemiological and intervention findings, however, remain heterogeneous and may differ by dietary source, food matrix, baseline exposure, dose, and prostate tissue uptake [77,78,79,80,81,82,83]. Tomato-derived foods, isolated lycopene preparations, and enhanced-bioavailability formulations should therefore not be regarded as pharmacologically equivalent. Lycopene sources and possible PCa-related mechanisms are summarized in Figure 4, without implying effects in BCa, RCC, or TGCTs, or established clinical benefit.

Fisetin, kaempferol, myricetin, quercetin, resveratrol, and other flavonoids have also been reported to influence AR, PI3K/AKT, NF-κB, STAT3, EMT, apoptosis, migration, and invasion in experimental PCa models [86,89,91,94,162]. Their evidence base remains predominantly preclinical, and apparent multitarget activity should be interpreted in light of limited human exposure data and the extensive metabolism of these compounds. Reductions in tumor growth or PSA reported in selected animal PCa models should not be extrapolated to clinical efficacy (Figure 2).

Resveratrol provides a clear example of the discrepancy between parent-compound mechanisms and human tissue exposure. In a presurgical radiolabeled study, Cai et al. [163] detected resveratrol-derived material in human prostate tissue after both dietary-achievable and pharmacological oral doses, but unconjugated resveratrol was not detected; sulfate and glucuronide conjugates predominated. Antiproliferative effects in PCa cells generally required free resveratrol concentrations of approximately 5–10 μM or greater, while many experimental studies used 25–100 μM. The tested conjugates did not consistently suppress prostate cell growth, potentially due to limited cellular uptake. Resveratrol-related claims should therefore be considered translationally plausible only if activity is demonstrated for tissue-relevant conjugates, local regeneration of the parent compound is verified, or a formulation achieves greater, safe, and measurable prostate exposure. Similar considerations apply to other flavonoids. Liu et al. [164] showed in rats that orally administered flavones undergo extensive transformation and that prostate distribution includes both parent and metabolized species. Although these findings cannot be directly extrapolated to humans, they reinforce the need to investigate methylated, glucuronidated, and other circulating metabolites rather than relying solely on unconjugated compounds tested in vitro. Human studies should quantify individual prostate-tissue species and compare their concentrations with metabolite-specific concentration–response relationships.

Pomegranate-derived preparations further illustrate the importance of microbiota-dependent metabolism. Ellagitannins are extensively transformed after ingestion, and urolithins may represent more relevant circulating and tissue-exposed species than the parent compounds. Seeram et al. [165] detected ellagitannin-derived metabolites in mouse prostate tissue and reported inhibition of PCa-cell growth, while a testosterone-induced rat BPH model associated pomegranate waste extract exposure with modulation of NRF2, HO-1, IL1R1, mitochondrial, redox, and DNA-repair-related proteins [166]. These findings support further investigation of urolithins but do not establish equivalent exposure or biological activity in human prostate tumors. Future studies should consider urolithin metabotype and quantify individual metabolites in plasma and prostate tissue before attributing NRF2-, inflammatory-, mitochondrial-, or DNA-repair effects to the administered preparation.

Redox and inflammatory findings require particularly cautious interpretation. NF-κB/p65 may suppress NRF2-dependent transcription by competing for transcriptional coactivators and recruiting repressors, whereas NRF2 can inhibit selected pro-inflammatory programs [167,168]. Transient NRF2 activation may support detoxification or protect non-malignant tissue from oxidative injury. In established tumors, however, sustained NRF2 activity may enhance antioxidant capacity, metabolic adaptation, tumor survival, and resistance to treatment-induced stress [169,170]. Changes in NRF2, HMOX1, NQO1, or total antioxidant capacity should therefore not automatically be classified as favorable tumor-related effects. Studies should establish the duration of the response, the malignant or non-malignant compartment involved, and the consequences for sensitivity to standard treatment. Overall, PCa offers a practical setting for nutraceutical pharmacology, as molecularly defined subgroups and active surveillance or presurgical windows permit direct evaluation of prostate-tissue exposure and pathway-specific responses. This research accessibility should not be confused with clinical readiness. Even for extensively studied compounds such as isoflavones, EGCG, curcumin, lycopene, and resveratrol, human studies have not demonstrated consistent tumor-directed clinical benefit. Future investigations should distinguish hypotheses related to disease prevention from those concerning already established disease; account for AR activity, luminal or basal state, lineage plasticity, PTEN/PI3K status, DNA-repair alterations, previous therapy, redox context, and microbiome-dependent metabolism; and use chemically characterized formulations with reproducible dose–exposure relationships. Progression to controlled biomarker studies should require identification of the biologically relevant parent compound or metabolite, adequate prostate-tissue exposure, acceptable safety, and subtype-matched target engagement. Clinical outcome-oriented adjunct or recurrence-prevention studies remain premature until reproducible exposure-linked biological activity has been demonstrated in human prostate tissue.

### 4.2. Bladder Cancer

Bladder cancer (BCa) is a molecularly heterogeneous disease in which receptor tyrosine kinase signaling, PI3K/AKT/mTOR activation, tumor-suppressor alterations, inflammatory pathways, and immune–stromal interactions contribute to disease progression and treatment response [4,7,11,171]. In the context of nutraceutical research, however, translational relevance depends less on cataloging the multiple pathways affected in experimental models than on demonstrating that a chemically defined preparation achieves biologically relevant exposure in urine and bladder tissue. The anatomical accessibility of the bladder makes short pre-transurethral resection of bladder tumor (TURBT) or peri-cystectomy studies particularly suitable for investigating target-compartment exposure and tissue biological responses. This accessibility facilitates translational assessment but, by itself, does not indicate clinical readiness or therapeutic efficacy.

A compartment-specific exposure framework is essential because urinary excretion may result in repeated luminal contact between phytochemical-derived chemical species and the urothelium. Timed urine, plasma, and, where feasible, paired bladder mucosa or tumor tissue should therefore be analyzed for the administered parent compound and relevant conjugated, microbiota-derived, or degradation metabolites [39,172]. The concentration and duration of exposure, urinary pH, chemical stability, deconjugation, tissue permeability, and intracellular availability should also be considered. Detection in urine confirms luminal exposure but does not establish intracellular tumor delivery or pathway engagement. Consequently, claims involving FGFR/ERBB–PI3K, NF-κB, mTOR, redox regulation, apoptosis, or related processes require evidence that the relevant chemical species reaches bladder tissue and produces a prespecified, mechanism-matched tissue response. Developments in molecularly selected urothelial cancer research demonstrate that such studies are methodologically feasible. FGFR-directed treatment strategies have established the biological and clinical relevance of FGFR signaling while also revealing the importance of patient selection, pathway bypass, and acquired resistance [109,173]. Biomarker-guided neoadjuvant and adjuvant studies further support molecularly informed study designs [174,175], and longitudinal urinary tumor-DNA assessment during presurgical immunotherapy shows that urine-based molecular monitoring can be integrated into short intervention windows [176]. These developments support the feasibility of subtype-selected exposure and pharmacodynamic studies but do not provide evidence that nutraceuticals improve clinical outcomes.

Molecular selection is particularly important because contemporary classifications distinguish luminal papillary, luminal nonspecified, luminal unstable, stroma-rich, basal/squamous, and neuroendocrine-like phenotypes, each with distinct receptor tyrosine kinase activity, tumor-suppressor alterations, differentiation programs, and immune–stromal characteristics [177,178,179,180]. FGFR3 alterations are enriched in luminal-papillary disease and can activate MAPK and PI3K/AKT/mTOR signaling; however, FGFR3 status alone may be insufficient for study selection because downstream effectors, alternative receptor tyrosine kinases, and adaptive signaling can maintain pathway activity [181,182,183]. FGFR3 mutation, fusion, or validated overexpression may therefore serve as an eligibility or enrichment criterion, but it should be accompanied by baseline confirmation that the proposed downstream pathway is active. The earliest justified human investigation would be a short, formulation-specific presurgical study integrating molecular context with exposure verification and one prespecified pathway-related endpoint. Iida et al. showed that luteolin inhibited BCa growth in experimental models by modulating mTOR-related signaling and inducing p21 [184]. Although these findings support mechanistic plausibility, they do not establish clinically relevant urinary or bladder-tissue exposure. A translational study of an mTOR-directed candidate should first quantify the parent compound and relevant metabolites in timed urine, plasma, and paired bladder tissue. If adequate tissue exposure and baseline pathway activation are confirmed, a change in tissue *p*-S6 could serve as the primary pathway-proximal endpoint, with p21 as a mechanism-coherent secondary marker. Ki-67, TUNEL, cleaved caspase-3, and general oxidative-stress markers should be interpreted as supportive downstream outcomes rather than evidence of direct target engagement or clinical efficacy.

For an isoflavone hypothesis involving FGFR3/ERBB–PI3K signaling, an early presurgical study could enroll patients with FGFR3 mutation, fusion, or validated overexpression, because FGFR3/ERBB-related signaling and downstream MAPK–PI3K/AKT/mTOR pathways represent biologically relevant and targetable axes in BCa [173,181,182]. Parent isoflavones, S-equol, and major conjugated metabolites should be quantified in timed urine, plasma, and TURBT or cystectomy specimens. The primary pharmacodynamic endpoint should align with the proposed pathway and the baseline tumor state. In tumors with PI3K/mTOR activation, paired changes in *p*-AKT and *p*-S6 would be more informative than a broad panel of unrelated markers of apoptosis, inflammation, and immunity [181,183]. Where direct FGFR3 modulation is proposed, a receptor-proximal or MAPK-related endpoint should also be included. Progression should require concordance among urinary exposure, bladder-tissue concentration, molecular eligibility, and modulation of the prespecified pathway. Failure to detect the relevant chemical species in tumor tissue should preclude mechanistic interpretation of an unchanged pharmacodynamic biomarker.

Green tea catechins, particularly epigallocatechin-3-gallate (EGCG), are among the most extensively studied compounds in experimental BCa. Preclinical studies suggest that EGCG may influence proliferation, angiogenesis, invasion, autophagy, inflammatory signaling, and stem-like phenotypes [99,102]. EGCG has been reported to increase PTEN expression and reduce PI3K/AKT phosphorylation in BCa models [100]. It also inhibited IL-1β-induced uPAR expression and invasiveness in T24 cells by reducing NF-κB and AP-1 activity, along with changes in ERK1/2, JNK, and oxidative-stress-associated signaling [101]. Other experimental studies have described changes in LC3B-II, Beclin, caspase-3/9, BAX, and PI3K/AKT-regulated autophagy [103]. Reduced expression of CD44, CD133, ALDH1A1, and Nanog, and impaired tumor-sphere formation, have also been reported in association with modulation of the sonic hedgehog pathway [102]. These findings identify possible mechanisms but remain predominantly preclinical and require confirmation at urinary and urothelial exposures achievable in humans.

Curcumin is similarly well aligned with BCa biology but is relevant as much for its broad suppression of converging inflammatory and survival pathways as for a single dominant target. In bladder cancer models, curcumin inhibits the NF-κB, PI3K/AKT, JAK/STAT, MAPK, and WNT/β-catenin signaling pathways as well as reduces COX-2, VEGF, matrix metalloproteinases, and inflammatory cytokine levels that drive angiogenesis, epithelial–mesenchymal transition, and local invasion [84,116,119,122,125]. It may also suppress ERK1/2 signaling in benzidine-induced T24 proliferation models and induce apoptosis by modulating PI3K/Akt, along with effects on Bax, Bcl-2, caspases, and PARP cleavage [119,122]. Curcumin was reported to down-regulate IGF2 and to inhibit downstream IGF2-mediated PI3K/AKT/mTOR signaling, leading to decreased proliferation and migration of urothelial tumors [121]. Data from in vivo bladder carcinogenesis models also support antitumor and pro-apoptotic activity [120]. Its primary translational limitation is still pharmacokinetic rather than mechanistic. However, BCa may have a lower barrier to drug development than many solid tumors, as it allows direct assessment of urinary and urothelial exposure. Consequently, curcumin is best promoted through biomarker-rich, short-duration studies with predefined endpoints, such as *p*-AKT, *p*-S6, NF-κB transcriptional signatures, Ki-67, cleaved caspase-3/PARP, and EMT-related markers. These findings support network-level effects but remain dominated by in vitro studies using concentrations and parent compounds that may not reflect human urinary or urothelial exposure. Li et al. [43] similarly concluded that natural products in urothelial cancer engage multiple signaling pathways but remain limited by insufficient translational and clinical validation.

**Figure 5:**
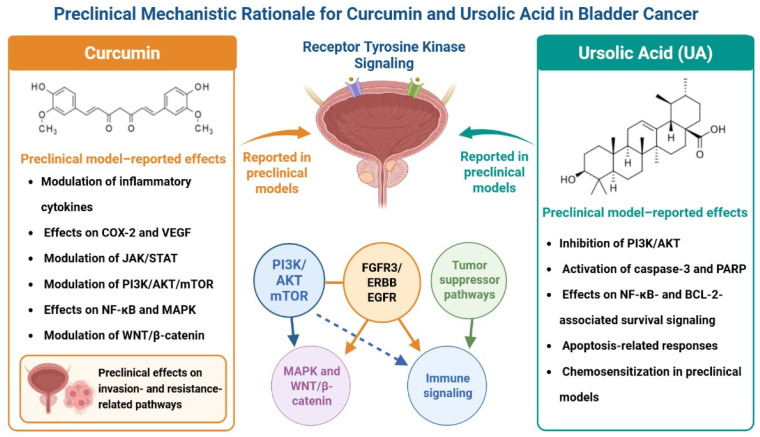
Possible signaling pathways influenced by curcumin and ursolic acid in preclinical bladder cancer models. Preclinical studies suggest that curcumin may influence NF-κB, PI3K/AKT/mTOR, MAPK, JAK/STAT, WNT/β-catenin, IGF2, inflammatory, angiogenic, and EMT-associated processes. Ursolic acid has been reported to affect PI3K/AKT, NF-κB/BCL-2, AMPK/JNK, caspase-3, and PARP-related signaling. Enhanced responses to gemcitabine have been described in experimental models but have not been clinically established. The illustrated relationships represent possible mechanisms and should not be interpreted as evidence of therapeutic efficacy or as support for unsupervised supplement use.

Ursolic acid (UA) deserves particular attention because it combines direct suppression of survival signaling with early evidence of chemosensitization. In T24 bladder cancer cells, UA reduces *p*-AKT and NF-κB activity, lowers BCL-2, and increases caspase-3 activation, consistent with apoptotic induction and growth restraint [113]. Additional studies show reduced cell viability, clonogenic survival, and migration, along with morphological features of cell death [118]. Experimental studies and network pharmacology analyses converge on PI3K/AKT downregulation, caspase-3 activation, and apoptosis induction, supporting a multi-node mechanism rather than single-target inhibition [113,115]. UA has also been shown to activate AMPK, stimulate JNK, and inhibit mTORC1 signaling, thereby contributing to growth suppression and downregulation of survival in T24 cells [124]. Importantly, UA enhances gemcitabine cytotoxicity in T24 and 5637 cell lines and xenografts, with combined PI3K/AKT suppression and JNK activation providing a mechanistic basis for chemotherapy sensitization [114]. These features make UA one of the more extensively investigated preclinical candidates in BCa, although hydrophobicity, poor solubility, and limited oral bioavailability remain substantial barriers that will likely require formulation-enabled development (Figure 5) [42,117,123]. The principal preclinical mechanisms proposed for curcumin and UA in BCa models are summarized in Figure 5.

Flavonols such as kaempferol, myricetin, and fisetin regulate cell-cycle progression, mitochondrial apoptosis, and EMT-related pathways in preclinical BCa models [105,106,107]. However, circulating flavonols in humans are predominantly present as glucuronidated and sulfated metabolites rather than as free aglycones. This limits the direct relevance of experiments using micromolar concentrations of unconjugated parent compounds [105,106,107]. Kaempferol has been associated with inhibition of cyclin–CDK signaling, reduced Rb phosphorylation, and modulation of BCL-2 family proteins, whereas myricetin has been reported to activate p53–p21/p27-dependent checkpoint pathways and regulate EMT-related markers across preclinical cancer models, including bladder cancer studies [106,107]. Although fisetin shares several of these mechanistic features, the current evidence supporting its activity is more extensive in prostate cancer than in BCa. Collectively, these observations suggest that flavonols in bladder cancer are best characterized as modulators of cell-cycle control, apoptosis, and invasion-related signaling, supporting their evaluation in biomarker-driven peri-procedural studies rather than in efficacy-focused clinical development at this stage. This cautious interpretation is further reinforced by a major translational challenge: circulating flavonols in humans are predominantly present as glucuronidated and sulfated metabolites rather than as free aglycones, raising concerns regarding the direct extrapolation of in vitro findings obtained with micromolar concentrations of parent compounds. Therefore, future clinical studies should incorporate exposure verification by quantifying both parent compounds and biologically relevant metabolites [104,111,112].

Isoflavones play a more targeted, yet still impactful, role in BCa. Its strongest mechanistic rationale appears to lie in molecularly defined FGFR3-driven and RTK-enriched subsets rather than in a universal urothelial effect. The preclinical evidence supports anti-proliferative activity and suppression of FGFR3-associated signaling pathways, whereas a short presurgical clinical trial of genistein demonstrated a measurable effect on tumor *p*-EGFR expression but little change in global markers of proliferation or apoptosis during the exposure period [110]. In particular, daidzein has been reported to block FGFR3-associated signaling in FGFR3-dependent BCa models, offering an immediate entry point into the RTK-to-PI3K/MAPK circuitry that may warrant the development of biomarker-enrichment strategies in defined molecular subsets [108,109,110]. Therefore, while isoflavones are not expected to have broad activity in BCa, if tissue exposure and pathway modulation can be demonstrated in humans, they could be beneficial in FGFR3-mutant or RTK-dominant disease.

Other polyphenols, such as resveratrol and quercetin, also map nicely onto BCa signaling topology despite having limited translational richness. Resveratrol reduces proliferative and invasive behavior via PI3K/AKT/mTOR inhibition, mitochondrial apoptosis, and modulation of non-coding RNA networks, while quercetin manages PI3K/AKT, MAPK, NF-κB, JAK/STAT, and WNT-associated programs directed towards cell-cycle arrest, apoptosis, and EMT attenuation [23,28,185,186]. As with other tumor types, their main limitation is not mechanistic plausibility but rather the lack of relevant human data, combined with low and variable bioavailability. As such, these agents are better progressed through formulation-facilitated early-phase studies focused on urinary/tissue exposure and target engagement than on outcome-primed intervention trials.

Carotenoids, such as lycopene, seem to act in a less direct, more limited way with respect to BCa progression than their currently accepted role of protecting against other cancers through direct antitumor cytotoxicity; instead, they appear to act indirectly by intercepting inflammation- and oxidative stress-linked molecules. This might still be relevant in urothelial neoplasia, as NF-κB-dependent inflammation, cytokine signaling, and oxidative stress pathways are implicated in progression/metastasis/recurrence [187,188,189]. Prospective biomarker studies conducted early in the field suggested an association between lower circulating carotenoid levels, including lycopene (HL 1948), and higher BCa risk, although not all were significant [190]. Case–control data have suggested similar protective associations for several carotenoids, especially among smokers [191]. In addition, stronger inverse associations have not been consistently observed in larger cohort studies, which are characterized by substantial heterogeneity and potential residual confounding [192,193]. Experimental evidence is similarly mixed: rodent models of bladder carcinogenesis show little independent chemopreventive activity for lycopene and β-carotene compared with other agents, but some modifications in proliferation [194].

BCa therefore offers a useful setting for precision exposure studies, not an abbreviated route to efficacy trials. Initial human investigations should quantify parent compounds and metabolites in urine and tissue, establish local and systemic safety, and evaluate predefined pharmacodynamic endpoints. Molecular subtype, FGFR3 and TP53/RB1 status, NRF2 activity, immune contexture, and urinary molecular profiles should be incorporated as stratification variables [195]. Recurrence-prevention, chemotherapy-sensitization, or clinical-response trials should not be prioritized until exposure-linked target engagement and pharmacological compatibility have been demonstrated.

### 4.3. Renal Cell Carcinoma

Renal cell carcinoma (RCC), particularly clear cell RCC (ccRCC), has a molecular architecture that differs from that of prostate and bladder cancers. The biology of ccRCC is largely dominated by loss of the von Hippel–Lindau (VHL) tumor suppressor, resulting in stabilization of hypoxia-inducible factors, particularly HIF-2α, with context-dependent involvement of HIF-1α. This activates hypoxia-responsive programs that promote VEGF-driven angiogenesis, metabolic reprogramming, tumor growth, and immune modulation [8,18,19]. PI3K/AKT/mTOR, c-MET, NF-κB, and other stress-response pathways further contribute to tumor survival and treatment resistance [4,18,138]. Hsieh et al. [196] accordingly characterized RCC as a heterogeneous group of malignancies shaped by distinct genomic, angiogenic, metabolic, and immune programs.

The relevance of a proposed nutraceutical mechanism depends strongly on RCC subtype. Papillary RCC includes MET-driven tumors as well as subgroups characterized by alterations in cell-cycle regulation, chromatin remodeling, Hippo signaling, NRF2-associated stress responses, and cellular metabolism [197]. Chromophobe RCC, by contrast, displays extensive chromosomal and mitochondrial abnormalities and differs markedly from VHL-deficient ccRCC. Contemporary metabolic analyses further indicate that clear-cell, papillary, and chromophobe tumors exhibit distinct dependencies involving oxygen sensing, nutrient utilization, lipid and amino acid metabolism, and tricarboxylic acid cycle activity [198]. Proteogenomic analyses have also identified aggressive ccRCC subsets characterized by coordinated metabolic disruption, immune activation, and metastatic behavior [199]. Consequently, effects on HIF or VEGF signaling may be most relevant to ccRCC, whereas MET-directed hypotheses may apply only to selected papillary tumors and should not be generalized across RCC histologies.

Target-compartment assessment in RCC should not rely solely on urinary concentration. Renal excretion may produce high urinary levels of a compound or metabolite without demonstrating exposure within the tumor parenchyma, while systemic exposure may be more relevant for vascularized renal tumors and metastatic disease. In a kidney tumor xenograft metabolomics study, Ganti et al. [200] found that serum metabolic changes more closely reflected tumor tissue alterations than did urine, suggesting that the most accessible biofluid is not necessarily the best surrogate for renal tumor exposure. Conversely, paired human ccRCC analyses by Nizioł et al. [201] identified selected metabolites that were increased in both tumor tissue and patient urine, suggesting that some tumor-derived or tumor-enriched species can enter the urinary space. However, the correspondence was metabolite-specific rather than universal. Nutraceutical studies in RCC should therefore use paired plasma or serum, urine, tumor tissue, and adjacent kidney measurements whenever feasible and should distinguish renal filtration or excretion from tumor uptake. Parent compounds and active metabolites should be quantified separately, and bulk-tissue measurements should be interpreted cautiously because intratumoral vascular, necrotic, immune, and metabolic regions may differ in exposure.

Several polyphenols show plausible mechanistic overlap with RCC biology. Across cancer models, polyphenols such as curcumin, resveratrol, quercetin, and EGCG have been reported to modulate PI3K/AKT/mTOR signaling, NF-κB activation, MAPK-related cascades, and angiogenesis-associated outputs [23,125,186]. Curcumin is mechanistically relevant because it suppresses growth factor-linked PI3K/AKT/mTOR signaling and modulates inflammatory and pro-apoptotic stress responses, suggesting potential overlap with inflammatory and metabolic circuits involved in RCC progression [125,135]. Quercetin and resveratrol also influence receptor-to-kinase signaling cascades, including PI3K/AKT and MAPK pathways, thereby attenuating proliferative and survival-related outputs that intersect with hypoxia-driven cancer biology [23,186]. Among these compounds, quercetin and resveratrol appear particularly relevant because experimental evidence links them more directly to RCC-associated molecular processes. Quercetin, especially in combination with β-hydroxybutyrate, has been shown to suppress hypoxia-induced angiogenic signaling and multidrug resistance programs in Caki-1 ccRCC cells, including downregulation of HIF-1α, HIF-2α, VEGF, Ang-1, and MDR4 expression [131]. Resveratrol similarly exhibits antitumor activity in RCC models through apoptosis induction, cell-cycle arrest, and modulation of angiogenesis-related signaling. Notably, resveratrol has been reported to sensitize renal cancer cells to the VEGFR inhibitor tivozanib by enhancing apoptosis, inhibiting migration, and reducing HIF1α and VEGFC expression [133]. In addition, resveratrol nanoparticle formulations suppress RCC cell migration and invasion by reducing ERK activation and MMP-2 activity, indicating that optimized delivery systems may be necessary to further explore its biological and translational potential in renal tumors [134]. These findings support mechanistic plausibility but remain predominantly confined to experimental models, mainly ccRCC-related. As also noted by Li et al. [43], natural products have been associated with numerous pathway-level effects in renal cancer, but subtype-specific validation, achievable tissue exposure, and human pharmacodynamic confirmation remain limited.

Carotenoids may play a role in the biology of RCC interception. In a TSC2 mutant Eker rat model that develops renal tumors through mTOR activation, dietary lycopene significantly decreased both tumor multiplicity and tumor size, thus also demonstrating its potential to modulate renal tumorigenesis [202]. Epidemiologic studies generally support this possibility. A higher dietary intake of lycopene was associated with a reduced RCC risk in the Women’s Health Initiative cohort [127], and case–control studies show inverse associations between carotenoid intake, including that of β-carotene and lutein/zeaxanthin, and RCC risk [128]. While these findings do not prove causality, they imply that dietary carotenoids may be relevant to long-term prevention.

Despite these areas of mechanistic overlap, RCC is one of the most pharmacologically challenging settings for nutraceutical research in uro-oncology. Advanced disease is commonly treated with VEGFR-directed tyrosine kinase inhibitors, immune checkpoint inhibitors, mTOR-directed agents, or combination regimens. Ciccarese et al. [203] highlighted the clinical importance of VEGFR-TKI and immune checkpoint inhibitor combinations, while Coffey & Simon [198] emphasized the close relationship among metabolic reprogramming, immune suppression, and treatment resistance. Tsai et al. [204] similarly situated the potential effects of natural products within the interconnected biology of the VHL–HIF axis, metabolic plasticity, redox regulation, angiogenesis, and tumor–immune interactions. Although these observations support further mechanistic investigation, they also underscore the difficulty of distinguishing a true tumor-directed effect from altered drug exposure, treatment antagonism, overlapping toxicity, or a nonspecific systemic response.

Nutraceuticals that affect CYP enzymes, P-glycoprotein or other transporters, angiogenic signaling, immune activation, redox balance, hepatic metabolism, or renal function may modify the exposure, toxicity, or activity of standard RCC therapies [8,17,19,132]. Although these products are generally perceived as low-toxicity, concentrated nutraceutical preparations may produce clinically relevant CYP3A4- or P-gp-mediated interactions. Given the limited clinical validation, variable pharmacokinetics, and potential effects on anticancer drug exposure, nutraceutical development for RCC should follow an interaction-first and safety-first strategy [204,205]. Initial studies should characterize preparation composition, systemic exposure, active metabolites, hepatic metabolism, renal elimination, transporter effects, and potential interactions with VEGFR-, mTOR-, and immune-directed regimens.

Subsequent pharmacodynamic evaluation should be matched to both histology and treatment context. Appropriate endpoints may include VHL–HIF and VEGF-related activity in ccRCC, MET-associated signaling in selected papillary tumors, and subtype-specific metabolic, redox, angiogenic, or immune markers. The phase I study combining isoquercetin with sunitinib demonstrates that a flavonoid derivative can be investigated alongside targeted therapy when interaction risks are explicitly monitored [126,136,139]. However, this study establishes feasibility under controlled conditions rather than adjunctive efficacy or a class-wide benefit. Efficacy-oriented combinations should therefore not proceed until clinically meaningful pharmacokinetic and pharmacodynamic interference has been excluded and acceptable renal, hepatic, and treatment-specific safety has been demonstrated.

Efficacy-focused investigations of nutraceuticals in RCC should be initiated only after the formulation’s quality and standardization, human bioavailability, absorption and metabolism, safety and tolerability, systemic exposure and organ-specific disposition, and compatibility with established therapeutic regimens have been thoroughly characterized. This requirement is particularly important in patients receiving VEGFR-directed tyrosine kinase inhibitors, mTOR inhibitors, or immune checkpoint inhibitors, because concentrated nutraceutical preparations may influence metabolic enzymes, transporters, organ function, immune activity, or treatment-related toxicity [18,19,132,151,204].

Tumor-related biological-response markers should be introduced only after adequate exposure, acceptable tolerability, and the absence of clinically meaningful treatment interactions have been demonstrated. A subsequent study should be restricted to a single RCC histology, a clearly defined treatment background, and one formulation-specific mechanistic hypothesis. For example, a candidate proposed to influence pseudohypoxic signaling in clear-cell RCC could be assessed using one prespecified VHL–HIF–VEGF-related marker, whereas a candidate proposed to influence mTOR signaling could be evaluated using *p*-S6 in tumors with baseline pathway activation. Multiple pathway markers should not be combined routinely unless the tumor context and proposed mechanism justify their inclusion. Progression to efficacy-oriented research should require consistent product quality, predictable human exposure, an acceptable safety and tolerability profile, absence of clinically important nutraceutical–drug interactions, and evidence that the parent compound or relevant metabolites reach the intended biological compartment. The RCC evidence base remains predominantly preclinical, and reported effects on HIF–VEGF, PI3K/AKT/mTOR, NF-κB, STAT, MAPK, apoptosis, and metabolic signaling support mechanistic plausibility rather than clinical applicability [43,206,207,208]. At present, the most appropriate priorities are product standardization, bioavailability, renal disposition, safety, tolerability, and treatment-interaction assessment rather than adjunctive efficacy testing. Claims of clinical applicability should therefore be avoided unless supported by formulation-specific human exposure, renal or tumor-tissue distribution, treatment-interaction assessment, exposure-linked biological activity, and controlled human evidence.

### 4.4. Testicular Germ Cell Tumors

Testicular germ cell tumors (TGCTs) differ from the other uro-oncology malignancies because of their developmental origin, young age distribution, exceptional chemosensitivity, and high curability. These features create a particularly low tolerance for uncertainty regarding adjunctive compounds. The primary objective of any nutraceutical investigation must be to preserve the efficacy and safety of cisplatin-based curative treatment [21,22]. Chovanec et al. [209] emphasized that the success of cisplatin-based therapy has produced a growing population of long-term germ cell tumor survivors, while also generating persistent renal, neurological, auditory, cardiovascular, and reproductive toxicities. This clinical context provides a rationale for supportive interventions but imposes a particularly high evidentiary threshold for demonstrating that such interventions do not compromise cure. TGCTs are also molecularly and histologically heterogeneous. Seminoma, embryonal carcinoma, yolk sac tumor, choriocarcinoma, teratoma, and mixed tumors represent distinct developmental, epigenetic, transcriptional, and immune states. Integrated molecular analyses by Shen et al. [210] showed that KIT, KRAS, and NRAS alterations occur more frequently in tumors containing seminoma components, whereas nonseminomatous tumors exhibit different differentiation and epigenetic programs. Findings obtained from a single cell line or histological subtype should therefore not be generalized across the full TGCT spectrum. Li et al. [43] similarly identified only limited preclinical observations for natural products in testicular cancer, with few disease-specific studies and little animal or human pharmacological validation.

Exposure characterization in TGCT should include both the nutraceutical-derived chemical species and the pharmacology of cisplatin. Plasma concentrations of parent compounds and metabolites should be related to those used in TGCT models, while parallel measurements should determine whether the intervention alters cisplatin exposure, renal elimination, platinum–DNA adduct formation, or tissue distribution. A metabolite that is inactive against germ cell tumors may nevertheless alter cisplatin transport, metabolism, redox responses, or normal-tissue toxicity. Conversely, a cytoprotective metabolite may reach both normal and malignant testicular compartments. Non-interference studies should therefore be based on measured systemic and tissue exposure rather than administered dose alone.

Although PI3K/AKT/mTOR, MAPK, NF-κB, oxidative stress, DNA damage response, and apoptosis contribute to germ cell tumor biology, there is no validated nutraceutical-guided biomarker framework for TGCT [21,22,43,211]. Most proposed compounds, including EGCG, quercetin, resveratrol, and ursolic acid, have attracted attention because of their multitarget effects on proliferation, apoptosis, oxidative stress, and inflammatory signaling in other tumor systems [42,140,186]. However, in TGCT, these compounds currently lack a clearly established tumor-specific mechanistic anchor, and the available evidence remains insufficient to define exposure thresholds, TGCT-context specificity, or pharmacodynamic biomarkers indicative of meaningful pathway engagement [21,22]. A similar limitation applies to carotenoids such as lycopene, whose proposed roles in oxidative stress modulation and inflammatory control remain biologically plausible but insufficiently linked to clinically actionable vulnerabilities in TGCT [81,212]. Isoflavones further illustrate the challenge of cross-context translation: although their relevance is mechanistically more coherent in prostate cancer because of their reported interactions with AR signaling and PI3K/AKT/mTOR- or MAPK/ERK-linked pathways, their relevance in TGCT remains largely theoretical in the absence of a comparable endocrine or lineage-defined axis [66,211]. The principal translational constraint in TGCT is therefore twofold: an underdeveloped, tumor-specific evidence base and an exceptionally narrow therapeutic risk tolerance. In a disease where a cure is frequently achievable, any adjunctive intervention must demonstrate unequivocally that it does not compromise chemotherapy efficacy, alter pharmacokinetics, or exacerbate toxicity. Future research should therefore compare nutraceutical effects across molecularly and histologically characterized TGCT models and should evaluate cisplatin pharmacokinetics, DNA damage signaling, apoptosis, tumor-cell killing, renal and neurological toxicity, and reproductive outcomes. Subtype-specific hypotheses remain secondary to demonstrating non-interference with curative therapy. Until such evidence is available, clinical adjunctive nutraceutical studies during cisplatin treatment are not justified.

In TGCT, the primary trial logic should prioritize non-interference over pathway-response screening. A candidate proposed to reduce cisplatin toxicity should first be evaluated in cisplatin-sensitive and resistant TGCT models using measured nutraceutical exposure together with cisplatin pharmacokinetics, platinum–DNA adduct formation, DNA-damage signaling, and tumor-cell killing. Renal, neurological, or reproductive protection should be evaluated in parallel but should not be considered actionable unless antitumor activity is preserved. Cleaved caspase-3 or cleaved PARP may therefore be useful as tumor non-interference endpoints in this context, rather than as general evidence of nutraceutical anticancer activity.

The principal translational question is therefore not whether a nutraceutical shows anticancer activity in an isolated model, but whether it can be administered without reducing cisplatin exposure, platinum–DNA adduct formation, DNA-damage signaling, apoptosis, or tumor-cell killing. Cocetta et al. [213] reviewed interactions between nutraceutical supplementation and platinum-based chemotherapy and highlighted both potential supportive effects and the possibility of altered drug metabolism or therapeutic activity. Dasari et al. [214] similarly showed that combinations of cisplatin with natural products may produce synergistic, additive, neutral, or antagonistic effects depending on the compound, dose, tumor model, and treatment schedule. These variable findings preclude assumptions of compatibility based solely on antioxidant, anti-inflammatory, or cytoprotective properties. Much of the available literature focuses on mitigating cisplatin-associated toxicity rather than on direct TGCT treatment. Rahimi et al. [215] summarized predominantly preclinical evidence concerning natural products and cisplatin-induced male reproductive injury, whereas Abdel-All et al. [216] evaluated a nutraceutical formulation for protection against cisplatin-induced testicular toxicity in rats. Such studies support hypotheses related to fertility preservation and toxicity reduction but do not establish selective protection of normal tissues. A compound that attenuates oxidative stress, DNA damage, or apoptosis in renal, neural, or testicular tissues could theoretically protect malignant germ cells through related mechanisms. Future preclinical studies should therefore evaluate tumor and normal-tissue responses concurrently. Experimental designs should include molecularly and histologically characterized TGCT models, cisplatin-sensitive and cisplatin-resistant systems, pharmacokinetic interaction analyses, measurements of platinum–DNA adducts or other DNA-damage markers, tumor-cell apoptosis and viability, and relevant renal, neurological, and reproductive toxicity endpoints. Demonstration of antitumor non-interference should precede investigation of nephroprotection, neuroprotection, fertility preservation, spermatogenic recovery, or long-term survivorship outcomes. TGCTs should consequently be regarded as a non-interference-led rather than an efficacy-led setting for nutraceutical development. Given the high curability of the disease and the narrow tolerance for treatment disruption, clinical adjunctive studies during cisplatin therapy should not proceed until pharmacokinetic compatibility, preservation of tumor cytotoxicity, and selective normal-tissue protection have been established. The translational threshold should be especially high in TGCT because cisplatin-based therapy is frequently curative. Although natural products have reduced cisplatin-associated testicular injury in animal models, such findings do not establish clinical benefit or antitumor non-interference [215,217,218]. Research should therefore prioritize preservation of cisplatin exposure, platinum–DNA adduct formation, DNA-damage signaling, apoptosis, and tumor-cell killing before evaluating supportive or independent nutraceutical effects. Until these requirements are met, nutraceutical strategies for TGCT should be considered preclinical and investigational rather than therapeutically applicable.

### 4.5. Mechanistic Plausibility Versus Clinical Applicability in RCC and TGCT

In renal cell carcinoma and testicular germ cell tumors, mechanistic plausibility currently exceeds clinical applicability. Natural products have been reported to influence several cancer-relevant pathways in both malignancies, but the evidence is mainly derived from cell culture and animal studies, with limited formulation-specific human exposure data and little controlled intervention evidence. Pathway modulation in experimental models should therefore not be presented as a therapeutic opportunity. The immediate priorities are product standardization, clinically relevant exposure, safety, treatment compatibility, and carefully selected biological-response endpoints.

In RCC, natural products have shown preclinical effects on apoptosis, angiogenesis, metastatic behavior, metabolic adaptation, treatment sensitivity, and immune signaling through pathways including VHL–HIF–VEGF, PI3K/AKT/mTOR, NF-κB, STAT, and MAPK [43,206,208,219]. These findings provide a biological rationale for further investigation but do not establish clinical activity. Advanced RCC treatment remains centered on VEGFR-directed tyrosine kinase inhibitors, mTOR-targeted agents, and immune checkpoint-based regimens, all of which introduce substantial interaction and toxicity considerations [206,208]. Patients may also have impaired renal function, cumulative treatment toxicity, and limited tolerance for additional pharmacologically active agents. Therefore, natural products in RCC should currently be described as mechanistically interesting candidates rather than clinically actionable adjuncts. Experimental observations, including apparent sensitization to targeted agents or enhanced apoptosis in combination models, remain hypothesis-generating until supported by chemically defined formulations, human pharmacokinetics, characterization of active metabolites, renal and tumor tissue distribution, treatment-specific interaction testing, and controlled clinical data [206,220]. Improved delivery systems may address poor bioavailability, but formulation enhancement alone does not demonstrate tumor exposure, target engagement, or therapeutic benefit.

An even more conservative framework is required for TGCT because cisplatin-based treatment is highly effective and frequently curative. In this setting, the primary translational objective should not be exploratory nutraceutical modulation of tumor pathways, but demonstration of safety and non-interference with established curative therapy. Any proposed supportive intervention must be shown not to reduce cisplatin exposure, alter platinum–DNA adduct formation, attenuate tumor DNA-damage signaling, protect malignant germ cells from apoptosis, or otherwise compromise tumor-cell killing.

Several natural products, including bilobetin and naringenin, have reduced oxidative stress, inflammation, apoptosis, and testicular injury in preclinical models of cisplatin toxicity [215,217,218]. However, these findings are largely based on short-term animal studies and do not establish clinical protection, long-term preservation of spermatogenesis, or the absence of interference with antitumor efficacy. More broadly, natural product–platinum combinations may produce beneficial, neutral, or detrimental effects depending on the identity of the natural product, formulation, dose, exposure, and tumor context [213,214]. A pomegranate nanoparticle formulation that reduced cisplatin-associated testicular toxicity while preserving antitumor activity in a mouse model illustrates the type of dual normal-tissue and tumor assessment required before supportive development can be considered [221]. Nevertheless, such findings remain preclinical and cannot be generalized to patients with TGCT without confirmation of formulation-specific exposure, cisplatin pharmacokinetics, tumor non-interference, reproductive safety, and long-term clinical outcomes. Accordingly, RCC should be characterized as a setting of mechanistic promise but premature clinical translation, whereas TGCT should be approached through a safety- and non-interference-first framework [43,206,208]. Neither malignancy currently has sufficient human evidence to support the use of nutraceuticals as therapeutic adjuncts. In RCC, the next justified steps are formulation-specific pharmacokinetic, tissue-distribution, interaction, and safety studies. In TGCT, adjunctive investigation should not proceed beyond preclinical development until preservation of cisplatin pharmacology and antitumor activity has been convincingly demonstrated.

### 4.6. Nutraceutical Modulation of Antitumor Immunity and Potential Interactions with Immune Checkpoint Inhibitors

Immune checkpoint inhibitors (ICIs) have substantially changed the treatment landscape of several solid tumors, including uro-oncology malignancies. Their efficacy, however, depends not only on tumor-intrinsic checkpoint expression but also on the composition and functional state of the tumor microenvironment (TME). Regulatory T cells, myeloid-derived suppressor cells, tumor-associated macrophages, dysfunctional dendritic cells, hypoxia, adenosine signaling, tryptophan depletion, and metabolic competition can collectively restrict T-cell infiltration and effector function, thereby contributing to primary or acquired ICI resistance [222,223,224,225].

Nutraceuticals may intersect with ICI therapy because their effects extend beyond direct tumor-cell signaling. Selected dietary compounds, microbial products, and metabolic interventions can influence dendritic-cell maturation, antigen presentation, CD8^+^ T-cell activation, regulatory T-cell differentiation, macrophage polarization, cytokine production, and gut microbial metabolism. These properties create a potential opportunity to convert an immunosuppressive TME into a more immunologically responsive state. However, immunomodulation is inherently context-dependent, and the same compound may enhance one immune compartment while suppressing another. Nutraceutical–ICI combinations should therefore not be assumed to be beneficial solely because a compound has anti-inflammatory or antioxidant properties [141,142,226]. Several preclinical findings illustrate potential immune-enhancing mechanisms. Vitamin E has been reported to inhibit the intracellular checkpoint SHP1 in dendritic cells, thereby improving antigen cross-presentation, antigen-specific T-cell responses, and enhancing ICI activity in experimental models; retrospective clinical observations also suggested improved outcomes among selected ICI-treated patients who reported vitamin E use [227]. A defined exopolysaccharide derived from Lactobacillus delbrueckii, EPS-R1, increased CCR6^+^ CD8^+^ T cells and improved immune-checkpoint blockade in tumor-bearing mice [228]. Fucoidan similarly enhanced experimental anti-PD-1 activity by increasing T-cell receptor/CD3 and JAK–STAT signaling and enhancing CD8^+^ T-cell activation [229]. Arginine supplementation has also been shown preclinically to increase tumor-infiltrating CD8^+^ T cells, promote M1-like macrophage polarization, and enhance antitumor immune activity [230]. The microbiota represents another important interface between nutrition and ICI efficacy. Higher dietary fiber intake has been associated with improved ICI response and progression-free survival, whereas use of commercial probiotics has been associated with less favorable outcomes in an observational melanoma cohort. In corresponding experimental models, low dietary fiber and selected probiotic interventions impaired anti-PD-1 activity [231]. These findings demonstrate that a defined microbial product with a specific immune mechanism cannot be considered equivalent to unsupervised commercial probiotic supplementation. Dietary fiber, short-chain fatty acid production, microbial diversity, antibiotic exposure, and the composition of both intestinal and intratumoral microbial communities may each modify ICI responsiveness [232,233]. These observations are potentially relevant to uro-oncology but should not be extrapolated directly from melanoma or non-urologic tumor models. In RCC and urothelial carcinoma, where immune-based therapies have an important clinical role, nutraceuticals could theoretically alter antigen presentation, CD8^+^ T-cell infiltration, macrophage polarization, angiogenic–immune coupling, or myeloid suppression. Such effects might enhance ICI activity, but they could also reduce therapeutic efficacy by promoting regulatory T-cell differentiation, suppressing effector T-cell activation, or interfering with inflammatory signals required for antitumor immunity. Plant-derived immunomodulators have demonstrated both immune-stimulatory and immune-suppressive properties, including compound-specific suppression of T-cell activation and promotion of regulatory T-cell phenotypes [141].

PCa provides a different immunological context because most prostate tumors exhibit limited T-cell infiltration and relative resistance to immune-checkpoint blockade. A preclinical study demonstrated that an optimized cyclic ketogenic diet and ketone supplementation altered the epigenetic and immune landscape of PCa, increased antitumor immune activity, and improved ICI responsiveness [234]. This provides a proof-of-concept that metabolic interventions may modify an immunologically “cold” prostate TME. Nevertheless, these findings remain preclinical and should not be interpreted as support for uncontrolled ketogenic interventions in patients receiving anticancer treatment.

Potential risks must be considered as carefully as potential benefits. Excessive suppression of inflammatory signaling could reduce dendritic-cell activation, T-cell priming, or effector responses required for ICI activity. Conversely, strong immune stimulation could theoretically increase immune-related adverse events. Antioxidant and redox-modulating compounds may also affect tumor-cell stress responses, immunogenic cell death, and immune-cell metabolism in dose-, timing-, and tissue-dependent ways. Moreover, concentrated extracts and formulation-enhanced products may alter CYP enzymes and transporters, concomitant drug exposure, and systemic toxicity. Dietary exposure, purified compounds, commercial supplements, probiotics, and pharmacologically optimized formulations should therefore be treated as distinct interventions. Future studies in uro-oncology should evaluate nutraceutical–ICI interactions using biomarker-rich preclinical and early-phase clinical designs rather than focusing on the clinical investigation of potential supportive effects. Relevant endpoints should include systemic and tumor exposure, dendritic-cell activation, CD8^+^ T-cell infiltration, CD8^+^/Treg ratios, myeloid-derived suppressor cells, macrophage phenotypes, T-cell receptor clonality, cytokine profiles, microbial composition and metabolites, tumor PD-L1 expression, and immune-related adverse events. Baseline diet, antibiotic and probiotic exposure, corticosteroid use, tumor molecular subtype, immune phenotype, and microbiome characteristics should also be incorporated into patient stratification. Accordingly, nutraceuticals should not currently be recommended as routine adjuncts to ICI therapy. The available evidence supports a dual, context-dependent model in which selected interventions may enhance antigen presentation or antitumor immunity, whereas others may induce immunosuppression, antagonize ICI activity, or increase toxicity. The immediate research priority is to identify which precisely defined compounds, formulations, dietary patterns, or microbial products are immunologically compatible with ICI therapy and in which molecular and immune contexts they may provide benefit or cause harm.

### 4.7. Integrated Perspective Across Uro-Oncology Malignancies

The available literature commonly describes natural products and nutraceutical-derived compounds as potential modulators of interconnected oncogenic networks rather than inhibitors of single molecular targets. In renal, urothelial, and testicular cancer models, natural products have been reported to influence cell growth, apoptosis, metastasis, therapy response, and the immune microenvironment through pathways including PI3K/AKT/mTOR, NF-κB, STAT, and MAPK [43]. Similarly, oleocanthal illustrates how a single nutraceutical-derived polyphenol may exert pleiotropic effects on inflammatory, metabolic, angiogenic, EMT-related, and drug-resistance-associated pathways, while also raising translational questions regarding pharmacokinetics and interactions [235]. However, the simultaneous alteration of multiple pathways should not be automatically interpreted as meaningful network control. Multitarget effects may produce synergy, but they may also induce compensatory signaling, pharmacological antagonism, altered treatment responses, or toxicity. Their translational significance therefore depends on achievable exposure, target engagement, tumor context, and compatibility with standard therapy.

The reciprocal relationship between NRF2 and NF-κB illustrates this context dependence. NF-κB/p65 may suppress NRF2-dependent transcription by competing for the CREB-binding protein and recruiting histone deacetylase 3 to MafK, whereas NRF2 can inhibit pro-inflammatory cytokine transcription under certain conditions [167,168]. Transient NRF2 activation may enhance detoxification and protect non-malignant tissues from oxidative or inflammatory injury. In established tumors, however, sustained NRF2 activity may promote reactive oxygen species detoxification, metabolic adaptation, proliferation, and treatment resistance [169,170]. Increased NRF2 signaling has also been experimentally linked to cisplatin resistance in bladder cancer, with NRF2 depletion partially restoring cisplatin sensitivity in resistant cells [184]. Consequently, an effect described broadly as antioxidant cannot be interpreted without considering the cellular compartment, tumor stage, treatment setting, dose, and duration of pathway activation.

Tumor subtype provides an additional level of biological specificity. AR-directed hypotheses may be relevant to luminal and AR-dependent prostate cancer but less applicable to basal-like, lineage-plastic, or neuroendocrine disease [151,152,153]. Similarly, FGFR3-associated mechanisms may be most relevant to selected luminal-papillary bladder tumors, whereas TP53/RB1-altered basal or neuroendocrine-spectrum tumors may depend more strongly on inflammatory, stromal, and lineage-plasticity programs [177,178,179]. VHL–HIF–VEGF-directed hypotheses are primarily applicable to clear-cell renal cell carcinoma and should not be generalized to papillary or chromophobe tumors, which possess distinct genomic, mitochondrial, and metabolic characteristics [197,198,210]. Testicular germ cell tumor histologies likewise differ in developmental state, molecular profile, and treatment biology [210]. These subtype distinctions should guide experimentally testable hypotheses rather than be interpreted as evidence of clinical readiness.

Meaningful translation will therefore require molecularly characterized models, chemically standardized preparations, quantitative measurement of parent compounds and active metabolites, and disease- and subtype-matched pharmacodynamic endpoints. Molecular or metabolic stratification should be linked to achievable systemic, tissue, or urinary exposure; target-proximal and circuit-level engagement; safety; microbiome-dependent biotransformation where relevant; and compatibility with standard treatment. Precision-nutrition and molecular-biomarker frameworks may facilitate such studies, particularly in bladder cancer, but require validation using exposure-linked mechanistic endpoints rather than general changes in inflammation, oxidative stress, proliferation, or apoptosis [195,236]. Potential interactions with immune checkpoint therapy further demonstrate that nutraceutical pleiotropy cannot be assumed to be beneficial. Compounds that suppress inflammatory or oxidative signaling may reduce injury in non-malignant tissues but could also impair antigen presentation, dendritic cell activation, T-cell priming, immunogenic tumor cell death, or immune checkpoint inhibitor activity. Conversely, selected dietary, microbial, or metabolic interventions may improve dendritic-cell function, CD8^+^ T-cell infiltration, and antitumor immune responsiveness. These effects are likely to depend on the intervention, dose, formulation, tumor type, treatment regimen, microbiome, and host metabolic state and therefore require direct pharmacological and immunological evaluation.

Across uro-oncology malignancies, the principal barriers remain poor or variable bioavailability, heterogeneous formulations, incomplete characterization of active metabolites, limited target-tissue exposure data, insufficient pharmacodynamic validation, and potential nutraceutical–drug interactions [235,237,238]. Advanced delivery systems may improve exposure but require independent evaluation of pharmacokinetics, tissue distribution, safety, and interaction risk. Similarly, describing a compound as a potential treatment sensitizer does not justify combination use without formal demonstration that it does not alter anticancer-drug exposure, therapeutic activity, or toxicity. Most nutraceuticals should therefore remain classified as investigational nutraceutical exposures rather than evidence-based oncologic adjuncts. The four malignancies also differ substantially in translational readiness. Prostate cancer currently provides the clearest mechanism-based setting because AR-centered biology and active surveillance or presurgical windows permit direct evaluation of prostate exposure and pathway-specific responses. This rationale is supported by the AR-focused analyses of Fontana et al. [26], Tyagi et al. [146], Wu et al. [144], and Pratama et al. [145]. Bladder cancer also offers a comparatively tractable framework because FGFR/RTK-defined molecular subsets, repeated urine sampling, and pre-TURBT or peri-cystectomy tissue access facilitate exposure-linked biomarker studies, consistent with the precision approaches described by Xiao et al. [173], Zhang et al. [176], Li et al. [109], and Meng et al. [174]. These characteristics support mechanism- and biomarker-based investigation but do not establish nutraceutical clinical benefit.

These pharmacokinetic limitations should be incorporated directly into mechanistic interpretation rather than presented as a separate general barrier. For a pathway claim to be translationally meaningful, the responsible chemical species must be identified and shown to reach the appropriate compartment at an exposure compatible with the reported effect. In PCa, this requires measuring prostate tissue and recognizing that conjugated or microbial metabolites may predominate over the parent compound. In BCa, timed urinary exposure provides a useful but incomplete measure that should be linked to bladder-mucosal or tumor uptake. In RCC, urine cannot be assumed to represent tumor exposure, requiring paired systemic, urinary, and tissue assessment. In TGCT, systemic nutraceutical exposure must be interpreted together with cisplatin pharmacokinetics and tumor non-interference. Thus, the relevant translational unit is not the compound name alone, but the defined preparation, chemical species, concentration, compartment, tumor context, and treatment setting.

Renal cell carcinoma represents a more conditional setting because its biology and treatment are shaped by angiogenic signaling, metabolic adaptation, immune regulation, renal physiology, and combination systemic therapy [196,203,204,239]. Nutraceutical development in RCC should therefore begin with formulation characterization, pharmacokinetics, renal and hepatic disposition, treatment-interaction testing, and safety assessment. Testicular germ cell tumors impose the highest evidentiary threshold because cisplatin-based therapy is frequently curative. Preservation of cisplatin pharmacokinetics, DNA-damage signaling, and tumor-cell killing must take precedence over exploratory anticancer or supportive effects, as emphasized by Chovanec et al. [209] and the platinum-interaction analyses of Dasari et al. [214] and Cocetta et al. [213]. Accordingly, nutraceutical development should follow a disease-ranked strategy: mechanism- and biomarker-led window studies in prostate and bladder cancer, interaction- and disposition-led investigation in renal cell carcinoma, and non-interference-led preclinical evaluation in testicular germ cell tumors. This hierarchy reflects differences in translational opportunity, treatment complexity, and clinical risk rather than the number of molecular pathways a compound reportedly affects.

### 4.8. Cross-Study Interpretation and Key Evidence Gaps

Across the studies discussed above, apparent inconsistencies should not be interpreted solely as evidence for or against the biological activity of a given nutraceutical. Differences in formulation, purity, dose, treatment duration, route of administration, experimental model, disease stage, baseline diet, concomitant therapy, and analytical methods may substantially influence the reported outcomes. In particular, many positive preclinical findings were generated using concentrations of unconjugated parent compounds that may not be achievable in humans, whereas negative clinical studies often lacked tissue-exposure measurements or pharmacodynamic confirmation. Consequently, it is frequently unclear whether a null clinical result reflects true biological inactivity, inadequate exposure, inappropriate patient selection, or use of an insensitive endpoint. The literature is also affected by selective emphasis on positive mechanistic findings, limited replication across independent models, and substantial heterogeneity in the preparations described under the same compound name. Human studies are generally small, short-term, and rarely stratified by tumor molecular subtype, metabolic phenotype, microbiome-related biotransformations, or background treatment. These limitations restrict causal interpretation and make direct comparison across studies difficult. Future investigations should therefore be designed to distinguish among competing explanations for inconsistent findings. Such studies should use standardized formulations, quantify parent compounds and active metabolites, confirm target-tissue exposure, predefine pathway-specific pharmacodynamic endpoints, and select participants according to biologically justified molecular or metabolic characteristics. This approach would allow the field to move beyond sequential descriptions of positive and negative studies toward explicit testing of why, in whom, and under what pharmacological conditions a nutraceutical may or may not produce a measurable effect.

## 5. Safety, Bioavailability, and Human Evidence as Prerequisites for Nutraceutical Development

Before nutraceuticals are evaluated in uro-oncology, their quality, standardization, bioavailability, absorption and metabolism, dose–response relationships, safety, tolerability, and compatibility with established anticancer treatment must be adequately characterized. These requirements are particularly important because patients with prostate, bladder, or renal cancers frequently receive complex multidrug regimens, including androgen receptor pathway inhibitors, taxane-based chemotherapy, VEGFR-directed tyrosine kinase inhibitors such as pazopanib and axitinib, agents targeting c-MET- or FGFR-related pathways, and immune checkpoint inhibitors. These treatments may be administered sequentially or concurrently, often in patients with comorbidities, altered nutritional status, and substantial background medication use. Consequently, the introduction of a concentrated dietary bioactive into this clinical setting requires careful consideration of metabolic pathways, transporter activity, immune regulation, organ function, and treatment-related toxicity [4,19,132]. Many nutraceuticals have been reported to influence molecular networks that are also affected by approved anticancer therapies, including PI3K/AKT, MAPK, NF-κB, STAT3, and androgen receptor signaling. However, mechanistic overlap should not be assumed to indicate clinical synergy. It may instead result in neutral effects, treatment interference, or altered toxicity. For example, reciprocal regulation between androgen receptor signaling and the PI3K/AKT/mTOR pathway is an established mechanism of treatment adaptation and resistance in prostate cancer [5,53]. Additional modulation of these pathways by a nutraceutical may theoretically influence tumor-associated signaling, but the direction and clinical relevance of such effects cannot be predicted from preclinical findings alone. Similar considerations apply to renal cell carcinoma, in which VEGFR-directed kinase inhibitors and immune checkpoint inhibitors act within interconnected angiogenic, immune, and metabolic networks [18,19]. Nutraceutical–drug interaction studies should therefore assess not only metabolic enzymes and transporters but also potential effects on treatment efficacy, immune function, organ toxicity, and patient nutritional status.

Bioavailability and human exposure represent additional major translational constraints. Many polyphenols and triterpenoids exhibit limited oral absorption, rapid first-pass metabolism, extensive conjugation, and substantial interindividual variability. As a result, the predominant circulating or tissue-level chemical species may differ considerably from the unconjugated parent compounds commonly examined in experimental models [240,241]. Human studies should therefore quantify the parent compound and relevant circulating, urinary, or tissue-level metabolites, where appropriate, and determine whether these species reach concentrations compatible with the proposed biological effects. Dose–exposure relationships, absorption and metabolic profiles, target-compartment distribution, formulation characteristics, nutritional status, and variability among target populations should also be considered. Without such information, extrapolation from experimental concentrations to human biological relevance remains speculative.

Evidence from epidemiological and interventional studies further cautions against assuming that a biologically plausible nutrient or bioactive compound will necessarily provide preventive or therapeutic benefit. Vitamin D research, for example, demonstrates that strong mechanistic plausibility does not consistently translate into improved cancer-related outcomes and that supplementation effects may be population-dependent, neutral, or inconsistent [57,242]. Human efficacy or health-function evidence must therefore be evaluated separately from mechanistic findings, bioavailability, and changes in surrogate biomarkers. The immediate priority is not to establish anticancer efficacy but to determine whether a nutraceutical can be manufactured consistently, administered reproducibly, and studied safely in an appropriately defined human population. Before efficacy-oriented investigation, studies should establish the chemical identity, purity, stability, and batch-to-batch consistency of the preparation; validate analytical methods for the parent compound and relevant metabolites; characterize absorption, metabolism, and dose–exposure relationships; evaluate safety and tolerability; and assess clinically relevant nutraceutical–drug interactions. Early human studies should prioritize bioavailability, tolerability, systemic and target-compartment exposure, and compatibility with standard treatment. Mechanism-matched biological-response biomarkers may be included when they are prespecified and supported by adequate exposure data, but they should not be interpreted as evidence of clinical benefit. Only after product quality, human exposure, safety, tolerability, and treatment compatibility have been demonstrated should controlled studies assess clinical efficacy or health-function outcomes in a clearly defined target population. Until these requirements are fulfilled, nutraceuticals should be regarded as investigational nutritional bioactives rather than as established adjunctive anticancer therapies.

## 6. An Exposure- and Evidence-First Research Agenda for Nutraceuticals in Uro-Oncology

The main limitation of nutraceutical research in uro-oncology is not the absence of additional mechanistic observations, but the gap between experimental biological activity and clinically interpretable evidence. Numerous food- and botanical-derived compounds have been reported to influence redox balance, inflammatory signaling, angiogenesis, metabolism, apoptosis, and treatment-response pathways. These findings generate research hypotheses for research but do not demonstrate that the products treat cancer. Future development should therefore follow a staged, evidence- and exposure-first pathway centered on preparation quality, human exposure, safety, treatment compatibility, and mechanism-linked biomarker activity. Clinical outcome-oriented investigation should be considered only after these prerequisites have been satisfied and should not be interpreted as support for commercial disease-treatment claims. The sequence proposed below begins with preparation and analytical characterization, followed by assessment of bioavailability, absorption, and metabolism; safety and interactions; exposure-linked biological responses; limited proof-of-mechanism studies; and, only when justified, efficacy-oriented clinical investigation. Progression between stages should be conditional rather than automatic. Failure to demonstrate adequate exposure, acceptable safety, or tumor-relevant target engagement should preclude advancement to the next stage. Progression should also require concordance among exposure, target engagement, circuit modulation, and phenotype. Measured concentrations of the parent compound and relevant metabolites in plasma, tumor tissue, or urine should be compatible with those required for the proposed effect; the primary biomarker should be proximal to the claimed target rather than a nonspecific stress or cell-death marker; and pathway modulation should be biologically relevant to the selected tumor subtype. Where experimentally feasible, specificity should be supported by genetic perturbation, pharmacological comparison, rescue experiments, or orthogonal validation. A reduction in tumor-cell viability accompanied only by increased reactive oxygen species, caspase activation, altered inflammatory cytokines, or other common cancer-associated readouts should not be regarded as sufficient evidence of network control. Such findings should support further mechanistic investigation but should not, in and of themselves, justify efficacy-oriented clinical testing. Advancement should instead depend on demonstrating that a defined and achievable exposure engages a compound-specific target, alters the intended disease-defining circuit, and produces a biologically interpretable downstream response.

Precision nutraceutical studies should not use a broad and interchangeable panel of pharmacodynamic biomarkers. Biomarker selection should instead follow a compound–subtype–compartment–readout framework. Each study should prospectively define: the chemically characterized compound or formulation being tested; the molecular or metabolic feature used for patient selection; the biological compartment in which the parent compound and relevant metabolites will be measured; the single primary pharmacodynamic pathway matched to the proposed mechanism; and predefined criteria for progression or termination. Secondary markers may be included to assess pathway coherence, but they should not substitute for the primary target-engagement endpoint.

Accordingly, *p*-AKT, *p*-mTOR, and *p*-S6 should be prioritized only when the candidate is proposed to modulate PI3K/AKT/mTOR signaling and the selected tumor demonstrates baseline activity of that pathway. NF-κB signatures should be used only for compounds with an exposure-supported inflammatory-transcription hypothesis. Cleaved caspase-3, cleaved PARP, Ki-67, oxidative-stress markers, and epithelial–mesenchymal transition markers should generally be considered downstream supportive endpoints rather than primary evidence of target engagement. CD8^+^ T-cell infiltration, PD-L1 expression, and related immune markers should be included only when the intervention has a defined immune mechanism and the study is conducted in an immunologically relevant treatment context.

This framework limits each trial to a biologically coherent hypothesis rather than attempting to demonstrate simultaneous effects across multiple signaling pathways. Advancement should require concordance among patient selection, verified exposure in the relevant compartment, modulation of the prespecified primary pathway, acceptable safety, and absence of clinically meaningful treatment interference.

This framework must also remain disease-specific. The required development sequence differs substantially across diseases. PCa and BCa provide the most appropriate settings for molecularly selected Stage 3–4 window studies because prostate tissue, urine, urothelial tissue, and presurgical biomarkers can be assessed directly. RCC requires formulation-specific exposure, characterization of the active metabolite, renal disposition, and interaction testing with VEGFR-, mTOR-, and immune checkpoint-directed therapies before tumor-focused investigation. TGCT requires an even more conservative sequence in which non-interference with cisplatin disposition, DNA-damage signaling, and tumor-cell killing is demonstrated before any supportive or adjunctive study is considered. In prostate cancer, studies should account for dynamic interactions among androgen receptor signaling, characterize prostate cancer, PI3K/AKT/mTOR activity, DNA damage response pathways, and treatment-dependent adaptive signaling [5,243]. Therefore, studies evaluating nutraceutical interventions should incorporate biomarkers of AR activity, PTEN or PI3K pathway status, and, where relevant, DNA damage repair alterations. Bladder cancer studies should address molecular heterogeneity involving PI3K/AKT activation, FGFR3- and ERBB-related receptor tyrosine kinase signaling, immune contexture, and emerging epitranscriptomic regulation [11,15]. In ccRCC, VHL loss, HIF-dependent angiogenesis, and interactions among VEGF, MET, mTOR, metabolic, and immune networks are central [8,16,18,239]. These biological and clinical differences should determine the evidence required at each stage.

### 6.1. Preparation and Analytical Characterization

The first requirement for reproducible nutraceutical research is rigorous characterization of the investigated preparation. The term “nutraceutical” encompasses chemically diverse materials, ranging from purified compounds to botanical extracts, food-derived concentrates, nanoformulations, liposomal products, and mixtures containing multiple potentially active constituents. Preparations bearing the same compound name may differ substantially in source, extraction method, purity, stability, excipients, particle size, dissolution properties, and batch-to-batch composition. They should therefore not be assumed to be pharmacologically interchangeable. Studies should report the botanical or dietary source, manufacturing and extraction procedures, chemical identity, purity, contaminant testing, stability, batch consistency, excipient composition, and, where relevant, particle size, surface properties, encapsulation efficiency, and release characteristics. Multi-component preparations should be analyzed for major co-occurring compounds because these constituents may influence absorption, metabolism, toxicity, or biological activity. Formulation-specific information is particularly important for compounds such as curcumin, resveratrol, quercetin, EGCG, and ursolic acid, for which delivery technologies are frequently used to overcome poor solubility or limited systemic exposure.

Validated analytical methods should be established before clinical investigation. These methods should quantify both the administered parent compound and its major metabolites in appropriate biological matrices, including plasma, serum, urine, and, where feasible, tumor or adjacent tissue. Analytical specificity, sensitivity, recovery, matrix effects, and stability should be reported. Without a reliable measurement of exposure, a negative clinical finding cannot be distinguished from inadequate absorption, whereas a positive biomarker change cannot be confidently attributed to the administered compound. Preparation characterization should therefore be regarded as a prerequisite for all subsequent pharmacokinetic, safety, pharmacodynamic, and efficacy studies. Evidence obtained using one formulation should not be generalized to another without comparative bioavailability and compositional data.

### 6.2. Bioavailability, Absorption, Metabolism, and Target-Compartment Exposure

Once a preparation has been adequately characterized, the next priority is to determine whether biologically relevant human exposure can be achieved. Dose-ranging studies should define absorption, time to maximum concentration, peak and total systemic exposure, elimination half-life, accumulation during repeated administration, interindividual variability, and the relative abundance of the parent compound and its metabolites. This step is essential because many polyphenols and related nutraceuticals undergo extensive intestinal and hepatic metabolism. Glucuronidation, sulfation, methylation, oxidation, reduction, and microbiota-dependent biotransformation may result in circulating molecular species that differ substantially from the unconjugated compounds used in cell-culture experiments [240,241]. The parent compound may represent only a small fraction of systemic exposure, while conjugated or microbiota-derived metabolites may predominate. Findings obtained with high micromolar concentrations of free aglycones therefore have limited translational relevance unless comparable concentrations can be demonstrated in human plasma or target tissue.

The biological activity of major metabolites should therefore be evaluated directly rather than assuming that they reproduce the activity of the parent compound. Where deconjugation within tissues is proposed as a mechanism, this process should be demonstrated experimentally and linked to quantitative exposure data. Pharmacokinetic priorities should differ according to tumor type. In prostate cancer, systemic exposure should ideally be complemented by measurement of parent compounds and metabolites in prostate tissue obtained during presurgical studies. In bladder cancer, urinary concentration and urothelial exposure may be as relevant as plasma pharmacokinetics because the bladder mucosa is directly exposed to urinary metabolites. Urinary metabolomics may support noninvasive exposure assessment and biomarker discovery, although findings from other cancer settings should be extrapolated cautiously [244,245]. In RCC, both systemic disposition and renal clearance require detailed evaluation, particularly in patients receiving agents with narrow therapeutic indices. In TGCT, pharmacokinetic studies must determine whether nutraceutical exposure alters the disposition of cisplatin or supportive medications. The central question at this stage is not whether a compound shows anticancer activity in an experimental model, but whether the molecular species responsible for that activity can be generated at an adequate and tolerable concentration in humans.

The exposure- and evidence-based framework should be operationalized as an exposure–species–compartment sequence. First, the administered preparation and its chemical stability should be defined. Second, the parent compound, phase II conjugates, microbial metabolites, and other potentially active species should be quantified in plasma and urine. Third, the chemical species reaching the disease-relevant compartment—prostate tissue, bladder mucosa or tumor, RCC tissue, or systemic circulation in TGCTs—should be identified. Fourth, measured target-site concentrations should be compared directly with metabolite-specific concentration–response relationships generated in disease-appropriate models. Only then should target engagement and circuit-level biological activity be evaluated.

Formulation optimization, physiologically based exposure modeling, organoid systems, omics-based analyses, and advanced human-relevant experimental models should be used to close a predefined exposure –response gap rather than being presented as generic solutions to poor bioavailability. A formulation should advance only if it reproducibly increases the relevant chemical species in the intended compartment without causing unacceptable toxicity or treatment interactions. Kim et al. [246] and Aljabali et al. [247] emphasized that improved delivery must be integrated with mechanistic validation, exposure characterization, and predictive experimental systems to strengthen clinical translation.

Progression criteria should therefore require concordance among preparation identity, administered dose, systemic exposure, metabolite profile, target-compartment concentration, target engagement, and downstream biological response. Failure at any of these levels should preclude efficacy-oriented clinical development.

Exposure assessment should also anticipate treatment-related changes in nutraceutical disposition and reciprocal effects on anticancer-drug exposure. Candidate preparations should therefore be screened for inhibition or induction of clinically relevant CYP enzymes, UGT-mediated glucuronidation, and clinically relevant transporters before progression to combination studies. Because phase I metabolism, conjugation, and transporter activity may act concurrently, an observed exposure change should not automatically be attributed to a single pathway. Formulation-specific interaction studies are particularly important for concentrated extracts and enhanced-delivery systems, which may produce exposures substantially greater than those achieved through habitual dietary intake [248,249,250]. CYP inhibition and induction are major determinants of drug exposure, while UGT inhibition or dual substrate inhibition has also been associated independently with increased concern for drug-induced liver injury.

### 6.3. Safety Tolerability, and Clinically Relevant Interaction Assessment

Safety assessment should precede claims of anticancer efficacy and should be conducted in the context of the intended oncologic regimen. Tolerability in healthy volunteers or non-cancer populations cannot be extrapolated directly to patients receiving chemotherapy, targeted therapy, PARP inhibitors, or immune checkpoint inhibitors. Concentrated extracts, purified compounds, and bioavailability-enhanced formulations may produce systemic exposures substantially greater than those associated with habitual dietary intake and may therefore have distinct toxicological and interaction profiles. Evaluation should be specific to the formulation and treatment context and should document the exact product composition, dose, duration, exposure to the parent compound and relevant metabolites, renal and hepatic function, concomitant medications, and the potential for clinically relevant nutraceutical–drug interactions affecting treatment exposure, biological activity, or toxicity. Early studies should assess dose-related adverse events, hepatic and renal function, hematologic parameters, gastrointestinal tolerability, cardiovascular effects, electrolyte balance, coagulation, endocrine activity, and immune-related toxicity, based on the compound’s known or proposed pharmacology.

#### 6.3.1. Metabolic and Transporter-Mediated Interactions

Formal assessment of nutraceutical–drug interactions is essential because uro-oncology patients commonly receive multidrug regimens, including targeted agents, chemotherapy, immunotherapy, supportive medications, and treatments for comorbidities [132,237]. CYP-mediated oxidation is particularly relevant, as CYP3A4/5 contributes to the disposition of many orally administered anticancer agents, while CYP2C8 is relevant to taxane metabolism and selected drug–drug interaction scenarios [132,249]. Inhibition of CYP3A4, CYP2C8, CYP2C9, CYP2C19, or CYP2D6 by nutraceutical-derived preparations may increase anticancer-drug exposure and toxicity, whereas enzyme induction may reduce exposure and compromise treatment activity. The direction and magnitude of these effects may vary according to dose, formulation, duration, and the relative abundance of the parent compound and circulating metabolites [237,249].

UGT-dependent glucuronidation should be considered alongside CYP metabolism rather than as a secondary pathway. UGT enzymes contribute to the elimination of many drugs, active metabolites, and xenobiotic- or phytochemical-derived compounds. UGT inhibition has been associated with increased concern for drug-induced liver injury, and CYP–UGT interactions may produce exposure changes that cannot be predicted from either pathway alone. For CYP2C8 substrates, for example, interaction magnitude may depend on the relative contributions of oxidative and glucuronidation pathways to total clearance [248,250]. Candidate nutraceuticals with CYP3A4, CYP2C8, or UGT inhibitory potential, or dual substrate–inhibitor behavior, should therefore undergo structured interaction screening before combination with oral anticancer therapies.

P-gp and BCRP are also clinically relevant because they regulate intestinal absorption, biliary and renal efflux, tissue penetration, and blood–brain barrier transport. Their inhibition may increase systemic or tissue exposure and toxicity, whereas increased efflux may reduce oral bioavailability and target-compartment delivery [251,252]. Transporter effects should be interpreted in conjunction with CYP and UGT liabilities, as simultaneous modulation of metabolism and efflux may determine the net clinical interaction [248,250,252].

This principle is particularly important for AR-pathway inhibitors, which are not pharmacologically interchangeable. Enzyme induction by an AR-directed agent does not necessarily predict its overall transporter effect. In patients with metastatic castration-resistant prostate cancer, enzalutamide modestly increased digoxin exposure but did not alter rosuvastatin exposure, consistent with mild P-gp inhibition and limited BCRP interaction under the conditions studied [253]. Interaction assessment should therefore be performed for the specific AR-pathway agent and formulation rather than extrapolated across the class.

#### 6.3.2. Treatment-Specific Interaction Risks

Interaction assessment is particularly important in renal cell carcinoma, where VEGFR TKIs frequently depend on CYP3A- and transporter-mediated disposition and possess clinically important cardiovascular, hepatic, gastrointestinal, and dermatologic toxicities [254,255,256]. Inhibition of metabolism or efflux may increase exposure and aggravate hypertension, hepatotoxicity, diarrhea, hand–foot syndrome, bleeding, heart failure, or QT-related toxicity, whereas induction may reduce therapeutic exposure [254,256]. Although hypertension is a prominent class effect, pharmacovigilance data indicate that the relative risks of cardiomyopathy, heart failure, and QT prolongation differ among individual agents [257].

Nutraceutical preparations with CYP3A-modulating, transporter-inhibiting, antihypertensive, antiplatelet, cardiotoxic, or hepatotoxic potential should not be combined with VEGFR TKIs without treatment-specific assessment. Monitoring should include blood pressure, liver and renal function, electrolytes, bleeding symptoms, and electrocardiographic evaluation where clinically indicated. Because VEGFR TKI interaction burdens vary across agents, treatment selection should consider both efficacy and the total concomitant medication and supplement profile (Table 4). The most practice-relevant interaction mechanisms and safety implications for selected uro-oncology treatment contexts are summarized in Table 4.

AR-pathway inhibitors may induce or inhibit CYP enzymes and transporters, thereby altering the exposure of supportive medications, other anticancer agents, and coadministered nutraceuticals. Conversely, concentrated supplements affecting CYP3A, CYP2C8, P-gp, or BCRP may modify AR-directed treatment exposure. Enzalutamide, apalutamide, abiraterone, and darolutamide should therefore be evaluated separately rather than treated as a uniform class. Medication and supplement reconciliation should be repeated at treatment initiation, during dose modification, and when unexpected toxicity or loss of response occurs. In patients receiving abiraterone, hepatic function, blood pressure, potassium levels, and fluid retention require particular attention.

Broad modulation of AR and PI3K/AKT/mTOR signaling may also produce context-dependent pharmacodynamic effects. Thus, experimental evidence that a nutraceutical suppresses AR- or PI3K-related signaling should not be automatically interpreted as evidence of safety or benefit when coadministered with AR-directed therapy.

Docetaxel and cabazitaxel rely substantially on CYP3A-mediated clearance. CYP3A inhibition may increase taxane exposure and the risks of neutropenia, mucositis, diarrhea, hepatic toxicity, infection, and treatment interruption, whereas CYP3A induction may reduce exposure and antitumor activity [264,265]. Preclinical evidence of taxane sensitization should not justify clinical coadministration without formal pharmacokinetic and toxicity evaluation. Studies combining nutraceuticals with taxanes should include serial blood counts, hepatic assessment, gastrointestinal toxicity monitoring, infection surveillance, and evaluation of peripheral neuropathy [264,265]. Cisplatin-related interaction risk is governed less by CYP metabolism than by renal handling, hydration, electrolyte balance, DNA-damage signaling, and overlapping nephrotoxic, neurotoxic, ototoxic, and hematologic effects [213,214,263]. Botanicals with nephrotoxic, diuretic, antioxidant, or poorly characterized mineral components may interfere with renal safety, hydration protocols, or supportive care [213,214]. Although platinum compounds may show mechanistic synergy with PARP inhibitors or immune checkpoint inhibitors, these effects are biomarker- and context-dependent and may be accompanied by additional toxicity [263]. In testicular germ cell tumors, where cisplatin-based treatment is frequently curative, the threshold for accepting interaction risk should be especially high [21,22,209]. Any adjunctive nutraceutical should be shown not to reduce cisplatin exposure, platinum–DNA adduct formation, DNA-damage signaling, apoptosis, or tumor-cell killing, and should not increase renal, auditory, neurologic, or hematologic toxicity [213,214,263]. Absence of overt adverse events alone is insufficient to establish non-interference.

PARP inhibitors differ in their metabolic and transporter liabilities. Olaparib is susceptible to CYP3A inhibition and induction, whereas talazoparib exposure is influenced by P-gp activity and renal function [266,267]. Nutraceutical modulation of CYP3A, P-gp, BCRP, renal clearance, or hematopoiesis may therefore increase cytopenias, fatigue, gastrointestinal toxicity, or systemic exposure, or may reduce therapeutic activity [237,266]. Baseline and serial blood counts, renal and hepatic evaluation, and treatment-specific interaction screening should be incorporated into combination studies [266,267]. Particular caution is required when PARP inhibitors are combined with DNA-damaging chemotherapy because PARP trapping and impaired DNA repair may amplify dose-limiting myelosuppression and other toxicities [263,266]. PARP inhibitor–chemotherapy combinations should therefore be distinguished from PARP inhibitor–ICI combinations, for which the principal rationale is immunobiological rather than solely pharmacokinetic [268,269,270].

Classical CYP-, UGT-, and transporter-mediated interactions are less prominent with monoclonal antibody immune checkpoint inhibitors. The principal concerns are pharmacodynamic and immunological. Immune-active, anti-inflammatory, or microbiome-modifying nutraceuticals may alter antigen presentation, immune-cell activation, inflammatory signaling, or the efficacy and toxicity of checkpoint blockade [262,268,269,270]. In addition, hepatotoxic, nephrotoxic, dermatologically active, or gastrointestinal botanical products may mimic or exacerbate immune-related hepatitis, nephritis, severe skin reactions, or colitis, thereby complicating identification of the responsible agent [262].

Checkpoint inhibition may also alter immune responses to concomitant medications, thereby contributing to severe immune-mediated reactions. Supplement use should therefore be documented before ICI initiation and reassessed whenever a suspected immune-related adverse event occurs [262]. PARP inhibition may enhance DNA damage, immune priming, and PD-L1 expression, providing a rationale for PARP inhibitor–ICI combinations; however, this rationale does not establish that an additional nutraceutical will improve efficacy or remain immunologically neutral [269,270].

In bladder cancer, the same caution applies when nutraceuticals are combined with platinum chemotherapy, gemcitabine, FGFR inhibitors, antibody–drug conjugates, or ICIs. Preclinical chemosensitization signals require validation of pharmacokinetics, safety, and non-interference.

#### 6.3.3. Actionable Clinical Safeguards

Before a nutraceutical is combined with anticancer therapy, the product name, manufacturer, composition, formulation, dose, frequency, duration, and treatment indication should be documented. The oncology team should then assess:

1. CYP and UGT inhibition or induction potential;
2. P-gp and BCRP substrate or inhibitor liability;
3. Renal, hepatic, hematologic, cardiovascular, bleeding, electrolyte, and immune toxicity;
4. Overlap with the adverse-effect profile of the intended anticancer regimen;
5. Potential effects on anticancer-drug exposure or efficacy;
6. The availability of validated analytical methods for measuring the parent compound and relevant metabolites.

New concentrated supplements should not be initiated during systemic treatment without review by the treating oncologist or an oncology pharmacist. Unexplained toxicity, altered laboratory values, loss of treatment response, or immune-related symptoms should prompt reassessment of the entire medication and supplement profile. When a combination is scientifically justified, early-phase studies should include measurements of anticancer-drug exposure, quantification of nutraceutical parent compounds and metabolites, predefined organ-specific safety assessments, and explicit criteria for dose interruption or discontinuation. Evaluation should be conducted within the relevant disease and treatment context rather than inferred from dietary exposure, healthy-volunteer studies, or another anticancer regimen. Only preparations with reproducible composition, acceptable treatment-context-specific safety, measurable exposure, and no clinically meaningful interaction signal should proceed to tumor-directed biological-response or efficacy-oriented investigation. In the absence of formal interaction data, concentrated nutraceutical products should be considered investigational exposures rather than routinely safe adjuncts.

### 6.4. Exposure-Linked Biomarker and Biological-Response Assessment

After adequate exposure and tolerability have been demonstrated, studies may investigate whether a nutraceutical produces the proposed biological effect at achievable human concentrations. Biological-response assessment should be explicitly linked to human exposure data. Interpreting a biomarker change is difficult when the concentrations, chemical forms, and tissue availability of the parent compound and relevant metabolites at the target site are unknown. Biomarkers should be selected based on the malignancy’s dominant signaling architecture and the proposed mechanism of the compound. In prostate cancer, relevant measures may include AR-regulated transcription, PSA-related molecular responses, PTEN status, PI3K/AKT/mTOR pathway activity, DNA damage response alterations, and treatment-stage-specific AR–PI3K feedback [5,243]. However, changes in serum PSA alone should not be interpreted as proof of antitumor efficacy without accompanying evidence of tissue exposure and pathway modulation. In bladder cancer, pharmacodynamic assessment may incorporate FGFR3- or ERBB-associated signaling, PI3K/AKT pathway activity, NF-κB-related transcription, EMT markers, proliferation indices, apoptosis markers, and immune contexture [11]. Emerging regulatory mechanisms, including m5C-, m1A-, and m7G-related RNA modifications, may contribute to disease biology, but their utility as intervention-guiding biomarkers remains insufficiently validated [15]. In RCC, relevant biomarkers may include HIF-1α- or HIF-2α-associated programs, VEGF signaling, MET activity, modulation of the PI3K/AKT/mTOR pathway, angiogenic signatures, and immune activation states [8,16,18]. Interpretation must account for overlap with the pharmacodynamic effects of standard VEGFR-directed and immune therapies. Potential pathway-specific readouts across tumor types include phosphorylation states of AKT, mTOR, and S6; NF-κB transcriptional signatures; EMT-associated proteins; apoptosis indices such as cleaved caspase-3 and cleaved PARP; angiogenic markers; and immune microenvironmental measures such as CD8-positive cell infiltration and PD-L1 expression.

Biological-response biomarkers should be prioritized hierarchically rather than combined into a broad, interchangeable panel. Each trial should prespecify a single primary target-proximal or circuit-level endpoint that is mechanistically matched to the tested compound or formulation, the tumor’s molecular subtype, the biological compartment in which exposure has been verified, and the relevant clinical context. Additional downstream biomarkers should be designated as secondary or exploratory endpoints. Additional biomarkers should be included only when they evaluate a predefined feedback mechanism, confirm pathway coherence, or clarify resistance. This approach is consistent with precision-oncology frameworks in bladder cancer, which show that molecular selection should be interpreted alongside pathway activation, feedback regulation, subtype context, and resistance mechanisms rather than treated as a stand-alone predictor of response [181,182].

For isoflavone studies in localized prostate cancer, the most actionable framework would combine equol-producer status, measured S-equol exposure, baseline androgen receptor activity, and a prespecified tissue AR-transcriptional endpoint. Akaza et al. [157] reported differences in equol-producer frequency between men with prostate cancer and controls, while Yoshikata et al. [156] demonstrated substantial interindividual variability in blood and urinary equol concentrations and showed that equol-producer status can be characterized using LC–MS/MS. Miyanaga et al. [159] found no significant preventive effect of purified isoflavones in the overall study population, although exploratory analyses suggested possible age-related heterogeneity. Preliminary experimental evidence also indicates that equol may reduce AR- and PSA-related activity in AR-dependent prostate cancer models [158]. Accordingly, serum PSA alone should not be considered evidence of AR target engagement; the primary endpoint should instead comprise AR nuclear localization, a validated AR-transcriptional signature, or expression of predefined AR-regulated genes in prostate tissue. Measurements of *p*-AKT or *p*-S6 should be added only when baseline molecular profiling demonstrates PTEN loss or PI3K/AKT/mTOR pathway activation.

In bladder cancer, biomarker selection should integrate tumor genotype, exposure compartment, and the proposed modulated pathway. FGFR3-altered disease provides a tractable model because FGFR3 activation can engage MAPK and PI3K/AKT/mTOR signaling, although alternative receptor tyrosine kinases and downstream pathway reactivation may mediate resistance [181,182]. For a selected isoflavone hypothesis involving FGFR3/ERBB–PI3K signaling, FGFR3 mutation, fusion, or validated overexpression should serve as an eligibility criterion, while parent compounds and relevant metabolites should be quantified in timed urine and paired bladder tissue. If the proposed mechanism involves PI3K/mTOR signaling, *p*-AKT and *p*-S6 would represent more informative primary or pathway-coherent endpoints than a broad panel of unrelated apoptotic and inflammatory markers.

For mTOR-directed flavonoid hypotheses, *p*-S6 should generally be prioritized as a proximal pathway readout. Iida et al. [183] showed that luteolin inhibited bladder cancer growth in experimental models by modulating mTOR signaling, supporting *p*-S6 as a candidate pharmacodynamic endpoint and p21 as a mechanism-coherent secondary marker. Similarly, the analysis by Khan et al. [185] provides a mechanistic rationale for evaluating PI3K/AKT/mTOR-related endpoints in studies of curcumin- or resveratrol-class compounds. Nevertheless, these biomarkers should be assessed only after the relevant parent compound or active metabolite has been demonstrated in urine and bladder tissue at concentrations compatible with the proposed effect. Urinary detection alone establishes luminal-exposure feasibility but not intracellular tumor exposure or pathway engagement.

In RCC safety, treatment compatibility, and human exposure should precede tumor-directed biological-response assessment. The available phytochemical evidence remains predominantly preclinical, with unresolved uncertainties concerning bioavailability, dose–response relationships, tolerability, renal and hepatic disposition, and interactions with standard therapies [206,207,237]. Initial studies should therefore assess adverse events, a tolerable study-dose range, exposure to the parent compound and relevant metabolites, renal and hepatic function, and potential effects on exposure to the specific VEGFR-, mTOR-, or immune-directed regimen. The phase I/II study design described by Lundstam et al. [271], although not evidence of nutraceutical efficacy, illustrates the principle that safety, tolerability, dose escalation, quality, exposure, and interaction characterization should precede efficacy interpretation. Tumor endpoints, such as VHL–HIF–VEGF activity, *p*-S6, or immune signatures, should be introduced only in histologically and therapeutically defined expansion cohorts after adequate exposure and the absence of clinically meaningful drug interactions have been demonstrated. This sequence is appropriate for nutraceutical research and avoids conflating nutrition-al bioactive development with early-phase anticancer drug development.

In testicular germ cell tumors, the primary requirement should be to demonstrate that a nutraceutical intervention does not compromise cisplatin exposure or antitumor activity, rather than to show independent modulation of a nutraceutical-related pathway. Because cisplatin-based therapy is frequently curative, studies should assess whether the intervention alters systemic cisplatin exposure, platinum–DNA adduct formation, DNA-damage signaling, apoptosis, or tumor-cell killing. Chovanec et al. [209] emphasized both the curative success and long-term toxicities of cisplatin treatment, whereas Cocetta et al. [213] and Dasari et al. [214] showed that nutraceuticals or natural products may interact with platinum-based therapy in potentially beneficial, neutral, or detrimental ways. Cleaved caspase-3 and cleaved PARP may therefore be relevant as tumor non-interference endpoints in this setting, but they should not be interpreted as general evidence of nutraceutical anticancer efficacy.

NF-κB signatures, epithelial–mesenchymal transition markers, cleaved caspase-3, cleaved PARP, Ki-67, CD8^+^ T-cell infiltration, and PD-L1 expression should consequently not be used as interchangeable indicators of biological activity. Their inclusion should depend on an exposure-supported compound mechanism and the molecular and therapeutic context of the tumor. NF-κB or EMT endpoints may be informative when inflammatory transcription or epithelial plasticity is the prespecified mechanism, whereas CD8^+^ infiltration and PD-L1 expression should be included only in immunologically relevant studies with a defined hypothesis concerning immune modulation. A biomarker change observed without verified exposure in the relevant target compartment should be interpreted as exploratory. Conversely, measurable exposure in the absence of a prespecified, mechanism-matched biological response supports bioavailability and exposure feasibility but does not demonstrate biological activity or clinical benefit.

Preclinical studies from non-oncologic inflammatory models have identified *p*-AKT, *p*-mTOR, IL-6, and IL-8 as potential indicators of PI3K/AKT/mTOR pathway modulation, but these markers require validation in uro-oncology tumor contexts before being used as definitive pharmacodynamic endpoints [272]. Likewise, network pharmacology studies identify NF-κB, MAPK, PI3K/AKT, and Nrf2 as recurrent convergence nodes for plant-derived metabolites, supporting systems-level biomarker panels while not substituting for direct experimental validation [273]. Nonspecific changes in circulating inflammatory or oxidative stress markers should not, by themselves, be considered evidence of tumor target engagement. Whenever feasible, biological activity should be assessed directly in tumor tissue, urothelial tissue, prostate tissue, urine-derived tumor material, or validated tumor-associated liquid biomarkers. The preferred analysis should establish a quantitative relationship among administered dose, systemic or local exposure, and biomarker response.

### 6.5. A Practical Disease-Specific Precision Roadmap

The precision framework should be operationalized through disease-specific development pathways rather than a universal panel of compounds and biomarkers. Each research program should prospectively define a single molecular context, one chemically characterized formulation, one principal exposure marker, one mechanism-matched biological-response biomarker, one clinical setting, and one primary endpoint. This focused design reduces biological heterogeneity and prevents broad pathway-related findings from being interpreted as evidence of clinical applicability.

In prostate cancer, the most defensible initial setting is localized, androgen receptor-driven disease with PTEN loss or evidence of PI3K/AKT/mTOR pathway activation. These molecular features are clinically relevant because AR and PI3K signaling interact dynamically and may influence treatment response and adaptive resistance [274,275,276]. A standardized, chemically characterized green tea catechin formulation represents a practical candidate because it has the most developed human exposure and tolerability evidence among the nutraceuticals reviewed. Early studies should quantify EGCG and its major methylated and conjugated metabolites in plasma and, where feasible, prostate tissue. In PTEN-deficient tumors, the primary biological-response endpoint should be a proximal marker of PI3K/mTOR pathway activity, such as tissue *p*-S6, rather than serum PSA alone. PSA kinetics and AR-regulated transcriptional markers may be included as secondary supportive endpoints. The most appropriate clinical settings are active surveillance or a presurgical window before prostatectomy, where paired tissue and plasma samples can be obtained. The primary objective should be demonstration of exposure-linked pathway modulation rather than progression-free or overall survival, which would be premature in an initial translational study [277,278].

In bladder cancer, the roadmap should initially focus on prospectively defined luminal-papillary, FGFR3-altered, non-muscle-invasive disease. Molecular subclassification is important because luminal-papillary and basal tumors differ in receptor tyrosine kinase activity, immune context, and treatment sensitivity [11,179,279,280]. A standardized isoflavone or genistein formulation selected based on measurable urinary exposure would provide a testable compound–subtype pairing. Parent compounds and relevant metabolites should be quantified in timed urine and plasma, and, where feasible, in paired urothelial or tumor tissue. In FGFR3-altered tumors, a prespecified FGFR3–MAPK or FGFR3–PI3K pathway marker, such as tissue *p*-ERK or *p*-AKT, should serve as the primary pharmacodynamic endpoint.

A short pre-transurethral resection window is the most appropriate early clinical setting because it permits direct assessment of urinary exposure, tissue delivery, local tolerability, and pathway modulation. Initial studies should use exposure-linked biomarker change as the primary endpoint; recurrence reduction should be reserved for later controlled trials after bioavailability, exposure feasibility, and target engagement have been demonstrated. For renal cell carcinoma, the roadmap should remain conservative because no nutraceutical has established clinical activity, and validated predictive biomarkers remain limited. Clear-cell RCC with VHL–HIF–VEGF-dominant biology provides the most coherent mechanistic entry point, but molecular selection should initially be considered exploratory rather than clinically established [281,282]. An analytically defined, bioavailability-enhanced curcumin formulation may be considered only after demonstrating acceptable CYP3A, UGT, P-gp, and BCRP interaction profiles. Parent compounds and active metabolites should be quantified in plasma and, when feasible, in renal and tumor tissue. The primary biological response endpoint should be selected based on the prespecified mechanism. For example, an HIF-2α/VEGF-associated signature may be appropriate for an angiogenesis-related hypothesis, whereas tissue *p*-S6 may be used when the proposed mechanism involves mTOR signaling. The preferred initial setting is a short presurgical window in untreated localized clear-cell RCC rather than in combination with a VEGFR TKI or immune checkpoint inhibitor. The primary endpoints should be safety, formulation-specific pharmacokinetics, renal disposition, tumor exposure, and exposure-linked pathway modulation. Tumor response or survival should not be designated as primary endpoints until clinically relevant nutraceutical–drug interactions have been excluded and exposure-linked biological-response feasibility has been demonstrated.

The TGCT roadmap should be explicitly safety- and non-interference-oriented. Because cisplatin-based treatment is highly curative, no tumor-directed nutraceutical should enter clinical investigation solely on the basis of pathway modulation. The most relevant biological group for disease monitoring is non-teratomatous, miR-371a-3p-positive germ cell tumor, because miR-371a-3p is an emerging marker of viable germ cell malignancy but does not reliably detect teratoma [283,284]. At present, no specific nutraceutical formulation can be considered clinically ready for adjunctive TGCT treatment. Only a chemically standardized candidate with prior evidence of cisplatin non-interference should advance. Plasma concentrations of the parent compound and its metabolite should be measured concurrently with cisplatin pharmacokinetics. The primary pharmacodynamic assessment should evaluate preservation of platinum–DNA adduct formation and DNA-damage signaling, such as γH2AX, rather than broad oxidative-stress or inflammatory biomarkers. Serum tumor markers and miR-371a-3p may be included as secondary measures for disease monitoring. Any initial investigation should be conducted in a carefully controlled supportive-care setting and should prioritize safety, renal and reproductive toxicity, and preservation of cisplatin antitumor activity. The primary endpoint should be the absence of clinically meaningful change in cisplatin exposure, dose intensity, tumor response, or treatment completion. Fertility-related outcomes may be assessed only as secondary supportive endpoints after antitumor non-interference has been established. Overall, the practical framework requires that each trial test a single coherent compound–disease–mechanism hypothesis. Each research program should prospectively define a single molecular context, one chemically characterized formulation, one principal exposure marker, one primary mechanism-matched biological-response biomarker, and one clinical objective. Progression to efficacy-oriented testing should require reproducible exposure in the relevant target compartment, consistent modulation of the prespecified biomarker, acceptable safety and tolerability, and no clinically meaningful interference with established treatment.

### 6.6. Efficacy-Oriented Investigation

The disease-specific roadmap defines the minimum translational sequence required before efficacy-oriented investigation. The proposed compound–subtype pairings should not be interpreted as treatment recommendations; rather, they illustrate how a precision-oriented hypothesis can be translated into a testable combination of a chemically characterized formulation, a principal exposure marker, a mechanism-matched biological-response endpoint, a defined clinical setting, and a prespecified progression criterion. Failure to demonstrate acceptable safety and tolerability, reproducible target-compartment exposure, or the prespecified biological response should preclude progression to efficacy testing.

Efficacy-oriented clinical trials should represent the final rather than the initial stage of nutraceutical development. Progression to such studies should be supported by a coherent body of evidence demonstrating that the investigated intervention is based on a reproducible, chemically defined formulation; that validated analytical methods are available to quantify the parent compound and its relevant metabolites; and that biologically meaningful, reproducible human exposure can be achieved. In addition, the major active metabolites and their distribution in plasma, urine, and target tissue should be characterized; acceptable short- and long-term safety and tolerability should be established; and clinically meaningful nutraceutical–drug interactions affecting anticancer-treatment exposure, biological activity, or toxicity should be excluded [237,249,266,267]. Evidence of tumor-relevant target engagement at achievable exposure levels, along with a disease-specific biological rationale supported by human data, should also be available before initiating efficacy-oriented investigation. Few, if any, currently studied nutraceuticals satisfy all of these requirements in uro-oncology. Therefore, the justification for efficacy trials should not rely solely on extensive in vitro pathway modulation, positive findings in animal models, epidemiological associations, or the availability of an enhanced-delivery formulation. Although such observations may provide a basis for further investigation, they do not establish that the relevant molecular effects can be reproduced safely at clinically achievable concentrations in humans [160,161,246,247].

When efficacy-oriented studies eventually become justified, their design should reflect the molecular, clinical, and therapeutic context of the relevant malignancy. In prostate cancer, patient stratification may incorporate treatment stage, androgen receptor activity, PTEN status, PI3K pathway alterations, and defects in DNA damage response pathways [5,243]. In bladder cancer, molecular subtype, FGFR3 or ERBB alterations, PI3K pathway activation, and tumor immune contexture may be relevant to patient selection and biomarker interpretation [11]. In RCC, the study design should consider VHL/HIF biology, angiogenic and immune phenotypes, MET pathway activity, and concurrent systemic therapies [8,16,18]. In TGCTs, efficacy-oriented adjunctive studies should remain exceptional and would require particularly robust evidence that the intervention does not alter the pharmacokinetics, toxicity, or antitumor activity of curative cisplatin-based chemotherapy [21,22,209,213,214,263]. Combination strategies should not be developed solely through empirical compound-plus-drug testing. Because many nutraceuticals reportedly act on signaling pathways targeted by approved anticancer agents, combinations may result in antagonism, altered drug exposure, compensatory pathway activation, or unexpected toxicity as readily as they may produce synergy [213,214,237,249]. Validated pharmacokinetic and pharmacodynamic models, disease-stage-specific biological evidence, and formal interaction testing should therefore support rational combination studies. The selection of clinical endpoints should also correspond to the maturity of the available evidence. Early clinical investigations should prioritize safety, systemic and tissue exposure, metabolite characterization, and demonstration of target engagement. Endpoints such as tumor response, recurrence, progression-free survival, and overall survival should be evaluated only after bioavailability and exposure feasibility, safety and tolerability, target-compartment delivery, treatment compatibility, and exposure-linked biological activity have been established, and when a clinically meaningful effect size can be reasonably justified. Nutraceutical interventions should be assessed against appropriate control groups and must not replace, delay, or compromise established evidence-based oncologic treatment. Collectively, this exposure- and evidence-based framework defines nutraceutical development as a conditional, stepwise process in which progression depends on reproducible human exposure, mechanism-matched biological responses, acceptable safety, and absence of clinically meaningful treatment interference. It therefore avoids a direct transition from preclinical mechanistic plausibility to claims of clinical benefit. The immediate priority is to determine whether experimentally reported molecular effects can be reproduced at achievable and safe human exposure levels using chemically characterized and standardized formulations. Only compounds that successfully meet sequential requirements relating to preparation quality, pharmacokinetics, metabolism, safety, interaction assessment, tissue distribution, and tumor-relevant pharmacodynamics should advance to efficacy-oriented clinical investigation. Such methodological discipline is essential for narrowing the substantial gap between experimental pathway modulation and clinically meaningful benefit in uro-oncology. Accordingly, the translational threshold for nutraceutical development in uro-oncology should not be limited to additional in vitro activity alone, but should also include demonstration of formulation quality, clinically relevant parent-compound and metabolite exposure, mechanism-linked pharmacodynamic activity, acceptable treatment-context-specific safety, and absence of meaningful interference with standard anticancer therapy.

## 7. Limitations of the Evidence Base and Review Approach

Several limitations should be considered when interpreting this review. First, this article is a structured expert review rather than a systematic or scoping review. Although a predefined and transparent literature-identification strategy was used, the aim was to prioritize mechanistically informative and translationally relevant studies rather than to provide exhaustive coverage of every dietary or botanical compound. Accordingly, selection and interpretive bias cannot be excluded. Second, the underlying evidence is highly heterogeneous. Studies differ in botanical source, chemical composition, purity, formulation, dose, route of administration, treatment duration, experimental model, and outcome selection. Preparations described under the same compound name may therefore have markedly different pharmacokinetic, metabolic, and biological properties. This variability limits direct comparison across studies and precludes meaningful quantitative synthesis. Third, much of the evidence derives from in vitro experiments using unconjugated parent compounds at concentrations that may not be achievable in humans. Many polyphenols undergo extensive intestinal, hepatic, and microbiota-dependent metabolism, producing glucuronidated, sulfated, methylated, or microbial metabolites that may differ substantially from the parent compound in exposure and biological activity. Mechanistic findings obtained without formulation-specific pharmacokinetic data or verification of exposure in plasma, urine, or target tissue should therefore be regarded as hypothesis-generating rather than clinically applicable. Fourth, the maturity and relevance of the evidence vary across malignancies. Prostate and bladder cancers offer comparatively tractable settings for presurgical, tissue-based, or urine-based translational studies. By contrast, RCC remains limited by uncertainties concerning systemic and renal disposition, target-tissue exposure, and interactions with VEGFR-directed agents, mTOR inhibitors, and immune checkpoint therapies. Evidence in TGCT is predominantly preclinical or indirect and must be interpreted with particular caution, as cisplatin-based treatment is frequently curative. Findings should therefore not be extrapolated across tumor types without consideration of disease biology, treatment context, organ function, and the relevant exposure compartment. Fifth, the evidence-domain categories used in this review—prevention signal, mechanistic plausibility, bioavailability and exposure feasibility, exposure-linked biomarker activity, and clinical benefit—describe the predominant nature and translational maturity of the available evidence. They are not formal risk-of-bias assessments or certainty-of-evidence grades. Systematic or scoping reviews focused on individual compounds, malignancies, or clinical settings will be required to evaluate study quality and evidence certainty using design-appropriate appraisal tools. An additional limitation concerns terminology. Experimental reports frequently use terms such as “anticancer,” “therapeutic,” or “chemopreventive” to describe changes in cell viability, pathway activity, tumor growth, oxidative stress, or inflammatory signaling in model systems. In this review, such observations are described according to their evidentiary level as mechanistic, pharmacologic, exposure-related, pharmacodynamic, supportive, or investigational unless validated clinical outcomes are available. Modulation of a cancer-relevant pathway or reduction in tumor growth in an experimental model should not be interpreted as evidence that a nutraceutical prevents, treats, or cures cancer. Finally, this review does not support nutraceuticals as alternatives to surgery, radiotherapy, systemic anticancer treatment, or evidence-based supportive oncology. The proposed precision-oriented framework is intended solely to guide future research on chemically characterized, exposure-verified, treatment-compatible, and biomarker-informed interventions. Any potential supportive role should be established through appropriately controlled human studies and should not be inferred from preclinical activity alone.

## 8. Conclusions and Future Perspectives

The available evidence provides a biologically plausible but clinically incomplete basis for investigating plant-derived nutraceuticals in uro-oncology. Isoflavones, catechins, curcumin, flavonols, carotenoids, stilbenes, and triterpenoids have been reported to influence cancer-relevant processes, including PI3K/AKT/mTOR, NF-κB, MAPK, HIF-VEGF, androgen receptor signaling, epithelial–mesenchymal transition, apoptosis, redox regulation, and tumor-microenvironmental activity. These observations arise mainly from experimental models and should not be interpreted as evidence that nutraceuticals prevent, treat, or cure urologic cancers. The conclusions of this review are therefore stage-specific. Dietary and epidemiological studies, particularly those involving lycopene and food-derived exposures, provide prevention signals but do not establish causality or tumor-directed benefit. Cell, organoid, animal, and network pharmacology studies support varying degrees of mechanistic plausibility but often rely on unconjugated parent compounds at concentrations that may not be achievable in humans. Available human exposure and presurgical studies primarily demonstrate formulation feasibility, systemic or tissue exposure, and exploratory biomarker modulation. No nutraceutical evaluated in this review has demonstrated validated clinical benefit for the treatment of a uro-oncologic malignancy. The central translational challenge is not simply poor bioavailability but uncertainty about which parent compounds or metabolites reach the relevant biological compartment, whether their concentrations are sufficient to engage the proposed target, and whether such exposure is compatible with established oncologic treatment. Future studies should therefore integrate product standardization, parent-compound and metabolite profiling, systemic and compartment-specific exposure assessment, target proximal biological-response biomarkers, and formal safety and nutraceutical–drug interaction evaluation. Findings obtained with supraphysiological concentrations of a parent compound should not support clinical development when human exposure is dominated by conjugated or microbiota-derived metabolites whose biological activity remains unverified. The proposed precision-oriented research framework provides a disease-specific pathway for addressing these requirements. Prostate and bladder cancers provide comparatively tractable settings for active surveillance, presurgical, pre-TURBT, or perioperative studies in which plasma, urine, and tissue exposure can be linked to predefined pharmacodynamic endpoints. In RCC, formulation-specific exposure, renal disposition, and interactions with VEGFR-directed agents, mTOR inhibitors, and immune checkpoint therapies should be established before tumor-focused investigation. In TGCT, the high curability of cisplatin-based treatment requires an especially conservative approach in which preservation of cisplatin pharmacology, antitumor activity, dose intensity, and treatment completion takes precedence over exploratory nutraceutical modulation.

Nutraceuticals should consequently be positioned as investigational nutritional bioactives or, where supported by appropriate evidence, as potential health-supportive interventions rather than as alternatives to surgery, radiotherapy, systemic anticancer therapy, or evidence-based supportive oncology. Advancement should proceed through clearly defined evidentiary stages, from mechanistic plausibility to bioavailability and exposure feasibility, exposure-linked biomarker activity, and ultimately validated clinical outcomes. Only rigorously controlled human studies demonstrating acceptable safety, treatment compatibility, and clinically meaningful benefit can justify stronger conclusions about their role in contemporary uro-oncology.

## Figures and Tables

**Table 1:** Core signaling hubs, biological implications, and established oncologic treatment contexts across uro-oncology malignancies.

Cancer Type	Dominant Signaling Hubs	Biological Implications	Established Oncologic Treatment Context	References
Prostate cancer (PCa)	Luminal/AR-high, basal/lineage-plastic, and neuroendocrine/AR-low states; AR; PI3K/AKT/mTOR; MAPK; WNT/β-catenin; DDR	Subtype-dependent AR reliance; AR–PI3K crosstalk; lineage plasticity; EMT and metastasis; genomic instability; therapy resistance	Androgen deprivation therapy (ADT); next-generation AR pathway inhibitors; CYP17A1 inhibitors; PARP inhibitors for HRR-deficient disease; PI3K/AKT/mTOR pathway inhibitors and combination strategies	[5,10,11,12,13]
Bladder cancer (BCa)	Luminal papillary/FGFR3-enriched, luminal unstable, basal/squamous, stroma-rich, and neuroendocrine-like states; PI3K/AKT; EGFR/ERBB; p53/RB; PD-L1	Subtype-specific RTK signaling, cell-cycle deregulation, immune/stromal context, invasion, and treatment response	FGFR inhibitors (e.g., erdafitinib); immune checkpoint inhibitors; biomarker-guided therapy; emerging RNA-modification-targeted strategies	[11,14,15]
Renal cell carcinoma (RCC)	ccRCC: VHL/HIF–VEGF; papillary RCC: MET-related biology in subsets; chromophobe/oncocytic RCC: distinct mitochondrial and chromosomal programs; PI3K/AKT/mTOR; immune–angiogenic signaling	Histology-specific angiogenesis, metabolic reprogramming, invasion, immune coupling, and differential therapy sensitivity	VEGFR-targeted TKIs (sunitinib, axitinib); mTOR inhibitors; TKI + immune checkpoint inhibitor combinations; MET-targeted agents	[4,8,16,17,18,19]
Testicular germ cell tumors (TGCT)	Seminoma and nonseminomatous lineages; PI3K/AKT; MAPK; DDR; TP53–MDM2; epigenetic regulators	Histology- and differentiation-dependent proliferation, DNA-damage sensitivity, cisplatin response, and refractory-state vulnerabilities	Cisplatin-based chemotherapy backbone; investigational PARP, mTOR, CDK, and epigenetic inhibitors in refractory/resistant	[20,21,22]

The treatments listed in this table represent established or investigational oncology-directed interventions and should not be interpreted as therapeutic claims for nutraceuticals. Nutraceutical-related pathway effects discussed elsewhere in the manuscript are predominantly mechanistic and do not establish disease-treatment efficacy. Abbreviations: ADT, androgen deprivation therapy; AR, androgen receptor; BCa, bladder cancer; CDK, cyclin-dependent kinase; DDR, DNA damage response; EGFR, epidermal growth factor receptor; EMT, epithelial–mesenchymal transition; FGFR3, fibroblast growth factor receptor 3; HIF, hypoxia-inducible factor; HRR, homologous recombination repair; MAPK, mitogen-activated protein kinase; MDM2, mouse double minute 2 homolog; mTOR, mechanistic target of rapamycin; PARP, poly(ADP-ribose) polymerase; PCa, prostate cancer; PD-L1, programmed death-ligand 1; RCC, renal cell carcinoma; TGCT, testicular germ cell tumor; TKI, tyrosine kinase inhibitor; VEGF, vascular endothelial growth factor; VEGFR, vascular endothelial growth factor receptor; VHL, von Hippel–Lindau; WNT, Wingless/Integrated. The table summarizes dominant signaling networks and clinically relevant therapeutic implications across major uro-oncology malignancies.

**Table 3:** Mechanistic rationale, evidence maturity, and research priorities for plant-derived nutraceuticals in uro-oncology.

Disease and Molecular Context	Candidate Nutraceutical or Formulation	Mechanistic or Supportive Hypothesis	Evidence Maturity	Principal Limitation or Risk	Earliest Justified Research Step and Endpoint	References
PCa: localized AR-active disease; equol-producer status and PTEN context recorded	Chemically defined genistein, daidzein, or S-equol formulation	AR and PI3K/MAPK modulation; AKT–FOXO3a signaling	Preclinical plus limited and mixed human intervention evidence	Variable equol production, formulation, dietary heterogeneity, and inconsistent PSA findings	Presurgical exposure study quantifying the administered parent compound(s) and relevant circulating, urinary, and tissue-level metabolites across serum, urine, and prostate tissue; primary endpoint: tissue AR activity, with *p*-AKT and *p*-S6 assessed only in tumors with baseline PI3K-pathway activation.	[26,58,59,60,61,62,63,64,65,66,67]
PCa: predominantly AR-active localized disease	Standardized EGCG/green tea catechin formulation	AR, PI3K/AKT, NF-κB, MAPK, STAT, and epigenetic signaling	Strong preclinical; limited human tissue and biomarker evidence	Low and variable exposure, formulation heterogeneity, and stage-dependent effects	Short presurgical study with plasma and prostate-tissue exposure to parent catechins and relevant metabolites and one prespecified AR- or PI3K-linked PD endpoint	[29,68,69,70,71,72,73,74,75,76]
PCa: prevention or interception context	Food-matrix or chemically characterized lycopene preparation	Redox and inflammatory regulation; IGF/PI3K-related signaling	Epidemiological and preclinical evidence; mixed human intervention data	Dietary confounding, food-matrix effects, threshold exposure, uncertain prostate delivery	Controlled dietary or formulation study with plasma and prostate-tissue carotenoids and prevention-related biomarkers; no treatment claim	[77,78,79,80,81,82,83]
PCa: exploratory molecularly defined setting	Curcumin, flavonols, resveratrol, quercetin, or UA in formulation-specific studies	NF-κB, PI3K/AKT/mTOR, AR, STAT3, apoptosis, and EMT-related signaling	Predominantly preclinical	Supraphysiological in vitro concentrations, uncertainty regarding the parent compounds and biologically relevant metabolites, poor bioavailability, and interaction risk	Formulation-specific PK, safety, and tissue target-engagement studies before clinical outcome-oriented investigation	[25,42,84,85,86,87,88,89,90,91,92,93,94,95,96,97,98]
BCa: molecular subtype, FGFR3 status, and urinary exposure defined	Standardized EGCG formulation	PTEN/PI3K/AKT, NF-κB, MAPK, EMT, and stemness-related signaling	Strong preclinical; minimal human evidence	Uncertain urinary and intracellular tumor exposure; metabolite relevance	Pre-TURBT window study quantifying parent catechins and relevant metabolites in urine, plasma, and tumor tissue with one PI3K- or NF-κB-linked PD endpoint	[99,100,101,102,103]
BCa: FGFR3-enriched luminal versus TP53/RB1-associated basal context	Chemically defined isoflavones or flavonols	FGFR3/RTK–PI3K/MAPK, cell-cycle, apoptosis, or EMT hypotheses	Limited human biomarker evidence plus preclinical findings	Molecular heterogeneity and predominance of conjugated metabolites in humans	Subtype-enriched presurgical study with FGFR3/TP53/RB1 annotation, urinary and tissue exposure to parent compounds and relevant metabolites, and a subtype-matched PD marker	[104,105,106,107,108,109,110,111,112]
BCa: pathway-defined preclinical setting	Formulation-specific curcumin or UA	NF-κB, PI3K/AKT/mTOR, JAK/STAT, WNT, IGF2, AMPK/JNK, apoptosis, and EMT	Preclinical	Poor bioavailability, uncertain urothelial exposure, and unknown interactions with systemic therapy	Urinary and tissue exposure assessment of parent compounds and relevant metabolites, and short presurgical safety/target-engagement study; formal interaction testing before any chemotherapy combination	[42,113,114,115,116,117,118,119,120,121,122,123,124,125]
RCC: predominantly clear-cell histology	Quercetin/isoquercetin, resveratrol, or other analytically defined formulations	HIF-VEGF, PI3K/AKT/mTOR, ERK/MMP, and inflammatory signaling	Preclinical with limited early human exposure data	Histology-specific biology, renal disposition, CYP/transporter effects, and TKI/ICI interactions	Safety and PK study with renal and tumor exposure to the parent compound and relevant metabolites and formal interaction monitoring; pathway biomarker secondary to safety	[126,127,128,129,130,131,132,133,134]
RCC: exploratory ccRCC setting	Curcumin, EGCG, or related phytochemicals after interaction screening	NF-κB, PI3K/AKT/mTOR, angiogenic, metabolic, or immune-related signaling	Preclinical	Poor exposure and high potential for interaction with VEGFR TKIs or ICIs	Presurgical or human-exposure study focused on safety, exposure to parent compounds and relevant metabolites, and exposure-linked biological activity; tumor-response testing premature	[18,19,125,132,135,136,137,138,139]
TGCT: seminoma and nonseminomatous disease distinguished	No clinically ready candidate; only a standardized formulation with prior cisplatin non-interference evidence	Normal tissue or reproductive support rather than exploratory tumor modulation	Indirect and preclinical	Potential interference with highly curative cisplatin treatment; no validated tumor-specific PK/PD framework	Preclinical assessment of cisplatin PK, platinum–DNA adducts, DNA-damage signaling, tumor-cell killing, and reproductive toxicity before any supportive clinical study	[21,22,43,140]
BCa/RCC receiving ICIs	EGCG, curcumin, or other immunomodulatory phytochemicals	PD-L1, STAT3/NF-κB, T-cell, myeloid, and microbiome-related modulation	Indirect and preclinical	Bidirectional immune effects, altered ICI activity, and immune-related toxicity	Immunocompetent disease-specific models were followed, only when justified, by safety, PK, and immune-PD studies	[141,142,143]

Evidence maturity describes the predominant source and translational stage of the available evidence and is not a formal certainty grade or clinical recommendation. Mechanistic effects are predominantly derived from experimental studies unless explicitly identified as human evidence. “Earliest justified research step” indicates the next methodologically appropriate investigation and does not imply that routine supplementation, combination treatment, or efficacy testing is warranted. Where exposure assessment is proposed, measurements should include the administered parent compound(s) and relevant circulating, urinary, and tissue-level metabolites, as appropriate to the compound, formulation, and target organ; PD, pharmacodynamics; ICI, immune checkpoint inhibitor; TURBT, transurethral resection of bladder tumor; UA, ursolic acid. Abbreviations: AKT, protein kinase B; AMPK, AMP-activated protein kinase; AR, androgen receptor; BCa, bladder cancer; ccRCC, clear cell renal cell carcinoma; CDK, cyclin-dependent kinase; EGCG, epigallocatechin-3-gallate; EMT, epithelial–mesenchymal transition; ERK, extracellular signal-regulated kinase; FGFR3, fibroblast growth factor receptor 3; HIF, hypoxia-inducible factor; IGF, insulin-like growth factor; JAK, Janus kinase; JNK, c-Jun *N*-terminal kinase; MAPK, mitogen-activated protein kinase; MMP, matrix metalloproteinase; mTOR, mechanistic target of rapamycin; NF-κB, nuclear factor kappa B; PCa, prostate cancer; PI3K, phosphoinositide 3-kinase; PK, pharmacokinetics; RCC, renal cell carcinoma; RTK, receptor tyrosine kinase; STAT, signal transducer and activator of transcription; TGCT, testicular germ cell tumor; TKI, tyrosine kinase inhibitor; TURBT, transurethral resection of bladder tumor; VEGF, vascular endothelial growth factor; WNT, Wingless/integrated signaling.

**Table 4:** Practice-relevant interaction mechanisms and safety implications for selected uro-oncology treatment contexts.

Setting	Mechanism Most Relevant to Practice	Actionable Safety Implication
VEGFR TKIs	Disruption of VEGFR2 signaling through direct kinase inhibition or suppression of downstream VEGFR2–NFAT signaling pathways [258,259]	Use caution with concomitant drugs that affect blood pressure, the QT interval, or cardiac function.
VEGFR TKI class	Class toxicities and clinical profiles vary across VEGFR TKIs despite a shared target class [260,261]	Agent selection should incorporate interaction burden, not only efficacy.
Cabozantinib/sunitinib/lenvatinib/vandetanib	FAERS signals include hypertension across classes; stronger signals for heart failure, cardiomyopathy, or QT/TdP with specific agents [257]	Baseline cardiovascular comorbidities and concomitant use of QT-prolonging or cardiotoxic drugs warrant explicit consideration.
ICI + cytotoxic/targeted therapy	Overlapping immune and nonimmune toxicities can be amplified in combinations [262]	Evaluate toxicity additivity and sequence-dependent immune toxicity rather than assuming purely pharmacokinetic interaction.
Cisplatin + PARP inhibitor/ICI	Platinum adds DNA-damage and immunomodulatory effects; synergy is biologically plausible but biomarker dependent [263]	Benefits appear context-specific, so combination claims should be linked to HRD/BRCA-like biology and toxicity surveillance.

## Data Availability

The original contributions presented in this study are included in the article/Supplementary Material. Further inquiries can be directed to the corresponding authors.

## Supplementary Materials

The following supporting information can be downloaded at: https://www.mdpi.com/article/10.3390/nu18152411/s1, Table S1. Database-specific search strategies used for the structured expert review; Table S2 Database-specific search strategies used in PubMed/MEDLINE, Embase, Scopus, and Web of Science Core Collection.

## Abbreviations

AKT, protein kinase B; ALDH1A1, aldehyde dehydrogenase 1 family member A1; AMPK, AMP-activated protein kinase; AP-1, activator protein 1; AR, androgen receptor; ASAP, atypical small acinar proliferation; ATM, ataxia-telangiectasia mutated; BAX, BCL-2-associated X protein; BCa, bladder cancer; BCL-2, B-cell lymphoma 2; BCRP, breast cancer resistance protein; BPH, benign prostatic hyperplasia; BRCA1/2, breast cancer susceptibility genes 1 and 2; ccRCC, clear cell renal cell carcinoma; CCR6, C-C chemokine receptor 6; CD3, cluster of differentiation 3; CD8, cluster of differentiation 8; CDK, cyclin-dependent kinase; COX-2, cyclooxygenase-2; CRPC, castration-resistant prostate cancer; CYP, cytochrome P450 enzyme family, including CYP17A1, CYP2C8, CYP2C9, CYP2C19, CYP2D6, and CYP3A4; DDR, DNA damage response; DNMT, DNA methyltransferase; DNMT1, DNA methyltransferase 1; EGCG, epigallocatechin-3-gallate; EGFR, epidermal growth factor receptor; eIF4E, eukaryotic translation initiation factor 4E; EMT, epithelial–mesenchymal transition; ERBB, Erb-B receptor tyrosine kinase family; ERK, extracellular signal-regulated kinase; FGFR, fibroblast growth factor receptor; FGFR3, fibroblast growth factor receptor 3; GTCs, green tea catechins; HDAC, histone deacetylase; HGF, hepatocyte growth factor; HGPIN, high-grade prostatic intraepithelial neoplasia; HIF, hypoxia-inducible factor; HIF-1α, hypoxia-inducible factor 1 alpha; HIF-2α, hypoxia-inducible factor 2 alpha; HO-1, heme oxygenase 1; ICI(s), immune checkpoint inhibitor(s); IFN, interferon; IGF, insulin-like growth factor; IGF-1, insulin-like growth factor 1; IGF2, insulin-like growth factor 2; IL-1β, interleukin 1 beta; IL1R1, interleukin 1 receptor type 1; JAK, Janus kinase; JNK, c-Jun *N*-terminal kinase; KIT, KIT proto-oncogene receptor tyrosine kinase; KLK3, kallikrein-related peptidase 3; KRAS, KRAS proto-oncogene GTPase; LC3B-II, lipidated microtubule-associated protein 1 light chain 3 beta; MAPK, mitogen-activated protein kinase; MDM2, mouse double minute 2 homolog; MET/c-MET, MET proto-oncogene receptor tyrosine kinase; miRNA, microRNA; MMP, matrix metalloproteinase; MRP1, multidrug resistance-associated protein 1; mTOR, mechanistic target of rapamycin; mTORC1, mechanistic target of rapamycin complex 1; NF-κB, nuclear factor kappa B; NQO1, NAD(P)H quinone dehydrogenase 1; NRF2, nuclear factor erythroid 2-related factor 2; PARP, poly(ADP-ribose) polymerase; PCa, prostate cancer; PD-1, programmed cell death protein 1; PD-L1, programmed death-ligand 1; P-gp, PSA, prostate-specific antigen; PTEN, phosphatase and tensin homolog; QoL, quality of life; RB1, retinoblastoma 1; RCC, renal cell carcinoma; ROS, reactive oxygen species; RTK(s), receptor tyrosine kinase(s); STAT, signal transducer and activator of transcription; STAT3, signal transducer and activator of transcription 3; TGCT(s), testicular germ cell tumor(s); TKI(s), tyrosine kinase inhibitor(s); TME, tumor microenvironment; TP53, tumor protein p53; TRAMP, transgenic adenocarcinoma of the mouse prostate; Treg, regulatory T cell; TSC2, tuberous sclerosis complex 2; TUNEL, terminal deoxynucleotidyl transferase-mediated dUTP nick-end labeling; TURBT, transurethral resection of bladder tumor; UA, ursolic acid; UGT, UDP-glucuronosyltransferase; UMs, urologic malignancies; uPAR, urokinase-type plasminogen activator receptor; VEGF, vascular endothelial growth factor; VEGF-C/VEGFC, vascular endothelial growth factor C; VEGFR, vascular endothelial growth factor receptor; VEGFR2, vascular endothelial growth factor receptor 2.

## References

[1] Hushmandi K., Farahani N., Einollahi B., Salimimoghadam S., Alimohammadi M., Liang L., Liu L., Sethi G. (2025). Deciphering Molecular Pathways in Urological Cancers: A Gateway to Precision Therapeutics. J. Adv. Res..

[2] Tian Y.-Q., Yang J.-C., Hu J.-J., Ding R., Ye D.-W., Shang J.-W. (2023). Trends and Risk Factors of Global Incidence, Mortality, and Disability of Genitourinary Cancers from 1990 to 2019: Systematic Analysis for the Global Burden of Disease Study 2019. Front. Public Health.

[3] Glaviano A., Foo A.S.C., Lam H.Y., Yap K.C.H., Jacot W., Jones R.H., Eng H., Nair M.G., Makvandi P., Geoerger B. (2023). PI3K/AKT/mTOR Signaling Transduction Pathway and Targeted Therapies in Cancer. Mol. Cancer.

[4] Rezaei S., Nikpanjeh N., Rezaee A., Gholami S., Hashemipour R., Biavarz N., Yousefi F., Tashakori A., Salmani F., Rajabi R. (2023). PI3K/Akt Signaling in Urological Cancers: Tumorigenesis Function, Therapeutic Potential, and Therapy Response Regulation. Eur. J. Pharmacol..

[5] Shorning B.Y., Dass M.S., Smalley M.J., Pearson H.B. (2020). The PI3K-AKT-mTOR Pathway and Prostate Cancer: At the Crossroads of AR, MAPK, and WNT Signaling. Int. J. Mol. Sci..

[6] Tortorella E., Giantulli S., Sciarra A., Silvestri I. (2023). AR and PI3K/AKT in Prostate Cancer: A Tale of Two Interconnected Pathways. Int. J. Mol. Sci..

[7] Huang P., Wang J., Yu Z., Lu J., Sun Z., Chen Z. (2025). Redefining Bladder Cancer Treatment: Innovations in Overcoming Drug Resistance and Immune Evasion. Front. Immunol..

[8] Mazumder S., Higgins P.J., Samarakoon R. (2023). Downstream Targets of VHL/HIF-α Signaling in Renal Clear Cell Carcinoma Progression: Mechanisms and Therapeutic Relevance. Cancers.

[9] Guo Q., Jin Y., Chen X., Ye X., Shen X., Lin M., Zeng C., Zhou T., Zhang J. (2024). NF-κB in Biology and Targeted Therapy: New Insights and Translational Implications. Signal Transduct. Target. Ther..

[10] Mateo J., Boysen G., Barbieri C.E., Bryant H.E., Castro E., Nelson P.S., Olmos D., Pritchard C.C., Rubin M.A., de Bono J.S. (2017). DNA Repair in Prostate Cancer: Biology and Clinical Implications. Eur. Urol..

[11] Antar R.M., Fawaz C., Gonzalez D., Xu V.E., Drouaud A.P., Krastein J., Pio F., Murdock A., Youssef K., Sobol S. (2024). The Evolving Molecular Landscape and Actionable Alterations in Urologic Cancers. Curr. Oncol..

[12] Lozano R., Castro E., Aragón I.M., Cendón Y., Cattrini C., López-Casas P.P., Olmos D. (2021). Genetic Aberrations in DNA Repair Pathways: A Cornerstone of Precision Oncology in Prostate Cancer. Br. J. Cancer.

[13] Mirzaei S., Paskeh M.D.A., Okina E., Gholami M.H., Hushmandi K., Hashemi M., Kalu A., Zarrabi A., Nabavi N., Rabiee N. (2022). Molecular Landscape of LncRNAs in Prostate Cancer: A Focus on Pathways and Therapeutic Targets for Intervention. J. Exp. Clin. Cancer Res..

[14] Mitra A.P., Datar R.H., Cote R.J. (2006). Molecular Pathways in Invasive Bladder Cancer: New Insights into Mechanisms, Progression, and Target Identification. J. Clin. Oncol..

[15] Xue W., Zhao Y., Zhang G., Li Z., Li J., Fei X. (2025). The Role of m5C, m1A and m7G Modifications in Tumors of Urinary System. Front. Cell Dev. Biol..

[16] Abah M.O., Ogenyi D.O., Zhilenkova A.V., Essogmo F.E., Ngaha Tchawe Y.S., Uchendu I.K., Pascal A.M., Nikitina N.M., Rusanov A.S., Sanikovich V.D. (2024). Innovative Therapies Targeting Drug-Resistant Biomarkers in Metastatic Clear Cell Renal Cell Carcinoma (ccRCC). Int. J. Mol. Sci..

[17] Jiang A., Li J., He Z., Liu Y., Qiao K., Fang Y., Qu L., Luo P., Lin A., Wang L. (2024). Renal Cancer: Signaling Pathways and Advances in Targeted Therapies. MedComm.

[18] Lai Y., Zhao Z., Zeng T., Liang X., Chen D., Duan X., Zeng G., Wu W. (2018). Crosstalk between VEGFR and Other Receptor Tyrosine Kinases for TKI Therapy of Metastatic Renal Cell Carcinoma. Cancer Cell Int..

[19] Rassy E., Flippot R., Albiges L. (2020). Tyrosine Kinase Inhibitors and Immunotherapy Combinations in Renal Cell Carcinoma. Ther. Adv. Med. Oncol..

[20] Das M.K., Evensen H.S.F., Furu K., Haugen T.B. (2019). miRNA-302s May Act as Oncogenes in Human Testicular Germ Cell Tumours. Sci. Rep..

[21] Országhová Z., Kalavska K., Mego M., Chovanec M. (2022). Overcoming Chemotherapy Resistance in Germ Cell Tumors. Biomedicines.

[22] Voutsadakis I.A. (2014). The Chemosensitivity of Testicular Germ Cell Tumors. Cell. Oncol..

[23] Cháirez-Ramírez M.H., de la Cruz-López K.G., García-Carrancá A. (2021). Polyphenols as Antitumor Agents Targeting Key Players in Cancer-Driving Signaling Pathways. Front. Pharmacol..

[24] Logue J.S., Morrison D.K. (2012). Complexity in the Signaling Network: Insights from the Use of Targeted Inhibitors in Cancer Therapy. Genes. Dev..

[25] Bhatia M., Bhalerao M., Cruz-Martins N., Kumar D. (2021). Curcumin and Cancer Biology: Focusing Regulatory Effects in Different Signalling Pathways. Phytother. Res..

[26] Fontana F., Raimondi M., Marzagalli M., Di Domizio A., Limonta P. (2020). Natural Compounds in Prostate Cancer Prevention and Treatment: Mechanisms of Action and Molecular Targets. Cells.

[27] Hashemi M., Mirzaei S., Barati M., Hejazi E.S., Kakavand A., Entezari M., Salimimoghadam S., Kalbasi A., Rashidi M., Taheriazam A. (2022). Curcumin in the Treatment of Urological Cancers: Therapeutic Targets, Challenges and Prospects. Life Sci..

[28] Hedayati N., Safari M.H., Milasi Y.E., Kahkesh S., Farahani N., Khoshnazar S.M., Dorostgou Z., Alaei E., Alimohammadi M., Rahimzadeh P. (2025). Modulation of the PI3K/Akt Signaling Pathway by Resveratrol in Cancer: Molecular Mechanisms and Therapeutic Opportunity. Discov. Oncol..

[29] Rahman M.M., Ishtiaque G.M.A., Rahat S.A., Hossain M.A., Islam M.R., Maeesa S.K., Rifat K., Akash S., Begum R., Hari Chandana K. (2023). Multifunctional Role of Natural Products for Therapeutic Approaches of Prostate Cancer: An Updated Review. J. Herb. Med..

[30] Di Napoli R., Balzano N., Mascolo A., Cimmino C., Vitiello A., Zovi A., Capuano A., Boccellino M. (2023). What Is the Role of Nutraceutical Products in Cancer Patients? A Systematic Review of Randomized Clinical Trials. Nutrients.

[31] Bettuzzi S., Brausi M., Rizzi F., Castagnetti G., Peracchia G., Corti A. (2006). Chemoprevention of Human Prostate Cancer by Oral Administration of Green Tea Catechins in Volunteers with High-Grade Prostate Intraepithelial Neoplasia: A Preliminary Report from a One-Year Proof-of-Principle Study. Cancer Res..

[32] Kumar N.B., Pow-Sang J., Egan K.M., Spiess P.E., Dickinson S., Salup R., Helal M., McLarty J., Williams C.R., Schreiber F. (2015). Randomized, Placebo-Controlled Trial of Green Tea Catechins for Prostate Cancer Prevention. Cancer Prev. Res..

[33] Lane J.A., Er V., Avery K.N.L., Horwood J., Cantwell M., Caro G.P., Crozier A., Smith G.D., Donovan J.L., Down L. (2018). ProDiet: A Phase II Randomized Placebo-Controlled Trial of Green Tea Catechins and Lycopene in Men at Increased Risk of Prostate Cancer. Cancer Prev. Res..

[34] Gontero P., Marra G., Soria F., Oderda M., Zitella A., Baratta F., Chiorino G., Gregnanin I., Daniele L., Cattel L. (2015). A Randomized Double-Blind Placebo Controlled Phase I–II Study on Clinical and Molecular Effects of Dietary Supplements in Men with Precancerous Prostatic Lesions. Chemoprevention or “Chemopromotion”?. Prostate.

[35] Lilly M.B., Wu C., Ke Y., Chen W.-P., Soloff A.C., Armeson K., Yokoyama N.N., Li X., Song L., Yuan Y. (2024). A Phase I Study of Docetaxel plus Synthetic Lycopene in Metastatic Prostate Cancer Patients. Clin. Transl. Med..

[36] Liss M.A., Dursun F., Hackman G.L., Gadallah M.I., Saha A., Friedman C.A., Rathore A.S., Chandra P., White J.R., Tiziani S. (2024). Phase 1 Clinical Trial Evaluating Safety, Bioavailability, and Gut Microbiome with a Combination of Curcumin and Ursolic Acid in Lipid Enhanced Capsules. J. Tradit. Complement. Med..

[37] Kroon M.A.G.M., van Laarhoven H.W.M., Swart E.L., van Tellingen O., Kemper E.M. (2025). A Pharmacokinetic Study and Critical Reappraisal of Curcumin Formulations Enhancing Bioavailability. iScience.

[38] Panknin T.M., Howe C.L., Hauer M., Bucchireddigari B., Rossi A.M., Funk J.L. (2023). Curcumin Supplementation and Human Disease: A Scoping Review of Clinical Trials. Int. J. Mol. Sci..

[39] Porfyris O., Detopoulou P., Adamantidi T., Tsoupras A., Papageorgiou D., Ioannidis A., Rojas Gil A.P. (2025). Phytochemicals as Chemo-Preventive and Therapeutic Agents Against Bladder Cancer: A Comprehensive Review. Diseases.

[40] Xia Y., Chen R., Lu G., Li C., Lian S., Kang T.-W., Jung Y.D. (2021). Natural Phytochemicals in Bladder Cancer Prevention and Therapy. Front. Oncol..

[41] Maimon Y., Amiel G., Cohen Z., Hoffman A., Samuels N. (2024). Prevention of Bladder Cancer Recurrence with the Botanical Formula LCS103: A Case Series Study. Integr. Cancer Ther..

[42] Kornel A., Nadile M., Retsidou M.I., Sakellakis M., Gioti K., Beloukas A., Sze N.S.K., Klentrou P., Tsiani E. (2023). Ursolic Acid against Prostate and Urogenital Cancers: A Review of In Vitro and In Vivo Studies. Int. J. Mol. Sci..

[43] Li D., Wang J., Tuo Z., Yoo K.H., Yu Q., Miyamoto A., Zhang C., Ye X., Wei W., Wu R. (2024). Natural Products and Derivatives in Renal, Urothelial and Testicular Cancers: Targeting Signaling Pathways and Therapeutic Potential. Phytomedicine.

[44] Kumar N.B., Dickinson S.I., Schell M.J., Manley B.J., Poch M.A., Pow-Sang J. (2018). Green Tea Extract for Prevention of Prostate Cancer Progression in Patients on Active Surveillance. Oncotarget.

[45] Kumar N.B., Hogue S., Pow-Sang J., Poch M., Manley B.J., Li R., Dhillon J., Yu A., Byrd D.A. (2022). Effects of Green Tea Catechins on Prostate Cancer Chemoprevention: The Role of the Gut Microbiome. Cancers.

[46] Kumar N.B., Pow-Sang J., Spiess P.E., Park J., Salup R., Williams C.R., Parnes H., Schell M.J. (2016). Randomized, Placebo-Controlled Trial Evaluating the Safety of One-Year Administration of Green Tea Catechins. Oncotarget.

[47] Henning S.M., Aronson W.J., Wang P., Wang J., Lee R.-P., Grojean E.M., Ly A., Hsu M., Heber D., Li Z. (2017). Combination of Quercetin and Green Tea Extract Increased Plasma Epicatechingallate and Decreased Urine Epigallocatechin and Epicatechin Concentrations. FASEB J..

[48] Henning S.M., Wang P., Lee R.-P., Trang A., Husari G., Yang J., Grojean E.M., Ly A., Hsu M., Heber D. (2020). Prospective Randomized Trial Evaluating Blood and Prostate Tissue Concentrations of Green Tea Polyphenols and Quercetin in Men with Prostate Cancer. Food Funct..

[49] Hashemi M., Taheriazam A., Daneii P., Hassanpour A., Kakavand A., Rezaei S., Hejazi E.S., Aboutalebi M., Gholamrezaie H., Saebfar H. (2023). Targeting PI3K/Akt Signaling in Prostate Cancer Therapy. J. Cell Commun. Signal..

[50] Benjamin D.J., Mita A.C. (2025). FGFR-Altered Urothelial Carcinoma: Resistance Mechanisms and Therapeutic Strategies. Target. Oncol..

[51] (2014). Cancer Genome Atlas Research Netwok. Comprehensive Molecular Characterization of Urothelial Bladder Carcinoma. Nature.

[52] Quistini A., Chierigo F., Fallara G., Depalma M., Tozzi M., Maggi M., Jannello L.M.I., Pellegrino F., Mantica G., Terracciano D. (2025). Androgen Receptor Signalling in Prostate Cancer: Mechanisms of Resistance to Endocrine Therapies. Res. Rep. Urol..

[53] Raith F., O’Donovan D.H., Lemos C., Politz O., Haendler B. (2023). Addressing the Reciprocal Crosstalk between the AR and the PI3K/AKT/mTOR Signaling Pathways for Prostate Cancer Treatment. Int. J. Mol. Sci..

[54] Su P., Zhang M., Kang X. (2023). Targeting C-Met in the Treatment of Urologic Neoplasms: Current Status and Challenges. Front. Oncol..

[55] Antognelli C., Talesa V.N. (2018). Glyoxalases in Urological Malignancies. Int. J. Mol. Sci..

[56] Pelletier J., Graff J., Ruggero D., Sonenberg N. (2015). Targeting the eIF4F Translation Initiation Complex: A Critical Nexus for Cancer Development. Cancer Res..

[57] Krajewski W., Dzięgała M., Kołodziej A., Dembowski J., Zdrojowy R. (2016). Vitamin D and Urological Cancers. Cent. Eur. J. Urol..

[58] Bosland M.C., Kato I., Zeleniuch-Jacquotte A., Schmoll J., Enk Rueter E., Melamed J., Kong M.X., Macias V., Kajdacsy-Balla A., Lumey L.H. (2013). Effect of Soy Protein Isolate Supplementation on Biochemical Recurrence of Prostate Cancer after Radical Prostatectomy: A Randomized Trial. JAMA.

[59] deVere White R.W., Tsodikov A., Stapp E.C., Soares S.E., Fujii H., Hackman R.M. (2010). Effects of a High Dose, Aglycone-Rich Soy Extract on Prostate-Specific Antigen and Serum Isoflavone Concentrations in Men with Localized Prostate Cancer. Nutr. Cancer.

[60] Van der Eecken H., Joniau S., Berghen C., Rans K., De Meerleer G. (2023). The Use of Soy Isoflavones in the Treatment of Prostate Cancer: A Focus on the Cellular Effects. Nutrients.

[61] Kaufman-Szymczyk A., Jalmuzna J., Lubecka-Gajewska K. (2025). Soy-Derived Isoflavones as Chemo-Preventive Agents Targeting Multiple Signalling Pathways for Cancer Prevention and Therapy. Br. J. Pharmacol..

[62] Kim I.-S. (2021). Current Perspectives on the Beneficial Effects of Soybean Isoflavones and Their Metabolites for Humans. Antioxidants.

[63] Lazarevic B., Boezelijn G., Diep L.M., Kvernrod K., Ogren O., Ramberg H., Moen A., Wessel N., Berg R.E., Egge-Jacobsen W. (2011). Efficacy and Safety of Short-Term Genistein Intervention in Patients with Localized Prostate Cancer Prior to Radical Prostatectomy: A Randomized, Placebo-Controlled, Double-Blind Phase 2 Clinical Trial. Nutr. Cancer.

[64] Lu Z., Zhou R., Kong Y., Wang J., Xia W., Guo J., Liu J., Sun H., Liu K., Yang J. (2016). S-Equol, a Secondary Metabolite of Natural Anticancer Isoflavone Daidzein, Inhibits Prostate Cancer Growth In Vitro and In Vivo, Though Activating the Akt/FOXO3a Pathway. Curr. Cancer Drug Targets.

[65] Napora J.K., Short R.G., Muller D.C., Carlson O.D., Odetunde J.O., Xu X., Carducci M., Travison T.G., Maggio M., Egan J.M. (2011). High-Dose Isoflavones Do Not Improve Metabolic and Inflammatory Parameters in Androgen-Deprived Men with Prostate Cancer. J. Androl..

[66] Ul Hassan M.H., Shahbaz M., Imran M., Momal U., Naeem H., Mujtaba A., Hussain M., Anwar M.J., Alsagaby S.A., Al Abdulmonem W. (2025). Isoflavones: Promising Natural Agent for Cancer Prevention and Treatment. Food Sci. Nutr..

[67] Wang S., DeGroff V.L., Clinton S.K. (2003). Tomato and Soy Polyphenols Reduce Insulin-like Growth Factor-I-Stimulated Rat Prostate Cancer Cell Proliferation and Apoptotic Resistance In Vitro via Inhibition of Intracellular Signaling Pathways Involving Tyrosine Kinase. J. Nutr..

[68] Adhami V.M., Siddiqui I.A., Sarfaraz S., Khwaja S.I., Hafeez B.B., Ahmad N., Mukhtar H. (2009). Effective Prostate Cancer Chemopreventive Intervention with Green Tea Polyphenols in the TRAMP Model Depends on the Stage of the Disease. Clin. Cancer Res..

[69] Deb G., Shankar E., Thakur V.S., Ponsky L.E., Bodner D.R., Fu P., Gupta S. (2019). Green Tea–Induced Epigenetic Reactivation of Tissue Inhibitor of Matrix Metalloproteinase-3 Suppresses Prostate Cancer Progression through Histone-Modifying Enzymes. Mol. Carcinog..

[70] Henning S.M., Wang P., Heber D. (2011). Chemopreventive Effects of Tea in Prostate Cancer: Green Tea versus Black Tea. Mol. Nutr. Food Res..

[71] Henning S.M., Wang P., Said J.W., Huang M., Grogan T., Elashoff D., Carpenter C.L., Heber D., Aronson W.J. (2015). Randomized Clinical Trial of Brewed Green and Black Tea in Men with Prostate Cancer Prior to Prostatectomy. Prostate.

[72] Lee S.-C., Chan W.-K., Lee T.-W., Lam W.-H., Wang X., Chan T.-H., Wong Y.-C. (2008). Effect of a Prodrug of the Green Tea Polyphenol (-)-Epigallocatechin-3-Gallate on the Growth of Androgen-Independent Prostate Cancer in Vivo. Nutr. Cancer.

[73] Moalemi S.F.S.S., Safari F., Ahvati H. (2025). Suppression of Cellular Proliferation in PC3 Prostate Cancer Cells by Green Tea Extract Through Induction of miR-34a Expression. Food Sci. Nutr..

[74] Shenoy A.G., Ravi V., Vishwakarma R., Varghese S., Subair S., Vaswani R., Raju R., Revikumar A., Rehman N. (2025). Prostate Cancer and Tea: CYP17A1 Inhibition by Phytochemicals from Tea Plant *Camellia sinensis* L. and Implications for Anti-Androgenic Effect. OMICS.

[75] Siddiqui I.A., Asim M., Hafeez B.B., Adhami V.M., Tarapore R.S., Mukhtar H. (2011). Green Tea Polyphenol EGCG Blunts Androgen Receptor Function in Prostate Cancer. FASEB J..

[76] Wang P., Aronson W.J., Huang M., Zhang Y., Lee R.-P., Heber D., Henning S.M. (2010). Green Tea Polyphenols and Metabolites in Prostatectomy Tissue: Implications for Cancer Prevention. Cancer Prev. Res..

[77] Holzapfel N.P., Holzapfel B.M., Champ S., Feldthusen J., Clements J., Hutmacher D.W. (2013). The Potential Role of Lycopene for the Prevention and Therapy of Prostate Cancer: From Molecular Mechanisms to Clinical Evidence. Int. J. Mol. Sci..

[78] Jiang L.-N., Liu Y.-B., Li B.-H. (2019). Lycopene Exerts Anti-Inflammatory Effect to Inhibit Prostate Cancer Progression. Asian J. Androl..

[79] Kapała A., Szlendak M., Motacka E. (2022). The Anti-Cancer Activity of Lycopene: A Systematic Review of Human and Animal Studies. Nutrients.

[80] López-Solís R., Castro-Barquero S., Donat-Vargas C., Corrado M., Arancibia-Riveros C., Martínez-González M.Á., Salas-Salvadó J., Sorlí J.V., Serra-Majem L., Fitó M. (2025). Lycopene Intake and Prostate Cancer Risk in Men at High Cardiovascular Risk: A Prospective Cohort Study. BMC Med..

[81] Maaz M., Sultan M.T., Khalid M.U., Raza H., Imran M., Hussain M., Al Abdulmonem W., Alsagaby S.A., Abdelgawad M.A., Ghoneim M.M. (2025). A Comprehensive Review on the Molecular Mechanism of Lycopene in Cancer Therapy. Food Sci. Nutr..

[82] Mirahmadi M., Azimi-Hashemi S., Saburi E., Kamali H., Pishbin M., Hadizadeh F. (2020). Potential Inhibitory Effect of Lycopene on Prostate Cancer. Biomed. Pharmacother..

[83] Moran N.E., Alexander B., Garg S., Marchant N., Hason N.A. (2024). Relative Uptake of Tomato Carotenoids by In Vitro Intestinal and Prostate Cancer Cells. J. Nutr..

[84] Abd Wahab N.A., Lajis N.H., Abas F., Othman I., Naidu R. (2020). Mechanism of Anti-Cancer Activity of Curcumin on Androgen-Dependent and Androgen-Independent Prostate Cancer. Nutrients.

[85] Ali S.A., Helmy H.I., Gaber M.H. (2026). Comparative In Vitro Cytotoxicity of Free Curcumin and a Liposomal Curcumin Formulation on Various Human Cancer Cell Lines. Sci. Rep..

[86] Clark R., Saha A., Lavender Hackman G., Friedman C.A., Gorgoglione R., Tiziani S., DiGiovanni J. (2026). A Combination of Xanthohumol and Ursolic Acid in the Diet Leads to Synergistic Inhibition of Prostate Cancer Progression. Mol. Carcinog..

[87] Esmaeli M., Dehghanpour Dehabadi M. (2025). Curcumin in Prostate Cancer: A Systematic Review of Molecular Mechanisms and Nanoformulated Therapeutic Strategies. BMC Cancer.

[88] Friedman C.A., Saha A., Clark R., Wilder C., Wright J., DiGiovanni J. (2025). Synergistic Inhibition of Prostate Cancer Progression in Mice with a Combination of Curcumin and Ursolic Acid in the Diet. Mol. Carcinog..

[89] Hussain Y., Mirzaei S., Ashrafizadeh M., Zarrabi A., Hushmandi K., Khan H., Daglia M. (2021). Quercetin and Its Nano-Scale Delivery Systems in Prostate Cancer Therapy: Paving the Way for Cancer Elimination and Reversing Chemoresistance. Cancers.

[90] Katta S., Srivastava A., Thangapazham R.L., Rosner I.L., Cullen J., Li H., Sharad S. (2019). Curcumin-Gene Expression Response in Hormone Dependent and Independent Metastatic Prostate Cancer Cells. Int. J. Mol. Sci..

[91] Khan N., Asim M., Afaq F., Zaid M.A., Mukhtar H. (2008). A Novel Dietary Flavonoid Fisetin Inhibits Androgen Receptor Signaling and Tumor Growth in Athymic Nude Mice. Cancer Res..

[92] Li J., Wang X., Xue L., He Q. (2024). Exploring the Therapeutic Mechanism of Curcumin in Prostate Cancer Using Network Pharmacology and Molecular Docking. Heliyon.

[93] Meng Y., Lin Z.-M., Ge N., Zhang D.-L., Huang J., Kong F. (2015). Ursolic Acid Induces Apoptosis of Prostate Cancer Cells via the PI3K/Akt/mTOR Pathway. Am. J. Chin. Med..

[94] Shanmugam M.K., Rajendran P., Li F., Nema T., Vali S., Abbasi T., Kapoor S., Sharma A., Kumar A.P., Ho P.C. (2011). Ursolic Acid Inhibits Multiple Cell Survival Pathways Leading to Suppression of Growth of Prostate Cancer Xenograft in Nude Mice. J. Mol. Med..

[95] Tavakoli Z., Khajeh K., Ranjbar B. (2026). A Hybrid Nanosystem for Prostate Cancer Therapy: Codelivery of Enzalutamide and Curcumin via Selenium-Embedded Mesoporous Silica and Chitosan Nanoparticles. ChemistryOpen.

[96] Termini D., Den Hartogh D.J., Jaglanian A., Tsiani E. (2020). Curcumin against Prostate Cancer: Current Evidence. Biomolecules.

[97] Wang Y., Zhao X., Zhao J., Wang X., Guo Y., Chen S., Jin Y., Peng P., Zhang W. (2025). ETS-Related Gene as a Key Factor in Curcumin Inhibition of Glucose Metabolism in Prostate Cancer Cells: An In Vitro Experimental Study. J. Yeungnam Med. Sci..

[98] Ye M., Tian H., Lin S., Mo J., Li Z., Chen X., Liu J. (2020). Resveratrol Inhibits Proliferation and Promotes Apoptosis via the Androgen Receptor Splicing Variant 7 and PI3K/AKT Signaling Pathway in LNCaP Prostate Cancer Cells. Oncol. Lett..

[99] Kareem Al-Hetty H.R.A., Ahmed A.T., Saleem H.M., Abdulhadi H.L., Muhammed T.M., Ali L.H. (2024). Cellular and Molecular Mechanisms of Action of Epigallocatechin Gallate on Bladder Cancer: A Comprehensive Systematic Review. PharmaNutrition.

[100] Luo K.-W., Lung W.-Y., Chun-Xie, Luo X.-L., Huang W.-R. (2018). EGCG Inhibited Bladder Cancer T24 and 5637 Cell Proliferation and Migration via PI3K/AKT Pathway. Oncotarget.

[101] Sah D.K., Khoi P.N., Li S., Arjunan A., Jeong J.-U., Jung Y.D. (2022). (-)-Epigallocatechin-3-Gallate Prevents IL-1β-Induced uPAR Expression and Invasiveness via the Suppression of NF-κB and AP-1 in Human Bladder Cancer Cells. Int. J. Mol. Sci..

[102] Sun X., Song J., Li E., Geng H., Li Y., Yu D., Zhong C. (2019). (-)-Epigallocatechin-3-gallate Inhibits Bladder Cancer Stem Cells via Suppression of Sonic Hedgehog Pathway. Oncol. Rep..

[103] Yin Z., Li J., Kang L., Liu X., Luo J., Zhang L., Li Y., Cai J. (2021). Epigallocatechin-3-Gallate Induces Autophagy-Related Apoptosis Associated with LC3B II and Beclin Expression of Bladder Cancer Cells. J. Food Biochem..

[104] Chen L., Cao H., Huang Q., Xiao J., Teng H. (2022). Absorption, Metabolism and Bioavailability of Flavonoids: A Review. Crit. Rev. Food Sci. Nutr..

[105] Crocetto F., di Zazzo E., Buonerba C., Aveta A., Pandolfo S.D., Barone B., Trama F., Caputo V.F., Scafuri L., Ferro M. (2021). Kaempferol, Myricetin and Fisetin in Prostate and Bladder Cancer: A Systematic Review of the Literature. Nutrients.

[106] de Morais E.F., de Oliveira L.Q.R., de Farias Morais H.G., de Souto Medeiros M.R., Freitas R.d.A., Rodini C.O., Coletta R.D. (2024). The Anticancer Potential of Kaempferol: A Systematic Review Based on In Vitro Studies. Cancers.

[107] Felice M.R., Maugeri A., De Sarro G., Navarra M., Barreca D. (2022). Molecular Pathways Involved in the Anti-Cancer Activity of Flavonols: A Focus on Myricetin and Kaempferol. Int. J. Mol. Sci..

[108] He Y., Wu X., Cao Y., Hou Y., Chen H., Wu L., Lu L., Zhu W., Gu Y. (2016). Daidzein Exerts Anti-Tumor Activity against Bladder Cancer Cells via Inhibition of FGFR3 Pathway. Neoplasma.

[109] Li R., Linscott J., Catto J.W.F., Daneshmand S., Faltas B.M., Kamat A.M., Meeks J.J., Necchi A., Pradere B., Ross J.S. (2025). FGFR Inhibition in Urothelial Carcinoma. Eur. Urol..

[110] Messing E., Gee J.R., Saltzstein D.R., Kim K., diSant’Agnese A., Kolesar J., Harris L., Faerber A., Havighurst T., Young J.M. (2012). A Phase 2 Cancer Chemoprevention Biomarker Trial of Isoflavone G-2535 (Genistein) in Presurgical Bladder Cancer Patients. Cancer Prev. Res..

[111] Murota K., Nakamura Y., Uehara M. (2018). Flavonoid Metabolism: The Interaction of Metabolites and Gut Microbiota. Biosci. Biotechnol. Biochem..

[112] Williamson G., Kay C.D., Crozier A. (2018). The Bioavailability, Transport, and Bioactivity of Dietary Flavonoids: A Review from a Historical Perspective. Compr. Rev. Food Sci. Food Saf..

[113] Gai L., Cai N., Wang L., Xu X., Kong X. (2013). Ursolic Acid Induces Apoptosis via Akt/NF-κB Signaling Suppression in T24 Human Bladder Cancer Cells. Mol. Med. Rep..

[114] Huang X., Sun Y., Tong H., Zhu J., Pan J., Wen P., He W. (2025). Ursolic Acid Sensitizes Bladder Cancer to Gemcitabine Chemotherapy by Concurrently Targeting PI3K/AKT and JNK Pathways. Transl. Androl. Urol..

[115] Huang X.-L., Sun Y., Wen P., Pan J.-C., He W.-Y. (2024). The Potential Mechanism of Ursolic Acid in the Treatment of Bladder Cancer Based on Network Pharmacology and Molecular Docking. J. Int. Med. Res..

[116] Kamat A.M., Sethi G., Aggarwal B.B. (2007). Curcumin Potentiates the Apoptotic Effects of Chemotherapeutic Agents and Cytokines through Down-Regulation of Nuclear Factor-kappaB and Nuclear Factor-kappaB-Regulated Gene Products in IFN-Alpha-Sensitive and IFN-Alpha-Resistant Human Bladder Cancer Cells. Mol. Cancer Ther..

[117] Limami Y., Pinon A., Wahnou H., Oudghiri M., Liagre B., Simon A., Duval R.E. (2023). Ursolic Acid’s Alluring Journey: One Triterpenoid vs. Cancer Hallmarks. Molecules.

[118] Silva L.W.d.P.E., Almeida T.C., Teixeira M.S.d.S., Cerrutti C.M.V., Agostini L.d.C., Brandão G.C., da Silva G.N. (2025). Antiproliferative Effects of the Triterpene Ursolic Acid Natural Product in Bladder and Ovarian Tumor Cell Lines. Drug Dev. Res..

[119] Sun X., Deng Q.-F., Liang Z.-F., Zhang Z.-Q., Zhao L., Geng H., Xie D.-D., Wang Y., Yu D.-X., Zhong C.-Y. (2016). Curcumin Reverses Benzidine-Induced Cell Proliferation by Suppressing ERK1/2 Pathway in Human Bladder Cancer T24 Cells. Exp. Toxicol. Pathol..

[120] Tian B., Wang Z., Zhao Y., Wang D., Li Y., Ma L., Li X., Li J., Xiao N., Tian J. (2008). Effects of Curcumin on Bladder Cancer Cells and Development of Urothelial Tumors in a Rat Bladder Carcinogenesis Model. Cancer Lett..

[121] Tian B., Zhao Y., Liang T., Ye X., Li Z., Yan D., Fu Q., Li Y. (2017). Curcumin Inhibits Urothelial Tumor Development by Suppressing IGF2 and IGF2-Mediated PI3K/AKT/mTOR Signaling Pathway. J. Drug Target..

[122] Wang J., Wang Z., Wang H., Zhao J., Zhang Z. (2011). Curcumin Induces Apoptosis in EJ Bladder Cancer Cells via Modulating C-Myc and PI3K/Akt Signaling Pathway. World J. Oncol..

[123] Zafar S., Khan K., Hafeez A., Irfan M., Armaghan M., Rahman A.U., Gürer E.S., Sharifi-Rad J., Butnariu M., Bagiu I.-C. (2022). Ursolic Acid: A Natural Modulator of Signaling Networks in Different Cancers. Cancer Cell Int..

[124] Zheng Q., Jin F., Yao C., Zhang T., Zhang G., Ai X. (2012). Ursolic Acid-Induced AMP-Activated Protein Kinase (AMPK) Activation Contributes to Growth Inhibition and Apoptosis in Human Bladder Cancer T24 Cells. Biochem. Biophys. Res. Commun..

[125] Zoi V., Kyritsis A.P., Galani V., Lazari D., Sioka C., Voulgaris S., Alexiou G.A. (2024). The Role of Curcumin in Cancer: A Focus on the PI3K/Akt Pathway. Cancers.

[126] Buonerba C., De Placido P., Bruzzese D., Pagliuca M., Ungaro P., Bosso D., Ribera D., Iaccarino S., Scafuri L., Liotti A. (2018). Isoquercetin as an Adjunct Therapy in Patients With Kidney Cancer Receiving First-Line Sunitinib (QUASAR): Results of a Phase I Trial. Front. Pharmacol..

[127] Ho W.J., Simon M.S., Yildiz V.O., Shikany J.M., Kato I., Beebe-Dimmer J.L., Cetnar J.P., Bock C.H. (2015). Antioxidant Micronutrients and the Risk of Renal Cell Carcinoma in the Women’s Health Initiative Cohort. Cancer.

[128] Hu J., La Vecchia C., Negri E., DesMeules M., Mery L. (2009). Canadian Cancer Registries Epidemiology Research Group Dietary Vitamin C, E, and Carotenoid Intake and Risk of Renal Cell Carcinoma. Cancer Causes Control..

[129] Huang J., Wang D., Xu C., Wu J. (2026). Integrated Network Pharmacology, Molecular Docking and Experimental Validation Reveal That Quercetin Suppresses Clear Cell Renal Cell Carcinoma via MMP9-Associated Macrophage Polarization. Biomedicines.

[130] Liu L., Barber E., Kellow N.J., Williamson G. (2025). Improving Quercetin Bioavailability: A Systematic Review and Meta-Analysis of Human Intervention Studies. Food Chem..

[131] Mohammadipoor N., Naiebi R., Mazhari S.A., Amooei F., Owrang M., Dastghaib S., Shams M., Maleki M.H., Dastghaib S. (2024). Improved Therapy for Clear Cell Renal Cell Carcinoma: Beta-Hydroxybutyrate and Quercetin Target Hypoxia-Induced Angiogenesis and Multidrug Resistance. Mol. Biol. Rep..

[132] Sałek-Zań A., Püsküllüoğlu M., Syrek-Kaplita K., Banaś T. (2025). Food Interactions with Tyrosine Kinase Inhibitors Used to Treat Advanced Renal Cell Carcinoma. Contemp. Oncol..

[133] Taheri D., Ghajar H.A., Mirzaei A., Mashhadi R., Dougaheh S.N.H., Bahri R.A., Khoshchehreh M., Tavoosian A., Aghamir S.M.K. (2024). Resveratrol Enhances Sensitivity of Renal Cell Carcinoma to Tivozanib: An In-Vitro Study. Tissue Cell..

[134] Zhu X.-W., Dong F.-M., Liu J., Li M.-S. (2022). Resveratrol Nanoparticles Suppresses Migration and Invasion of Renal Cell Carcinoma Cells by Inhibiting Matrix Metalloproteinase 2 Expression and Extracellular Signal-Regulated Kinase Pathway. J. Biomed. Nanotechnol..

[135] Ahmad I., Hoque M., Alam S.S.M., Zughaibi T.A., Tabrez S. (2023). Curcumin and Plumbagin Synergistically Target the PI3K/Akt/mTOR Pathway: A Prospective Role in Cancer Treatment. Int. J. Mol. Sci..

[136] Dai W., Ruan C., Zhang Y., Wang J., Han J., Shao Z., Sun Y., Liang J. (2020). Bioavailability Enhancement of EGCG by Structural Modification and Nano-Delivery: A Review. J. Funct. Foods.

[137] Gong X., Jiang L., Li W., Liang Q., Li Z. (2021). Curcumin Induces Apoptosis and Autophagy Inhuman Renal Cell Carcinoma Cells via Akt/mTOR Suppression. Bioengineered.

[138] Jayab N.A., Abed A., Talaat I.M., Hamoudi R. (2025). The Molecular Mechanism of NF-κB Dysregulation across Different Subtypes of Renal Cell Carcinoma. J. Adv. Res..

[139] Vieira I.R.S., Tessaro L., Lima A.K.O., Velloso I.P.S., Conte-Junior C.A. (2023). Recent Progress in Nanotechnology Improving the Therapeutic Potential of Polyphenols for Cancer. Nutrients.

[140] Mehmood S., Maqsood M., Mahtab N., Khan M.I., Sahar A., Zaib S., Gul S. (2022). Epigallocatechin Gallate: Phytochemistry, Bioavailability, Utilization Challenges, and Strategies. J. Food Biochem..

[141] Beier V., Wink M., Samstag Y. (2026). Plant-Derived Immunomodulators in Cancer: Balancing Immune Activation and Suppression within the Tumor Microenvironment. Adv. Biol. Regul..

[142] Lee J., Han Y., Wang W., Jo H., Kim H., Kim S., Yang K.-M., Kim S.-J., Dhanasekaran D.N., Song Y.S. (2021). Phytochemicals in Cancer Immune Checkpoint Inhibitor Therapy. Biomolecules.

[143] Rysz J., Ławiński J., Franczyk B., Gluba-Sagr A. (2025). Immune Checkpoint Inhibitors in Clear Cell Renal Cell Carcinoma (ccRCC). Int. J. Mol. Sci..

[144] Wu S., Oceguera Nava E.I., Ashong D., Chen G., Chen Q.-H. (2025). Natural Products Targeting the Androgen Receptor Signaling Pathway: Therapeutic Potential and Mechanisms. Curr. Issues Mol. Biol..

[145] Pratama F., Novitasari D., Mardianingrum R., Holik H.A., Ikram N.K.K., Muchtaridi M. (2026). Bioactive Natural Products Targeting Androgen Receptor Signaling in Prostate Cancer: A Systematic Review. Cancers.

[146] Tyagi A., Chandrasekaran B., Shukla V., Tyagi N., Sharma A.K., Damodaran C. (2024). Nutraceuticals Target Androgen Receptor-Splice Variants (AR-SV) to Manage Castration Resistant Prostate Cancer (CRPC). Pharmacol. Ther..

[147] Kinkade C.W., Castillo-Martin M., Puzio-Kuter A., Yan J., Foster T.H., Gao H., Sun Y., Ouyang X., Gerald W.L., Cordon-Cardo C. (2008). Targeting AKT/mTOR and ERK MAPK Signaling Inhibits Hormone-Refractory Prostate Cancer in a Preclinical Mouse Model. J. Clin. Investig..

[148] Besasie B.D., Saha A., DiGiovanni J., Liss M.A. (2024). Effects of Curcumin and Ursolic Acid in Prostate Cancer: A Systematic Review. Urologia.

[149] Chopra H., Bibi S., Goyal R., Gautam R.K., Trivedi R., Upadhyay T.K., Mujahid M.H., Shah M.A., Haris M., Khot K.B. (2022). Chemopreventive Potential of Dietary Nanonutraceuticals for Prostate Cancer: An Extensive Review. Front. Oncol..

[150] Kallifatidis G., Hoy J.J., Lokeshwar B.L. (2016). Bioactive Natural Products for Chemoprevention and Treatment of Castration-Resistant Prostate Cancer. Semin. Cancer Biol..

[151] Aggarwal R., Rydzewski N.R., Zhang L., Foye A., Kim W., Helzer K.T., Bakhtiar H., Chang S.L., Perry M.D., Gleave M. (2021). Prognosis Associated with Luminal and Basal Subtypes of Metastatic Prostate Cancer. JAMA Oncol..

[152] Zhao S.G., Chang S.L., Erho N., Yu M., Lehrer J., Alshalalfa M., Speers C., Cooperberg M.R., Kim W., Ryan C.J. (2017). Associations of Luminal and Basal Subtyping of Prostate Cancer with Prognosis and Response to Androgen Deprivation Therapy. JAMA Oncol..

[153] Dong B., Miao J., Wang Y., Luo W., Ji Z., Lai H., Zhang M., Cheng X., Wang J., Fang Y. (2020). Single-Cell Analysis Supports a Luminal-Neuroendocrine Transdifferentiation in Human Prostate Cancer. Commun. Biol..

[154] Favari C., Rinaldi de Alvarenga J.F., Sánchez-Martínez L., Tosi N., Mignogna C., Cremonini E., Manach C., Bresciani L., Del Rio D., Mena P. (2024). Factors Driving the Inter-Individual Variability in the Metabolism and Bioavailability of (Poly)Phenolic Metabolites: A Systematic Review of Human Studies. Redox Biol..

[155] Crocetto F., Boccellino M., Barone B., Di Zazzo E., Sciarra A., Galasso G., Settembre G., Quagliuolo L., Imbimbo C., Boffo S. (2020). The Crosstalk between Prostate Cancer and Microbiota Inflammation: Nutraceutical Products Are Useful to Balance This Interplay?. Nutrients.

[156] Yoshikata R., Myint K.Z.Y., Taguchi J. (2024). Comparison of Blood and Urine Concentrations of Equol by LC–MS/MS Method and Factors Associated with Equol Production in 466 Japanese Men and Women. PLoS ONE.

[157] Akaza H., Miyanaga N., Takashima N., Naito S., Hirao Y., Tsukamoto T., Fujioka T., Mori M., Kim W.-J., Song J.M. (2004). Comparisons of Percent Equol Producers between Prostate Cancer Patients and Controls: Case-Controlled Studies of Isoflavones in Japanese, Korean and American Residents. Jpn. J. Clin. Oncol..

[158] Itsumi M., Shiota M., Takeuchi A., Kashiwagi E., Inokuchi J., Tatsugami K., Kajioka S., Uchiumi T., Naito S., Eto M. (2016). Equol Inhibits Prostate Cancer Growth through Degradation of Androgen Receptor by S-Phase Kinase-Associated Protein 2. Cancer Sci..

[159] Miyanaga N., Akaza H., Hinotsu S., Fujioka T., Naito S., Namiki M., Takahashi S., Hirao Y., Horie S., Tsukamoto T. (2012). Prostate Cancer Chemoprevention Study: An Investigative Randomized Control Study Using Purified Isoflavones in Men with Rising Prostate-Specific Antigen. Cancer Sci..

[160] Luca S.V., Macovei I., Bujor A., Miron A., Skalicka-Woźniak K., Aprotosoaie A.C., Trifan A. (2020). Bioactivity of Dietary Polyphenols: The Role of Metabolites. Crit. Rev. Food Sci. Nutr..

[161] Williamson G. (2025). Bioavailability of Food Polyphenols: Current State of Knowledge. Annu. Rev. Food Sci. Technol..

[162] Wang Y., Romigh T., He X., Orloff M.S., Silverman R.H., Heston W.D., Eng C. (2010). Resveratrol Regulates the PTEN/AKT Pathway through Androgen Receptor-Dependent and -Independent Mechanisms in Prostate Cancer Cell Lines. Hum. Mol. Genet..

[163] Cai H., Scott E.N., Britton R.G., Parrott E., Ognibene T.J., Malfatti M., Khan M., Steward W.P., Brown K. (2021). Distribution and Metabolism of [14C]-Resveratrol in Human Prostate Tissue after Oral Administration of a “Dietary-Achievable” or “Pharmacological” Dose: What Are the Implications for Anticancer Activity?. Am. J. Clin. Nutr..

[164] Liu H., Chang G., Wang W., Ji Z., Cui J., Peng Y. (2022). Pharmacokinetics, Prostate Distribution and Metabolic Characteristics of Four Representative Flavones after Oral Administration of the Aerial Part of Glycyrrhiza Uralensis in Rats. Molecules.

[165] Seeram N.P., Aronson W.J., Zhang Y., Henning S.M., Moro A., Lee R.-P., Sartippour M., Harris D.M., Rettig M., Suchard M.A. (2007). Pomegranate Ellagitannin-Derived Metabolites Inhibit Prostate Cancer Growth and Localize to the Mouse Prostate Gland. J. Agric. Food Chem..

[166] Consoli V., D’Amico A.G., Russo C., Foderà E., Passarella D., Pecorino A., D’Agata V., Vanella L., Sorrenti V. (2026). Preclinical Evaluation of Waste-Derived Pomegranate Extract (PWE) as a Potential Preventing and Therapeutic Agent for Benign Prostatic Hyperplasia. Front. Mol. Biosci..

[167] Kobayashi E.H., Suzuki T., Funayama R., Nagashima T., Hayashi M., Sekine H., Tanaka N., Moriguchi T., Motohashi H., Nakayama K. (2016). Nrf2 Suppresses Macrophage Inflammatory Response by Blocking Proinflammatory Cytokine Transcription. Nat. Commun..

[168] Liu G.-H., Qu J., Shen X. (2008). NF-kappaB/P65 Antagonizes Nrf2-ARE Pathway by Depriving CBP from Nrf2 and Facilitating Recruitment of HDAC3 to MafK. Biochim. Biophys. Acta.

[169] DeNicola G.M., Karreth F.A., Humpton T.J., Gopinathan A., Wei C., Frese K., Mangal D., Yu K.H., Yeo C.J., Calhoun E.S. (2011). Oncogene-Induced Nrf2 Transcription Promotes ROS Detoxification and Tumorigenesis. Nature.

[170] Lin L., Wu Q., Lu F., Lei J., Zhou Y., Liu Y., Zhu N., Yu Y., Ning Z., She T. (2023). Nrf2 Signaling Pathway: Current Status and Potential Therapeutic Targetable Role in Human Cancers. Front. Oncol..

[171] Chestnut C., Subramaniam D., Dandawate P., Padhye S., Taylor J., Weir S., Anant S. (2021). Targeting Major Signaling Pathways of Bladder Cancer with Phytochemicals: A Review. Nutr. Cancer.

[172] Pereira F., Domingues M.R., Vitorino R., Guerra I.M.S., Santos L.L., Ferreira J.A., Ferreira R. (2024). Unmasking the Metabolite Signature of Bladder Cancer: A Systematic Review. Int. J. Mol. Sci..

[173] Xiao J.-F., Caliri A.W., Duex J.E., Theodorescu D. (2021). Targetable Pathways in Advanced Bladder Cancer: FGFR Signaling. Cancers.

[174] Meng L., Khorasanchi A., Jain R. (2025). Advancing Bladder Cancer Management: The Role of Neoadjuvant and Adjuvant Therapies and Biomarkers in Muscle Invasive Bladder Cancer. Curr. Treat. Options Oncol..

[175] Yang Y., Jain R.K., Gupta S., Tsung I., Gopalakrishnan D., Gheeya J.S., Wei L., Satturwar S., Parwani A., Collier K.A. (2025). A Phase 2 Trial of Neoadjuvant Futibatinib plus Durvalumab for Cisplatin-Ineligible Patients with *FGFR* Overexpressing Muscle-Invasive Bladder Cancer. JCO.

[176] Zhang R., Zang J., Jin D., Xie F., Shahatiaili A., Wu G., Zhang L., Wang L., Zhang Y., Zhao Z. (2023). Urinary Tumor DNA MRD Analysis to Identify Responders to Neoadjuvant Immunotherapy in Muscle-Invasive Bladder Cancer. Clin. Cancer Res..

[177] Kamoun A., de Reyniès A., Allory Y., Sjödahl G., Robertson A.G., Seiler R., Hoadley K.A., Groeneveld C.S., Al-Ahmadie H., Choi W. (2020). A Consensus Molecular Classification of Muscle-Invasive Bladder Cancer. Eur. Urol..

[178] McConkey D.J., Choi W. (2018). Molecular Subtypes of Bladder Cancer. Curr. Oncol. Rep..

[179] Lopez-Beltran A., Blanca A., Downes M.R., Cimadamore A., Montironi R., Cheng L. (2026). Molecular Pathology of Bladder Cancer. Histopathology.

[180] Peng Y., Song Y., Qin C., Ding M., Huang Z., Wang F., HuangFu Y., Yu L., Du Y., Xu T. (2025). Genomic Subtypes of Non-Muscle-Invasive Bladder Cancer: Guiding Immunotherapy Decision-Making for Patients Exposed to Aristolochic Acid. Mol. Med..

[181] Ascione C.M., Napolitano F., Esposito D., Servetto A., Belli S., Santaniello A., Scagliarini S., Crocetto F., Bianco R., Formisano L. (2023). Role of FGFR3 in Bladder Cancer: Treatment Landscape and Future Challenges. Cancer Treat. Rev..

[182] Noeraparast M., Krajina K., Pichler R., Niedersüß-Beke D., Shariat S.F., Grünwald V., Ahyai S., Pichler M. (2024). FGFR3 Alterations in Bladder Cancer: Sensitivity and Resistance to Targeted Therapies. Cancer Commun..

[183] Iida K., Naiki T., Naiki-Ito A., Suzuki S., Kato H., Nozaki S., Nagai T., Etani T., Nagayasu Y., Ando R. (2020). Luteolin Suppresses Bladder Cancer Growth via Regulation of Mechanistic Target of Rapamycin Pathway. Cancer Sci..

[184] Hayden A., Douglas J., Sommerlad M., Andrews L., Gould K., Hussain S., Thomas G.J., Packham G., Crabb S.J. (2014). The Nrf2 Transcription Factor Contributes to Resistance to Cisplatin in Bladder Cancer. Urol. Oncol..

[185] Khan K., Quispe C., Javed Z., Iqbal M.J., Sadia H., Raza S., Irshad A., Salehi B., Reiner Ž., Sharifi-Rad J. (2020). Resveratrol, Curcumin, Paclitaxel and miRNAs Mediated Regulation of PI3K/Akt/mTOR Pathway: Go Four Better to Treat Bladder Cancer. Cancer Cell Int..

[186] Silva-Pinto P.A., de Pontes J.T.C., Aguilar-Morón B., Canales C.S.C., Pavan F.R., Roque-Borda C.A. (2025). Phytochemical Insights into Flavonoids in Cancer: Mechanisms, Therapeutic Potential, and the Case of Quercetin. Heliyon.

[187] Konecki T., Juszczak A., Cichocki M. (2022). Can Diet Prevent Urological Cancers? An Update on Carotenoids as Chemopreventive Agents. Nutrients.

[188] Sui J., Guo J., Pan D., Wang Y., Xu Y., Sun G., Xia H. (2024). The Efficacy of Dietary Intake, Supplementation, and Blood Concentrations of Carotenoids in Cancer Prevention: Insights from an Umbrella Meta-Analysis. Foods.

[189] Wu S., Liu Y., Michalek J.E., Mesa R.A., Parma D.L., Rodriguez R., Mansour A.M., Svatek R., Tucker T.C., Ramirez A.G. (2020). Carotenoid Intake and Circulating Carotenoids Are Inversely Associated with the Risk of Bladder Cancer: A Dose-Response Meta-Analysis. Adv. Nutr..

[190] Helzlsouer K.J., Comstock G.W., Morris J.S. (1989). Selenium, Lycopene, Alpha-Tocopherol, Beta-Carotene, Retinol, and Subsequent Bladder Cancer. Cancer Res..

[191] Hung R.J., Zhang Z.-F., Rao J.Y., Pantuck A., Reuter V.E., Heber D., Lu Q.-Y. (2006). Protective Effects of Plasma Carotenoids on the Risk of Bladder Cancer. J. Urol..

[192] Michaud D.S., Pietinen P., Taylor P.R., Virtanen M., Virtamo J., Albanes D. (2002). Intakes of Fruits and Vegetables, Carotenoids and Vitamins A, E, C in Relation to the Risk of Bladder Cancer in the ATBC Cohort Study. Br. J. Cancer.

[193] Xu X., Xie B., Li S., Wang S., Xia D., Meng H. (2021). Association of Dietary Tomato Intake with Bladder Cancer Risk in a Prospective Cohort of 101,683 Individuals with 12.5 Years of Follow-Up. Aging.

[194] Okajima E., Ozono S., Endo T., Majima T., Tsutsumi M., Fukuda T., Akai H., Denda A., Hirao Y., Okajima E. (1997). Chemopreventive Efficacy of Piroxicam Administered Alone or in Combination with Lycopene and Beta-Carotene on the Development of Rat Urinary Bladder Carcinoma after N-Butyl-N-(4-Hydroxybutyl)Nitrosamine Treatment. Jpn. J. Cancer Res..

[195] Koçak T., Özbek Y.D., Bodur M., Yeşil S., Ağagündüz D. (2026). Intersection of Precision Nutrition and Bladder Cancer: A Narrative State-of-the-Art Review of Potential Applications and Challenges. J. Clin. Med..

[196] Hsieh J.J., Purdue M.P., Signoretti S., Swanton C., Albiges L., Schmidinger M., Heng D.Y., Larkin J., Ficarra V. (2017). Renal Cell Carcinoma. Nat. Rev. Dis. Prim..

[197] Linehan W.M., Spellman P.T., Ricketts C.J., Creighton C.J., Fei S.S., Davis C., Wheeler D.A., Murray B.A., Schmidt L., Vocke C.D. (2016). Comprehensive Molecular Characterization of Papillary Renal Cell Carcinoma. N. Engl. J. Med..

[198] Coffey N.J., Simon M.C. (2024). Metabolic Alterations in Hereditary and Sporadic Renal Cell Carcinoma. Nat. Rev. Nephrol..

[199] Qu Y., Feng J., Wu X., Bai L., Xu W., Zhu L., Liu Y., Xu F., Zhang X., Yang G. (2022). A Proteogenomic Analysis of Clear Cell Renal Cell Carcinoma in a Chinese Population. Nat. Commun..

[200] Ganti S., Taylor S.L., Abu Aboud O., Yang J., Evans C., Osier M.V., Alexander D.C., Kim K., Weiss R.H. (2012). Kidney Tumor Biomarkers Revealed by Simultaneous Multiple Matrix Metabolomics Analysis. Cancer Res..

[201] Nizioł J., Bonifay V., Ossoliński K., Ossoliński T., Ossolińska A., Sunner J., Beech I., Arendowski A., Ruman T. (2018). Metabolomic Study of Human Tissue and Urine in Clear Cell Renal Carcinoma by LC-HRMS and PLS-DA. Anal. Bioanal. Chem..

[202] Sahin K., Cross B., Sahin N., Ciccone K., Suleiman S., Osunkoya A.O., Master V., Harris W., Carthon B., Mohammad R. (2015). Lycopene in the Prevention of Renal Cell Cancer in the TSC2 Mutant Eker Rat Model. Arch. Biochem. Biophys..

[203] Ciccarese C., Iacovelli R., Porta C., Procopio G., Bria E., Astore S., Cannella M.A., Tortora G. (2021). Efficacy of VEGFR-TKIs plus Immune Checkpoint Inhibitors in Metastatic Renal Cell Carcinoma Patients with Favorable IMDC Prognosis. Cancer Treat. Rev..

[204] Tsai Y.-C., Tsai C.-C., Tsai V.F.S., Lin C.-H., Kuo C.-Y. (2026). Natural Products in Clear Cell Renal Cell Carcinoma: Rewiring the VHL-HIF Axis, Metabolic Plasticity, and Tumor–Immune Interactions. Int. J. Mol. Sci..

[205] Gougis P., Hilmi M., Geraud A., Mir O., Funck-Brentano C. (2021). Potential Cytochrome P450-Mediated Pharmacokinetic Interactions between Herbs, Food, and Dietary Supplements and Cancer Treatments. Crit. Rev. Oncol. Hematol..

[206] Bajalia E.M., Azzouz F.B., Chism D.A., Giansiracusa D.M., Wong C.G., Plaskett K.N., Bishayee A. (2022). Phytochemicals for the Prevention and Treatment of Renal Cell Carcinoma: Preclinical and Clinical Evidence and Molecular Mechanisms. Cancers.

[207] Vazhappilly C.G., Alsawaf S., Mathew S., Nasar N.A., Hussain M.I., Cherkaoui N.M., Ayyub M., Alsaid S.Y., Thomas J.G., Cyril A.C. (2025). Pharmacodynamics and Safety in Relation to Dose and Response of Plant Flavonoids in Treatment of Cancers. Inflammopharmacol..

[208] Feng C., Lyu Y., Gong L., Wang J. (2022). Therapeutic Potential of Natural Products in the Treatment of Renal Cell Carcinoma: A Review. Nutrients.

[209] Chovanec M., Abu Zaid M., Hanna N., El-Kouri N., Einhorn L.H., Albany C. (2017). Long-Term Toxicity of Cisplatin in Germ-Cell Tumor Survivors. Ann. Oncol..

[210] Shen H., Shih J., Hollern D.P., Wang L., Bowlby R., Tickoo S.K., Thorsson V., Mungall A.J., Newton Y., Hegde A.M. (2018). Integrated Molecular Characterization of Testicular Germ Cell Tumors. Cell Rep..

[211] Cayetano-Salazar L., Olea-Flores M., Zuñiga-Eulogio M.D., Weinstein-Oppenheimer C., Fernández-Tilapa G., Mendoza-Catalán M.A., Zacapala-Gómez A.E., Ortiz-Ortiz J., Ortuño-Pineda C., Navarro-Tito N. (2021). Natural Isoflavonoids in Invasive Cancer Therapy: From Bench to Bedside. Phytother. Res..

[212] Imran M., Ghorat F., Ul-Haq I., Ur-Rehman H., Aslam F., Heydari M., Shariati M.A., Okuskhanova E., Yessimbekov Z., Thiruvengadam M. (2020). Lycopene as a Natural Antioxidant Used to Prevent Human Health Disorders. Antioxidants.

[213] Cocetta V., Tinazzi M., Giacomini I., Rosato B., Ragazzi E., Berretta M., Montopoli M. (2023). Clinical Evidence of Interaction between Nutraceutical Supplementation and Platinum-Based Chemotherapy. Curr. Med. Chem..

[214] Dasari S., Njiki S., Mbemi A., Yedjou C.G., Tchounwou P.B. (2022). Pharmacological Effects of Cisplatin Combination with Natural Products in Cancer Chemotherapy. Int. J. Mol. Sci..

[215] Rahimi A., Asadi F., Rezghi M., Kazemi S., Soorani F., Memariani Z. (2022). Natural Products against Cisplatin-Induced Male Reproductive Toxicity: A Comprehensive Review. J. Biochem. Mol. Toxicol..

[216] Abdel-All S.R., Shakour Z.T.A., Abouhussein D.M.N., Reda E., Sallam T.F., El-Hefnawy H.M., Abdel-Monem A.R. (2021). Phytochemical and Biological Evaluation of a Newly Designed Nutraceutical Self-Nanoemulsifying Self-Nanosuspension for Protection and Treatment of Cisplatin Induced Testicular Toxicity in Male Rats. Molecules.

[217] Negm W.A., El-Kadem A.H., Hussein I.A., Alqahtani M.J. (2022). The Mechanistic Perspective of Bilobetin Protective Effects against Cisplatin-Induced Testicular Toxicity: Role of Nrf-2/Keap-1 Signaling, Inflammation, and Apoptosis. Biomedicines.

[218] Zarjani A.K., Khorsandi L., Kahkesh M.H., Nejad D.B., Lafout F.M., Khodayar M.J., Ashtari A. (2026). Protective Effect of Naringenin on Cisplatin-Testicular Damage through the Oxidation and P38 MAPK Inflammatory Pathway. JBRA Assist. Reprod..

[219] Majumdar A., Saraf S., Rao S.P. (2024). Current Trends in Herbal Medicines Targeting to Renal Cell Metabolic Pathways in the Treatment of Cancer. Pharmacol. Res.-Nat. Prod..

[220] Wang J., Chen Z., Liu W., Xu Z., Liu H., Li Y., Yan Y. (2025). Harnessing Plant-Derived Natural Compounds to Target Ferroptosis, Pyroptosis, Immune Modulation and Renin-Angiotensin System in Renal Cell Carcinoma. J. Renin Angiotensin Aldosterone Syst..

[221] Qari M., Harakeh S., Akefe I.O., Saber S.H., Al-Raddadi R., Elmageed Z.Y.A., Alamri T., El-Shitany N., Ali S.S., Almuhayawi M.S. (2023). Pomegranate Nanoparticle Mitigates Cisplatin-Induced Testicular Toxicity and Improves Cisplatin Anti-Cancer Efficacy in Ehrlich Carcinoma Model. J. King Saud. Univ. Sci..

[222] Chyuan I.-T., Chu C.-L., Hsu P.-N. (2021). Targeting the Tumor Microenvironment for Improving Therapeutic Effectiveness in Cancer Immunotherapy: Focusing on Immune Checkpoint Inhibitors and Combination Therapies. Cancers.

[223] Giannone G., Ghisoni E., Genta S., Scotto G., Tuninetti V., Turinetto M., Valabrega G. (2020). Immuno-Metabolism and Microenvironment in Cancer: Key Players for Immunotherapy. Int. J. Mol. Sci..

[224] Gill G.S., Kharb S., Goyal G., Das P., Kurdia K.C., Dhar R., Karmakar S. (2025). Immune Checkpoint Inhibitors and Immunosuppressive Tumor Microenvironment: Current Challenges and Strategies to Overcome Resistance. Immunopharmacol. Immunotoxicol..

[225] Naimi A., Mohammed R.N., Raji A., Chupradit S., Yumashev A.V., Suksatan W., Shalaby M.N., Thangavelu L., Kamrava S., Shomali N. (2022). Tumor Immunotherapies by Immune Checkpoint Inhibitors (ICIs); the Pros and Cons. Cell Commun. Signal..

[226] Masuelli L., Benvenuto M., Focaccetti C., Ciuffa S., Fazi S., Bei A., Miele M.T., Piredda L., Manzari V., Modesti A. (2021). Targeting the Tumor Immune Microenvironment with “Nutraceuticals”: From Bench to Clinical Trials. Pharmacol. Ther..

[227] Yuan X., Duan Y., Xiao Y., Sun K., Qi Y., Zhang Y., Ahmed Z., Moiani D., Yao J., Li H. (2022). Vitamin E Enhances Cancer Immunotherapy by Reinvigorating Dendritic Cells via Targeting Checkpoint SHP1. Cancer Discov..

[228] Kawanabe-Matsuda H., Takeda K., Nakamura M., Makino S., Karasaki T., Kakimi K., Nishimukai M., Ohno T., Omi J., Kano K. (2022). Dietary Lactobacillus-Derived Exopolysaccharide Enhances Immune-Checkpoint Blockade Therapy. Cancer Discov..

[229] Yang J., Yang X., Pan W., Wang M., Lu Y., Zhang J., Fang Z., Zhang X., Ji Y., Bei J.-X. (2021). Fucoidan-Supplemented Diet Potentiates Immune Checkpoint Blockage by Enhancing Antitumor Immunity. Front. Cell Dev. Biol..

[230] Zhang J., Wang S., Guo X., Lu Y., Liu X., Jiang M., Li X., Qin B., Luo Z., Liu H. (2022). Arginine Supplementation Targeting Tumor-Killing Immune Cells Reconstructs the Tumor Microenvironment and Enhances the Antitumor Immune Response. ACS Nano.

[231] Spencer C.N., McQuade J.L., Gopalakrishnan V., McCulloch J.A., Vetizou M., Cogdill A.P., Khan M.A.W., Zhang X., White M.G., Peterson C.B. (2021). Dietary Fiber and Probiotics Influence the Gut Microbiome and Melanoma Immunotherapy Response. Science.

[232] Chen J., Gao Y., Chen Y., Wang Q., Zhang Y., Huang Y., Xian X., Zhou D., Zhou H., Liu R. (2025). Identification and Validation of Intratumoral Microbiome Associated with Sensitization to Immune Checkpoint Inhibitors. Cell Rep. Med..

[233] Somodi C., Dora D., Horváth M., Szegvari G., Lohinai Z. (2025). Gut Microbiome Changes and Cancer Immunotherapy Outcomes Associated with Dietary Interventions: A Systematic Review of Preclinical and Clinical Evidence. J. Transl. Med..

[234] Murphy S., Rahmy S., Gan D., Liu G., Zhu Y., Manyak M., Duong L., He J., Schofield J.H., Schafer Z.T. (2024). Ketogenic Diet Alters the Epigenetic and Immune Landscape of Prostate Cancer to Overcome Resistance to Immune Checkpoint Blockade Therapy. Cancer Res..

[235] Jannati S., Patel A., Patnaik R., Banerjee Y. (2025). Oleocanthal as a Multifunctional Anti-Cancer Agent: Mechanistic Insights, Advanced Delivery Strategies, and Synergies for Precision Oncology. Int. J. Mol. Sci..

[236] Tang S., Li B. (2026). Editorial: Novel Therapeutics for Urological Cancers. Front. Pharmacol..

[237] Ashrafpour S., Ashrafpour M. (2025). The Double-Edged Sword of Nutraceuticals: Comprehensive Review of Protective Agents and Their Hidden Risks. Front. Nutr..

[238] Aggarwal P., Dutta G., Shaurya R., Rajendran V., Thirunavukkarasu P., Charan J., Kumar T. (2025). Unlocking the Potential of Nutraceuticals in Cancer Chemotherapy: A Comprehensive Review. Cureus.

[239] Zhang Y., Zhang S., Sun H., Xu L. (2025). The Pathogenesis and Therapeutic Implications of Metabolic Reprogramming in Renal Cell Carcinoma. Cell Death Discov..

[240] D’Archivio M., Filesi C., Varì R., Scazzocchio B., Masella R. (2010). Bioavailability of the Polyphenols: Status and Controversies. Int. J. Mol. Sci..

[241] Sun Q., He M., Zhang M., Zeng S., Chen L., Zhou L., Xu H. (2020). Ursolic Acid: A Systematic Review of Its Pharmacology, Toxicity and Rethink on Its Pharmacokinetics Based on PK-PD Model. Fitoterapia.

[242] Mondul A.M., Weinstein S.J., Layne T.M., Albanes D. (2017). Vitamin D and Cancer Risk and Mortality: State of the Science, Gaps, and Challenges. Epidemiol. Rev..

[243] Shah K., Secrest M.H., Zhou W., Wang S., Canter D., Zhang Y., Jin D., Sokol E., Nowicka M., Ang Houle A. (2025). Clinical Context Shapes the Relationship between Genomic Alterations and Response to AR Inhibitors and Chemotherapy in Metastatic Prostate Cancer. Clin. Cancer Res..

[244] Brezmes J., Llambrich M., Cumeras R., Gumà J. (2022). Urine NMR Metabolomics for Precision Oncology in Colorectal Cancer. Int. J. Mol. Sci..

[245] Ong E.S. (2024). Urine Metabolites and Bioactive Compounds from Functional Food: Applications of Liquid Chromatography Mass Spectrometry. Crit. Rev. Anal. Chem..

[246] Kim H.K., Kim S.J., Gil W.J., Yang C.-S. (2025). Exploring the Therapeutic Potential of Phytochemicals: Challenges and Strategies for Clinical Translation. Phytomedicine.

[247] Aljabali A.A.A., Obeid M.A., Bashatwah R.M., Qnais E., Gammoh O., Alqudah A., Mishra V., Mishra Y., Khan M.A., Parvez S. (2025). Phytochemicals in Cancer Therapy: A Structured Review of Mechanisms, Challenges, and Progress in Personalized Treatment. Chem. Biodivers..

[248] Iga K., Kiriyama A. (2024). Interplay of UDP-Glucuronosyltransferase and CYP2C8 for CYP2C8 Mediated Drug Oxidation and Its Impact on Drug–Drug Interaction Produced by Standardized CYP2C8 Inhibitors, Clopidogrel and Gemfibrozil. Clin. Pharmacokinet..

[249] Lee J., Beers J.L., Geffert R.M., Jackson K.D. (2024). A Review of CYP-Mediated Drug Interactions: Mechanisms and In Vitro Drug-Drug Interaction Assessment. Biomolecules.

[250] Olubamiwa A.O., Liao T.-J., Zhao J., Dehanne P., Noban C., Angin Y., Barberan O., Chen M. (2025). Drug Interaction with UDP-Glucuronosyltransferase (UGT) Enzymes Is a Predictor of Drug-Induced Liver Injury. Hepatology.

[251] Breedveld P., Beijnen J.H., Schellens J.H.M. (2006). Use of P-Glycoprotein and BCRP Inhibitors to Improve Oral Bioavailability and CNS Penetration of Anticancer Drugs. Trends Pharmacol. Sci..

[252] Choi Y.H., Yu A.-M. (2014). ABC Transporters in Multidrug Resistance and Pharmacokinetics, and Strategies for Drug Development. Curr. Pharm. Des..

[253] Poondru S., Ghicavii V., Khosravan R., Manchandani P., Heo N., Moy S., Wojtkowski T., Patton M., Haas G.P. (2022). Effect of Enzalutamide on PK of P-Gp and BCRP Substrates in Cancer Patients: CYP450 Induction May Not Always Predict Overall Effect on Transporters. CTS.

[254] Fogli S., Porta C., Del Re M., Crucitta S., Gianfilippo G., Danesi R., Rini B.I., Schmidinger M. (2020). Optimizing Treatment of Renal Cell Carcinoma with VEGFR-TKIs: A Comparison of Clinical Pharmacology and Drug-Drug Interactions of Anti-Angiogenic Drugs. Cancer Treat. Rev..

[255] Narayan V., Liu T., Song Y., Mitchell J., Sicks J., Gareen I., Sun L., Denduluri S., Fisher C., Manikowski J. (2023). Early Increases in Blood Pressure and Major Adverse Cardiovascular Events in Patients with Renal Cell Carcinoma and Thyroid Cancer Treated with VEGFR TKIs. J. Natl. Compr. Canc. Netw..

[256] Sayegh N., Yirerong J., Agarwal N., Addison D., Fradley M., Cortes J., Weintraub N.L., Sayed N., Raval G., Guha A. (2023). Cardiovascular Toxicities Associated with Tyrosine Kinase Inhibitors. Curr. Cardiol. Rep..

[257] Zhang Y., Deng J., Wang J. (2025). Cardiovascular Toxicities Associated with Vascular Endothelial Growth Factor Receptor Tyrosine Kinase Inhibitors: A Pharmacovigilance Study Based on FDA Adverse Event Reporting System. Int. J. Clin. Pharm..

[258] Van Daele M., Kilpatrick L.E., Woolard J., Hill S.J. (2023). Characterisation of Tyrosine Kinase Inhibitor-Receptor Interactions at VEGFR2 Using Sunitinib-Red and nanoBRET. Biochem. Pharmacol..

[259] Franczyk B., Rysz J., Ławiński J., Ciałkowska-Rysz A., Gluba-Brzózka A. (2023). Cardiotoxicity of Selected Vascular Endothelial Growth Factor Receptor Tyrosine Kinase Inhibitors in Patients with Renal Cell Carcinoma. Biomedicines.

[260] McTigue M., Murray B.W., Chen J.H., Deng Y.-L., Solowiej J., Kania R.S. (2012). Molecular Conformations, Interactions, and Properties Associated with Drug Efficiency and Clinical Performance among VEGFR TK Inhibitors. Proc. Natl. Acad. Sci. USA.

[261] Reinbold H., Goebell P.J., Merseburger A.S. (2026). Arzneimittelinteraktionen als Herausforderung bei der Monotherapie des fortgeschrittenen oder metastasierten klarzelligen Nierenzellkarzinoms mit VEGFR-assoziierten Tyrosinkinase-Inhibitoren. Aktuelle Urol..

[262] Grice S., Olsson-Brown A., Naisbitt D.J., Hammond S. (2024). Immunological Drug-Drug Interactions Affect the Efficacy and Safety of Immune Checkpoint Inhibitor Therapies. Chem. Res. Toxicol..

[263] Duan M., Leng S., Mao P. (2024). Cisplatin in the Era of PARP Inhibitors and Immunotherapy. Pharmacol. Ther..

[264] Belderbos B.P.S., Bins S., van Leeuwen R.W.F., Oomen-de Hoop E., van der Meer N., de Bruijn P., Hamberg P., Overkleeft E.N.M., van der Deure W.M., Lolkema M.P. (2018). Influence of Enzalutamide on Cabazitaxel Pharmacokinetics: A Drug-Drug Interaction Study in Metastatic Castration-Resistant Prostate Cancer (mCRPC) Patients. Clin. Cancer Res..

[265] Powell N.R., Shugg T., Ly R.C., Albany C., Radovich M., Schneider B.P., Skaar T.C. (2021). Life-Threatening Docetaxel Toxicity in a Patient with Reduced-Function CYP3A Variants: A Case Report. Front. Oncol..

[266] Bruin M.A.C., Sonke G.S., Beijnen J.H., Huitema A.D.R. (2022). Pharmacokinetics and Pharmacodynamics of PARP Inhibitors in Oncology. Clin. Pharmacokinet..

[267] Zeng Y., Arisa O., Peer C.J., Fojo A., Figg W.D. (2024). PARP Inhibitors: A Review of the Pharmacology, Pharmacokinetics, and Pharmacogenetics. Semin. Oncol..

[268] Franzese O., Graziani G. (2022). Role of PARP Inhibitors in Cancer Immunotherapy: Potential Friends to Immune Activating Molecules and Foes to Immune Checkpoints. Cancers.

[269] Li A., Yi M., Qin S., Chu Q., Luo S., Wu K. (2019). Prospects for Combining Immune Checkpoint Blockade with PARP Inhibition. J. Hematol. Oncol..

[270] Stewart R.A., Pilié P.G., Yap T.A. (2018). Development of PARP and Immune-Checkpoint Inhibitor Combinations. Cancer Res..

[271] Lundstam S., Haraldsson B., Nystrom J., Yachnin J. (2024). A Phase I/II, Open Label, Single Arm Study on Safety, Tolerability and Anti-Tumour Effiacy of Orellanine Treatment in Patients with Metastatic Clear-Cell or Papillary Renal Cell Carcinoma. J. Clin. Oncol..

[272] Bobkova T., Bobkov A., Li Y. (2025). Pharmacological Inhibition of the PI3K/AKT/mTOR Pathway in Rheumatoid Arthritis Synoviocytes: A Systematic Review and Meta-Analysis (Preclinical). Pharmaceuticals.

[273] Merecz-Sadowska A., Sadowski A., Zielińska-Bliźniewska H., Zajdel K., Zajdel R. (2025). Network Pharmacology as a Tool to Investigate the Antioxidant and Anti-Inflammatory Potential of Plant Secondary Metabolites—A Review and Perspectives. Int. J. Mol. Sci..

[274] Baston C., Preda A., Iordache A., Olaru V., Surcel C., Sinescu I., Gingu C. (2024). How to Integrate Prostate Cancer Biomarkers in Urology Clinical Practice: An Update. Cancers.

[275] Min K., Lin Q., Qiu D. (2025). Precision Medicine in Prostate Cancer: Individualized Treatment through Radiomics, Genomics, and Biomarkers. Cancer Imaging..

[276] Mizuno K., Beltran H. (2022). Future Directions for Precision Oncology in Prostate Cancer. Prostate.

[277] Armstrong A.J., Morris M.J., Abida W., Aggarwal R.R., Antonarakis E.S., Attard G., Beltran H., Bryce A., Carducci M.A., Cheng H.H. (2026). Trial Design and Objectives for Patients with Prostate Cancer: Recommendations from the Prostate Cancer Working Group 4. J. Clin. Oncol..

[278] Eggener S.E., Rumble R.B., Armstrong A.J., Morgan T.M., Crispino T., Cornford P., van der Kwast T., Grignon D.J., Rai A.J., Agarwal N. (2020). Molecular Biomarkers in Localized Prostate Cancer: ASCO Guideline. J. Clin. Oncol..

[279] Mohanty S.K., Lobo A., Mishra S.K., Cheng L. (2023). Precision Medicine in Bladder Cancer: Present Challenges and Future Directions. J. Pers. Med..

[280] Sanguedolce F., Zanelli M., Palicelli A., Ascani S., Zizzo M., Cocco G., Björnebo L., Lantz A., Falagario U.G., Cormio L. (2022). Are We Ready to Implement Molecular Subtyping of Bladder Cancer in Clinical Practice? Part 1: General Issues and Marker Expression. Int. J. Mol. Sci..

[281] Cimadamore A., Franzese C., Loreto C.D., Blanca A., Lopez-Beltran A., Crestani A., Giannarini G., Tan P.H., Carneiro B.A., El-Deiry W.S. (2024). Predictive and Prognostic Biomarkers in Urological Tumours. Pathology.

[282] Li W., Meng J., Yang H., Zhang T., Yin L. (2026). Editorial: Genomic Discoveries and Pharmaceutical Development in Urologic Tumors—Volume II. Front. Pharmacol..

[283] Esposito F., De Martino M., Franco V., Fusco A., Chieffi P. (2025). Potential Therapeutic Targets and Biomarkers in Testicular Germ Cell Tumor Oncogenesis. Expert Opin. Ther. Targets.

[284] Sykes J., Kaldany A., Jang T.L. (2024). Current and Evolving Biomarkers in the Diagnosis and Management of Testicular Germ Cell Tumors. J. Clin. Med..

