# Supplementary material for: Nutraceuticals in Uro-Oncology: A Structured Expert Review and Precision-Oriented Framework
Source: Nutrients. 2026 Jul 23;18(15):2411. doi: 10.3390/nu18152411 (PMC13468118; doi:10.3390/nu18152411)
Supplement: Supplementary file 1 [file nutrients-18-02411-s001.zip › nutrients-4413930-supplementary.pdf]

Supplementary Tables

Supplementary Table S1. Database-specific search strategies used for the structured expert review

Database	Date coverage	Search date	Search string
PubMed/MEDLINE	Inception to 17 June 2026	17 June 2026	("prostate cancer" OR "prostatic neoplasm*" OR "bladder cancer" OR "urothelial carcinoma" OR "renal cell carcinoma" OR "kidney cancer" OR "testicular cancer" OR "testicular germ cell tumor*") AND (nutraceutical* OR phytochemical* OR polyphenol* OR flavonoid* OR carotenoid* OR isoflavone* OR catechin* OR curcumin OR resveratrol OR quercetin OR genistein OR daidzein OR equol OR lycopene OR "epigallocatechin gallate" OR EGCG OR "ursolic acid" OR fisetin OR kaempferol OR myricetin) AND (signaling OR pathway* OR "androgen receptor" OR PI3K OR AKT OR mTOR OR MAPK OR "NF-kappaB" OR STAT OR FGFR OR EGFR OR VEGF OR HIF OR "DNA damage response" OR apoptosis OR angiogenesis OR inflammation OR "epithelial-mesenchymal transition" OR microenvironment OR pharmacokinetic* OR bioavailability OR biomarker* OR safety OR interaction*)
Embase	Inception to 17 June 2026	17 June 2026	('prostate cancer' OR 'prostatic neoplasm' OR 'bladder cancer' OR 'urothelial carcinoma' OR 'renal cell carcinoma' OR 'kidney cancer' OR 'testicular cancer' OR 'testicular germ cell tumor') AND ('prostate cancer' OR 'prostatic neoplasm' OR 'bladder cancer' OR 'urothelial carcinoma' OR 'renal cell carcinoma' OR 'kidney cancer' OR 'testicular cancer' OR 'testicular germ cell tumor') AND (nutraceutical* OR phytochemical* OR polyphenol* OR flavonoid* OR carotenoid* OR isoflavone* OR catechin* OR curcumin OR resveratrol OR quercetin OR genistein OR daidzein OR equol OR lycopene OR 'epigallocatechin gallate' OR EGCG OR 'ursolic acid' OR fisetin OR kaempferol OR myricetin) AND (signaling OR pathway* OR 'androgen receptor' OR PI3K OR AKT OR mTOR OR MAPK OR 'NF-kappaB' OR STAT OR FGFR OR EGFR OR VEGF OR HIF OR 'DNA damage response' OR apoptosis OR angiogenesis OR inflammation OR 'epithelial-mesenchymal transition' OR microenvironment OR pharmacokinetic* OR bioavailability OR biomarker* OR safety OR interaction*)
Scopus	Inception to 17 June 2026	17 June 2026	TITLE-ABS-KEY (("prostate cancer" OR "prostatic neoplasm*" OR "bladder cancer" OR "urothelial carcinoma" OR "renal cell carcinoma" OR "kidney

			cancer" OR "testicular cancer" OR "testicular germ cell tumor*") AND (nutraceutical* OR phytochemical* OR polyphenol* OR flavonoid* OR carotenoid* OR isoflavone* OR catechin* OR curcumin OR resveratrol OR quercetin OR genistein OR daidzein OR equol OR lycopene OR "epigallocatechin gallate" OR EGCG OR "ursolic acid" OR fisetin OR kaempferol OR myricetin) AND (signaling OR pathway* OR "androgen receptor" OR PI3K OR AKT OR mTOR OR MAPK OR "NF-kappaB" OR STAT OR FGFR OR EGFR OR VEGF OR HIF OR "DNA damage response" OR apoptosis OR angiogenesis OR inflammation OR "epithelial-mesenchymal transition" OR microenvironment OR pharmacokinetic* OR bioavailability OR biomarker* OR safety OR interaction*))
Web of Science Core Collection	Inception to 17 June 2026	17 June 2026	TS=(("prostate cancer" OR "prostatic neoplasm*" OR "bladder cancer" OR "urothelial carcinoma" OR "renal cell carcinoma" OR "kidney cancer" OR "testicular cancer" OR "testicular germ cell tumor*") AND (nutraceutical* OR phytochemical* OR polyphenol* OR flavonoid* OR carotenoid* OR isoflavone* OR catechin* OR curcumin OR resveratrol OR quercetin OR genistein OR daidzein OR equol OR lycopene OR "epigallocatechin gallate" OR EGCG OR "ursolic acid" OR fisetin OR kaempferol OR myricetin) AND (signaling OR pathway* OR "androgen receptor" OR PI3K OR AKT OR mTOR OR MAPK OR "NF-kappaB" OR STAT OR FGFR OR EGFR OR VEGF OR HIF OR "DNA damage response" OR apoptosis OR angiogenesis OR inflammation OR "epithelial-mesenchymal transition" OR microenvironment OR pharmacokinetic* OR bioavailability OR biomarker* OR safety OR interaction*))

Supplementary Table S2 Database-specific search strategies used in PubMed/MEDLINE, Embase, Scopus, and Web of Science Core Collection.

Database	Coverage date	Search date	Complete search string	Filters
PubMed/MEDLINE	Inception–17 June 2026	Actual date	Full PubMed query	English; humans/animals not restricted
Embase	Inception–17 June 2026	Actual date	Emtree + free-text query	Article/review as applicable
Scopus	Inception–17 June 2026	Actual date	TITLE-ABS-KEY query	English
Web of Science	Inception–17 June 2026	Actual date	TS query	English
